# Production of secondary metabolites in stirred tank bioreactor co-cultures of *Streptomyces noursei* and *Aspergillus terreus*

**DOI:** 10.3389/fbioe.2022.1011220

**Published:** 2022-09-29

**Authors:** Tomasz Boruta, Anna Ścigaczewska, Marcin Bizukojć

**Affiliations:** Department of Bioprocess Engineering, Faculty of Process and Environmental Engineering, Lodz University of Technology, Lodz, Poland

**Keywords:** *Aspergillus terreus*, co-culture, lovastatin, nystatin, *Streptomyces noursei*

## Abstract

The focus of the study was to characterize the bioprocess kinetics and secondary metabolites production in the novel microbial co-cultivation system involving *Streptomyces noursei* ATCC 11455 (the producer of an antifungal substance known as nystatin) and *Aspergillus terreus* ATCC 20542 (the source of lovastatin, a cholesterol-lowering drug). The investigated “*A. terreus* vs. *S. noursei*” stirred tank bioreactor co-cultures allowed for the concurrent development and observable biosynthetic activity of both species. In total, the production profiles of 50 secondary metabolites were monitored over the course of the study. The co-cultures were found to be effective in terms of enhancing the biosynthesis of several metabolic products, including mevinolinic acid, an acidic form of lovastatin. This work provided a methodological example of assessing the activity of a given strain in the co-culture by using the substrates which can be metabolized exclusively by this strain. Since *S. noursei* was shown to be incapable of lactose utilization, the observed changes in lactose levels were attributed to *A. terreus* and thus confirmed its viability. The study was complemented with the comparative microscopic observations of filamentous morphologies exhibited in the co-cultures and corresponding monocultures.

## 1 Introduction

Actinomycetes and filamentous fungi are potent sources of bioactive secondary metabolites (also termed natural products or specialized metabolites) that can be potentially used as drug leads in the pharmaceutical industry. The discovery of novel secondary metabolites is greatly stimulated by the isolation of new microbial strains and, most importantly, by the development of non-standard microbiological techniques allowing the scientists to uncover the biosynthetic potential hidden within the microbial metabolic pathways (Du and van Wezel, 2018; Deng et al., 2020). One of the most promising and effective strategies applied in this context is to mimic the naturally occurring microbial interactions through the laboratory co-cultivation of two or more species. Since the environmental signals required to activate the production of secondary metabolites are not always existent in the monoculture (also referred to as the axenic culture), performing the co-culture experiments can be used to broaden the perspective on the metabolic capabilities of the investigated microbes. Importantly, the co-cultivation may lead to the increased (or decreased) titer of a given metabolite compared to the value recorded for a monoculture or, in some cases, visibly alter the exhibited repertoire of secondary metabolic products (Hoshino et al., 2019; Arora et al., 2020; Liu and Kakeya, 2020; Kim et al., 2021; Schlembach et al., 2021; Knowles et al., 2022).

The production of secondary metabolites in the microbial co-cultures is typically studied at a relatively small scale, i.e., with the use of laboratory plates or flasks, while the bioreactor-based studies are still rare. In the case of *Streptomyces*, a major actinomycete genus, the stirred tank bioreactor system was used by Luti and Mavituna (2011) to demonstrate the elicitation of undecylprodigiosin biosynthesis in *Streptomyces coelicolor* as a result of co-cultivation with *E. coli*. Recently, the bioreactor co-cultures involving *Streptomyces rimosus*, the producer of an antibiotic oxytetracycline (Slemc et al., 2021), and *Aspergillus terreus*, the fungal producer of a cholesterol-lowering drug lovastatin (Barrios-González et al., 2020), were characterized by our group with regard to a broad spectrum of secondary metabolites and cultivation conditions (Boruta et al., 2021). The study demonstrated the stimulatory effects exerted by *A. terreus* on *S. rimosus* (and vice versa), including the induced formation of rimocidin and milbemycin derivates. It is a good example of a co-cultivation system that favors a single species, i.e., the one that dominates its partner through faster growth and the production of bioactive secondary metabolites. The “*S. rimosus* vs *A. terreus*” co-culture was observed to be a “microbial war”, in which there was a strong tendency for only a single species to prevail by the ultimate “defeat” of the accompanying microbe, depending on the applied inoculation scheme and bioprocess conditions. Specifically, it was recorded that *S. rimosus* dominated *A. terreus* in all the co-cultures initiated by the simultaneous inoculation of the two species. This outcome was reversed if the fungus was given a 24-h growth advantage over the actinomycete. Since the work of Boruta et al. (2021) concerned only a single pair of microorganisms, it remained unknown if the “*Aspergillus* vs. *Streptomyces*” bioreactor co-cultures could possibly follow a different scenario built around the co-existence of two active strains rather than the elimination of the “loser” by the “winner”. In the present study, *A. terreus* was co-cultivated with *S. noursei*, an actinomycete recognized primarily for its ability to produce nystatin A1, an important and widely used antifungal drug (Fjærvik and Zotchev, 2005). Since *S. noursei* was known to be easily dominated by *S. rimosus* in submerged co-cultures unless provided with a growth-related advantage (Boruta and Ścigaczewska, 2021), the outcome of the “*S. noursei* vs *A. terreus*” clash could not be easily predicted. The main question was whether *S. noursei* had the biosynthetic and growth-related capabilities to dominate *A. terreus* in the co-culture. Moreover, it remained unknown whether the presence of *A. terreus* would stimulate *S. noursei* to elevate the levels of nystatin A1, a molecule that potentially could be used by this actinomycete as a chemical weapon against the fungal rival. Finally, it has never been investigated if the “*S. noursei* vs. *A. terreus*” co-culture showed any promise towards achieving a microbial two-species system in which both microorganisms display observable growth and biosynthetic activity.

The main objective of the present study was to characterize the stirred tank bioreactor co-cultures of *A. terreus* ATCC 20542 and *S. noursei* ATCC 11455 in terms of bioprocess kinetics and the production of secondary metabolites.

## 2 Materials and methods

### 2.1 Strains

*Streptomyces noursei* ATCC 11455 and *Aspergillus terreus* ATCC 20542 were used throughout this work.

### 2.2 Media

For the preparation of agar slants with *A. terreus* the following composition of solid medium was employed: malt extract (20 g L^−1^), casein peptone (5 g L^−1^), and agar (20 g L^−1^). To prepare the agar slants for *S. noursei* the solid ISP Medium 2 (BD, United States) was prepared according to the manufacturer’s instructions.

The composition of the liquid medium used for the propagation of preculture was the same as the composition of the liquid medium applied in the corresponding bioreactor mono- and co-cultures within a given ATSN experiment.

All media used in the bioreactor mono- and co-cultures contained lactose 20 g L^−1^, yeast extract 4 g L^−1^, KH_2_PO_4_ 1.51 g L^−1^, MgSO_4_·7H_2_O 0.5 g L^−1^, NaCl 0.4 g L^−1^, biotin 0.04 mg L^−1^, and 1 ml L^−1^ of trace elements solution of the following composition: MnSO_4_ 50 mg L^−1^, ZnSO_4_·7H_2_O 1 g L^−1^, Fe(NO_3_)_3_·9H_2_O 2 g L^−1^, Na_2_B_4_O_7_·10H_2_O 100 mg L^−1^, CuSO_4_·5H_2_O 250 mg L^−1^ and Na_2_MoO_4_·2H_2_O 50 mg L^−1^. In the ATSN4, ATSN5, ATSN7, and ATSN8 experiments glucose (20 g L^−1^) was also added to the medium. By contrast, the media used in ATSN3 and ATSN6 did not contain glucose.

The pH of all media was adjusted to the initial value of 6.5 with the use of 0.4 M solution of potassium and sodium carbonates prior to the start of the cultivation process. Once the cultivation started, there was no pH correction over the course of cultivation. All media were sterilized at 121°C in the autoclave. During all bioreactor cultivations, the sterile Antifoam Y-30 Emulsion (Sigma-Aldrich, United States) was employed to prevent foaming.

### 2.3 Cultivation conditions

The agar slants were prepared in glass tubes to obtain the spores required to inoculate the liquid media in preculture shake flasks (as in ATSN5, ATSN6, ATSN7, and ATSN8) or bioreactors (as in ATSN3 and ATSN4). The cultivation leading to the development of spores was carried out for 10 days.

Shake flask precultures were applied to inoculate the bioreactors in ATSN5, ATSN6, ATSN7, and ATSN8 experiments. To start the preculture, the spores were removed from the agar slant with the use of 1 ml disposable sterile pipette and transferred into the 500-ml flat-bottom preculture flask contaning sterile medium (working volume: 150 ml). The procedure was designed and monitored with the use of a Thoma chamber to achieve approx. 10^9^ spores per 1 L of medium (dilutions with sterile medium were performed if necessary to reach the desired concentration of spores). The precultures were shaken for 24 h at 28°C in a Certomat^®^ BS-1 rotary shaker (Sartorius Stedim, Germany) at 110 rotations per minute.

To start the bioreactor run, a 300 ml of each preculture or spore suspension was pumped into the bioreactor from the bottles equipped with tubings. In each ATSN experiment, there were three bioreactors operating in parallel. One of them corresponded to the “*S. noursei* vs. *A. terreus*” co-culture, whereas the remaining two bioreactors hosted the monocultures of *S. noursei* and *A. terreus*, respectively. The details regarding the inoculation strategies applied in the study are presented in Table 1. The initial working volume was equal to 5.5 L. The level of dissolved oxygen was automatically controlled at 20% by adjusting the stirring speed and air flow rate within the intervals 220–300 min^−1^ and 1.5–5.5 L min^−1^, respectively.

**TABLE 1:** Inoculation strategies used in the study.

Experimental run	Variant in the run	Type and volume of inoculum	Time of inoculation of *S. noursei*	Time of inoculation of *A. terreus*
ATSN3	*A. terreus* monoculture (bioreactor #1)	*A. terreus* spore suspension (300 ml)	-	0 h
	*S. noursei* monoculture (bioreactor #2)	*S. noursei* spore suspension (300 ml)	0 h	-
	*A. terreus* + *S. noursei* co-culture (bioreactor #3)	*S. noursei* spore suspension (300 ml) + *A. terreus* spore suspension (300 ml)	0 h	0 h
ATSN4	*A. terreus* monoculture (bioreactor #1)	*A. terreus* spore suspension (300 ml)	-	0 h
	*S. noursei* monoculture (bioreactor #2)	*S. noursei* spore suspension (300 ml)	0 h	-
	*A. terreus* + *S. noursei* co-culture (bioreactor #3)	*S. noursei* spore suspension (300 ml) + *A. terreus* spore suspension (300 ml)	0 h	0 h
ATSN5	*A. terreus* monoculture (bioreactor #1)	*A. terreus* preculture (300 ml)	-	0 h
	*S. noursei* monoculture (bioreactor #2)	*S. noursei* preculture (300 ml)	0 h	-
	*A. terreus* + *S. noursei* co-culture (bioreactor #3)	*S. noursei* preculture (300 ml) + *A. terreus* preculture (300 ml)	0 h	0 h
ATSN6	*A. terreus* monoculture (bioreactor #1)	*A. terreus* preculture (300 ml)	-	0 h
	*S. noursei* monoculture (bioreactor #2)	*S. noursei* preculture (300 ml)	0 h	-
	*A. terreus* + *S. noursei* co-culture (bioreactor #3)	*S. noursei* preculture (300 ml) + *A. terreus* preculture (300 ml)	0 h	0 h
ATSN7	*A. terreus* monoculture (bioreactor #1)	*A. terreus* preculture (300 ml)	-	24 h
	*S. noursei* monoculture (bioreactor #2)	*S. noursei* preculture (300 ml)	0 h	-
	*A. terreus* + *S. noursei* co-culture (bioreactor #3)	*S. noursei* preculture (300 ml) + *A. terreus* preculture (300 ml)	0 h	24 h
ATSN8	*A. terreus* monoculture (bioreactor #1)	*A. terreus* preculture (300 ml)	-	0 h
	*S. noursei* monoculture (bioreactor #2)	*S. noursei* preculture (300 ml)	24 h	-
	*A. terreus* + *S. noursei* co-culture (bioreactor #3)	*S. noursei* preculture (300 ml) + *A. terreus* preculture (300 ml)	24 h	0 h

### 2.4 Analytical procedures

The analysis of secondary metabolites and carbohydrates was performed with the use of ultra-performance liquid chromatography coupled with high-resolution mass spectrometry system ACQUITY-SYNAPT G2 (Waters, United States) as previously described (Boruta et al., 2021). Mevinolinic acid (β-hydroxy acidic form of lovastatin) and nystatin A1 were analyzed quantitatively with the use of authentic standards, whereas the remaining metabolites were determined semi-quantitatively based on the peak areas of [M + H]^+^ or [M−H]^−^ ions. TargetLynx (Waters, United States) software was used in all quantitative and semi-quantitative analyses. The identity of mevinolinic acid, nystatin A1, (+)-geodin, and butyrolactone I was confirmed with the use of standards. For the remaining metabolites, literature records and databases were consulted during the identification process, as described in the previous work (Boruta et al., 2021). In addition, the ACD/MS Fragmenter software (ACD/Labs, Canada) was employed to analyze the possible fragmentation patterns of molecules and thus identify the secondary metabolites.

Microscopic images were taken with the use of Olympus BX53 light microscope equipped with the Olympus cellSens Dimension Desktop 1.16 software (Olympus Corporation, Japan).

### 2.5 Calculations

Glucose and lactose volumetric uptake rates r_GLU_ and r_LAC_ were calculated as follows. The cubic b-spline approximation of the experimental glucose and lactose concentration data was performed. Next, the obtained smooth concentration curves were differentiated in time to find the temporal changes of r_GLU_ and r_LAC_. The calculations were performed by using PTC Mathcad 15 software.

### 2.6 Statistical analysis

**FIGURE 1:**
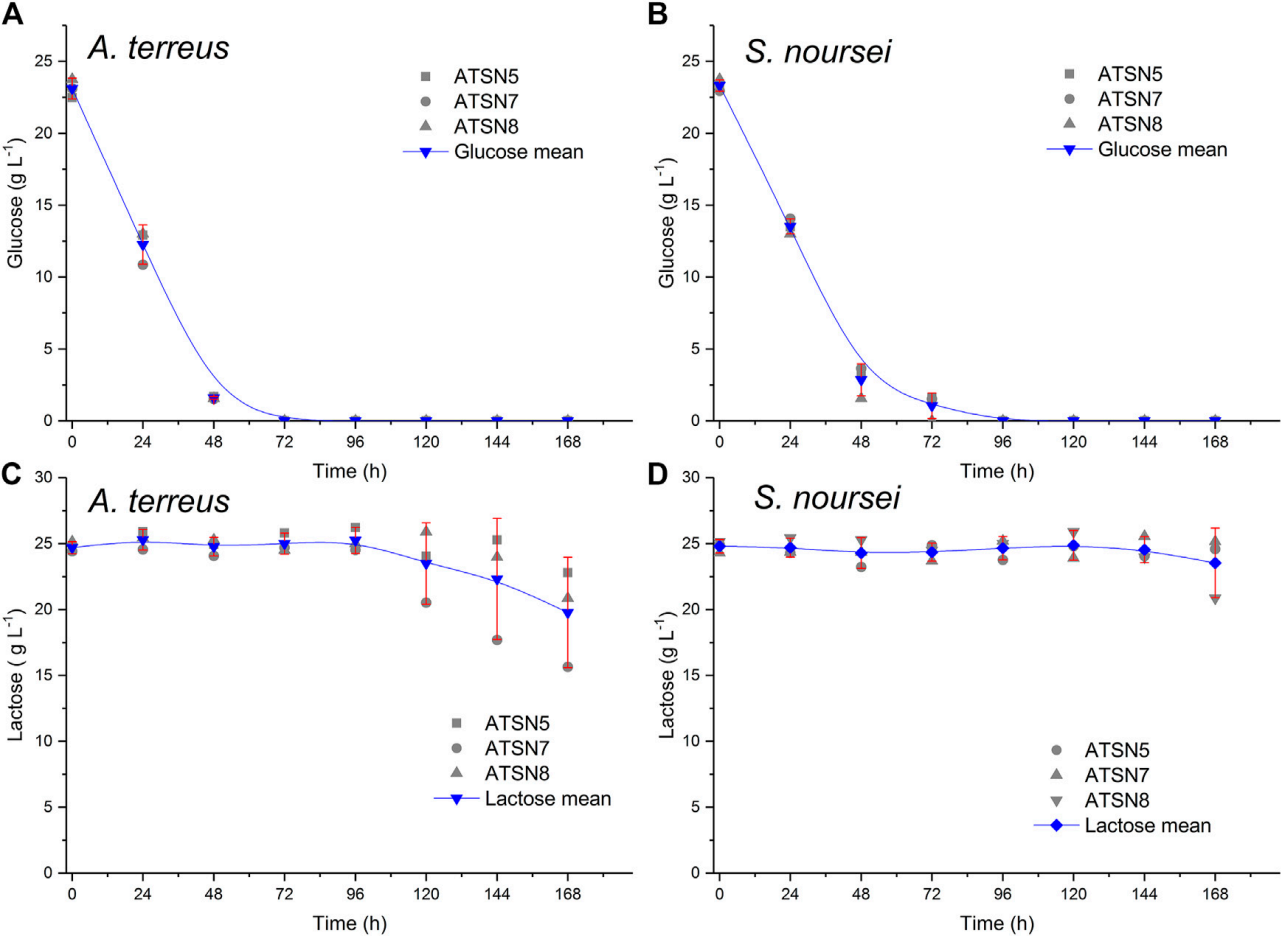
Replicability analysis of glucose **(A,B)** and lactose **(C,D)** concentration values determined over the course of the study for the *A. terreus* **(A,C)** and *S. noursei* **(B,D)** monocultures. The replicability was tested with the use of the confidence function in OriginPro 2017 software (OriginLab, United States) which returns the confidence interval for the mean value. Calculations were carried out for the significance level α = 0.05 based on three bioreactor experiments conducted in the same conditions, namely the monocultures in ATSN5, ATSN7 and ATSN8 runs.

**FIGURE 2:**
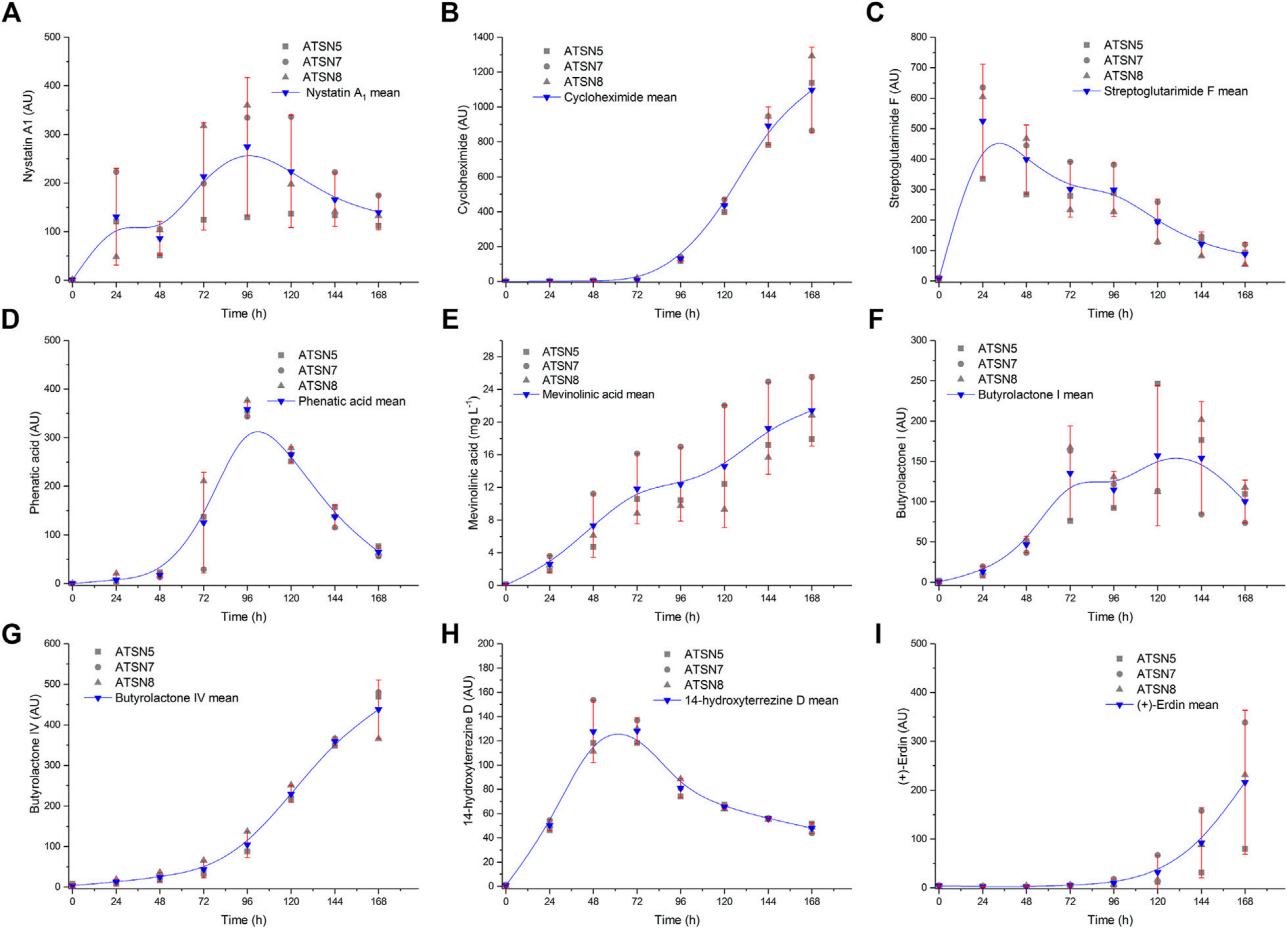
Replicability analysis of secondary metabolites levels determined over the course of the study for *S. noursei* **(A–D)** and *A. terreus* **(E–I)** monocultures. The replicability was tested with the use of the confidence function in OriginPro 2017 software (OriginLab, United States) which returns the confidence interval for the mean value. Calculations were carried out for the significance level α = 0.05 based on three bioreactor experiments conducted in the same conditions, namely the monocultures in ATSN5, ATSN seven and ATSN8 runs. AU—auxiliary units.

The assesment of experimental replicability was performed with regard to the levels of carbon substrates (Figure 1) and the selected secondary metabolites (Figure 2) based on three bioreactor monocultures conducted under equivalent conditions, namely ATSN5, ATSN7 and ATSN8. The replicability was tested with the use of the confidence function in OriginPro 2017 software (OriginLab, United States) which returns the confidence interval for the mean value. Calculations were carried out for the significance level *α* = 0.05.

With regard to the mass spectrometry data, the absolute error Δ(*m/z*) was calculated as follows:

$$\Delta \left(m/z\right)={\left(m/z\right)}_{\text{experimental}}-{\left(m/z\right)}_{\text{theoretical}}$$

where *z* stands for ion charge (*z* = 1) and *m* stands for monoisotopic mass. The (*m/z*)_theoretical_ value was calculated on the basis of tabularized physicochemical data. The experimental value of *m/z* for a given metabolite was constant throughout the study and showed no variation among the tested cultivation variants.

## 3 Results

In total, 50 previously discovered secondary metabolites were identified over the course of the present study (Table 2), including mevinolinic acid (β-hydroxy acid form of lovastatin) and nystatin A1. The identification process was mainly based upon the thorough analysis of mass spectra recorded over the course of mono- and co-cultivations and comparing the experimental *m/z* values with the theoretical ones presented in metabolic databases (Table 2). With regard to the *m/z* values of 280.1520, 310.1324, 292.1167, and 274.1042 (under ESI^−^ ionization), the comparison of *m/z* values proved to be insufficient to identify the molecules and the additional analysis of fragmentation patterns was performed (as described in the Supplementary Material). As far as the products represented by the *m/z* values of 449.2758, 477.2576, and 285.1204 (at ESI^−^) were concerned, it was not possible to distinguish between the biosynthetically related isomers by using mass spectrometry and these molecules were eventually referred to as “eurystatin A or D”, “terreustoxin A or B″ and “speradine B or G″, respectively (Table 2). After the identification of metabolites, their production profiles were analyzed by considering the peak areas corresponding to the respective *m/z* values. The production profiles of the secondary metabolites attributed to *S. noursei* (Supplementary Figure S1–S19) and *A. terreus* (Supplementary Figure S20–S50) are provided in the Supplementary Material of the current report. The complete catalogs of secondary metabolites were considered by examining the total ion chromatograms (TICs) obtained over the course of mono- and co-cultivations. By comparing the biosynthetic spectrum of the co-culture and the two corresponding monocultures one could evaluate if one of the co-cultured species dominated its partner (e.g., the similarity between the TICs corresponding to the co-culture and the *A. terreus* monoculture indicated that *A. terreus* was the dominant microorganism). The aligned TICs are presented in the Supplementary Material (see Supplementary Figure S51–S56).

**TABLE 2:** Secondary metabolites identified over the course of the study.

Retention time [min]	Ionization	(*m/z*)_experimental_	Assigned formula	(*m/z)*_theoretical_	Δ(*m/z*)	Assigned metabolite	Producing species	Effect of co-cultivation on metabolite production^a^						Literature
								ATSN3	ATSN4	ATSN5	ATSN6	ATSN7	ATSN8	
5.88	ESI^−^	924.4999	C_47_H_74_O_17_N	924.4957	+0.0042	nystatin A1	*S. noursei*	−	−	+/−	−	0	−	Borowski et al., 1971; Brautaset et al., 2000
5.92	ESI^−^	280.1520	C_15_H_22_O_4_N	280.1549	−0.0029	secocycloheximide	*S. noursei*	−	−	+/−	0	+/−	−	Huang et al., 2011; Morishita et al., 1998
5.28	ESI^−^	280.1520	C_15_H_22_O_4_N	280.1549	−0.0029	cycloheximide	*S. noursei*	−	−	0	0	+/−	−	
5.77	ESI^−^	280.1520	C_15_H_22_O_4_N	280.1549	−0.0029	A75943	*S. noursei*	−	−	0	0	+/−	−	
4.83	ESI^−^	310.1324	C_15_H_20_O_6_N	310.1291	+0.0033	streptoglutarimide F	*S. noursei*	−	−	0	+/−	+	−	Zhang et al. (2019)
5.34	ESI^−^	292.1167	C_15_H_18_O_1_N	291.1185	−0.0018	phenatic acid A	*S. noursei*	−	−	0	+/−	+	−	Fukuda et al. (2005)
6.31	ESI^−^	274.1042	C_15_H_16_O_4_N	274.1079	−0.0037	3-(2-hydroxy-3,5-dimethylphenacyl)glutarimide (actiphenol)	*S. noursei*	−	−	0	0	+	−	Yin et al. (2014)
6.30	ESI^−^	290.1028	C_15_H_16_O_5_N	290.1028	0.0000	3-[2-[2-hydroxy-3-methylphenyl-5-(hydroxymethyl)]-2-oxoethyl]glutarimide	*S. noursei*	−	−	0	+/−	+/−	−	Sun et al. (2014)
7.24	ESI^−^	163.0753	C_10_H_11_O_2_	163.0759	−0.0006	2-phenylethyl acetate	*S. noursei*	−	−	0	+/−	+/−	−	Rojas et al. (2001)
6.00	ESI^−^	449.2758	C_23_H_37_O_5_N_4_	449.2764	−0.0006	eurystatin A or D	*S. noursei*	−	−	0	−	+	−	Suzuki et al. (1994)
5.57	ESI^−^	435.2554	C_22_H_35_O_5_N_4_	435.2607	−0.0053	eurystatin C	*S. noursei*	−	−	0	−	+	−	
6.00	ESI^−^	276.1201	C_15_H_18_O_4_N_1_	276.1236	−0.0053	obscurolide B3	*S. noursei*	−	−	0	+	+/−	−	Hoff et al. (1992)
4.80	ESI^+^	264.1572	C_15_H_22_O_3_N_1_	264.1600	−0.0028	streptoprenylindole C	*S. noursei*	−	−	++	+/−	++	−	Yi et al. (2019)
4.86	ESI^+^	601.3576	C_27_H_49_O_9_N_6_	601.3561	+0.0015	desferrioxamine E	*S. noursei*	−	−	−	+++	−	−	Yamanaka et al. (2005)
4.80	ESI^+^	585.3549	C_27_H_49_O_8_N_6_	585.3612	−0.0063	deshydroxynocardamine	*S. noursei*	−	−	+	+++	+	−	Lee et al. (2005)
7.73	ESI^+^	379.2491	C_22_H_35_O_5_	379.2485	+0.0006	actinofuranone A	*S. noursei*	−	−	+++	+/−	−	−	Cho et al., 2006
7.20	ESI^+^	257.1300	C_15_H_17_O_2_N_2_	257.1290	+0.0010	albonoursin	*S. noursei*	−	−	+	0	++	−	Kansaki et al., 2000; Lautru et al., 2002
4.27	ESI^+^	238.1678	C_11_H_20_O_1_N_5_	238.1668	−0.0010	argvalin	*S. noursei*	−	−	+	0	+	−	Tatsuta et al. (1973)
6.47	ESI^+^	293.1290	C_18_H_17_O_2_N_2_	293.1290	0.0000	endophenazine A	*S. noursei*	−	−	+/−	+	+/−	−	Krastel et al. (2002)
8.62	ESI^−^	421.2598	C_24_H_37_O_6_	421.2590	+0.0008	mevinolinic acid (lovastatin acid)	*A. terreus*	++	0	−	0	−	0	Alberts et al. (1980)
9.34	ESI^−^	423.2722	C_24_H_39_O_6_	423.2747	−0.0025	4a,5-dihydromevinolinic acid	*A. terreus*	++	0	−	+/−	−	0	Albers-Schönberg et al. (1981)
6.56	ESI^−^	339.2166	C_19_H_31_O_5_	399.2172	−0.0006	3α-hydroxy-3,5-dihydromonacolin L	*A. terreus*	++	0	−	0	−	0	Nakamura et al. (1990)
8.08	ESI^−^	396.9890	C_17_H_11_O_7_Cl_2_	396.9882	+0.0008	(+)-geodin	*A. terreus*	+/−	0	−	−	−	0	Calam et al. (1947)
7.65	ESI^−^	382.9689	C_16_H_9_O_7_Cl_2_	382.9725	−0.0036	(+)-erdin	*A. terreus*	0	−	−	−	−	0	
7.44	ESI^−^	347.0801	C_17_H_15_O_8_	347.0767	+0.0034	asterric acid	*A. terreus*	0	0	−	−	−	0	Curtis et al. (1960)
8.38	ESI^−^	399.0037	C_17_H_13_O_7_Cl_2_	399.0038	−0.0001	(+)-geodin hydrate	*A. terreus*	+/−	0	−	−	−	0	Hassall and McMorris (1959)
7.99	ESI^−^	423.1462	C_24_H_23_O_7_	423.1444	+0.0018	butyrolactone I	*A. terreus*	−	++	−	+	−	+/−	Rao et al. (2000)
7.01	ESI^−^	439.1404	C_24_H_23_O_8_	439.1393	+0.0011	butyrolactone IV	*A. terreus*	+/−	+/−	−	0	−	+/−	
5.80	ESI^−^	236.0896	C_12_H_14_O_4_N	236.0923	−0.0027	isoflavipucine	*A. terreus*	0	0	−	−	−	0	Gressler et al. (2011)
5.60	ESI^−^	238.1072	C_12_H_16_O_4_N	238.1079	−0.0007	dihydroisoflavipucine	*A. terreus*	0	0	−	−	−	0	
3.16	ESI+	477.2576	C_26_H_37_O_8_	477.2488	+0.0088	terreustoxin A or B	*A. terreus*	++	0	−	0	−	+/−	Feng et al. (2019)
8.79	ESI^−^	291.1611	C_17_H_23_O_4_	291.1596	+0.0015	aspereusin D	*A. terreus*	0	−	−	−	−	−	Wang et al. (2018)
11.71	ESI^−^	295.0606	C_17_H_11_O_5_	295.0607	−0.0001	aspulvinone E	*A. terreus*	−	++	−	−	−	+	Ojima et al. (1973)
6.66	ESI^−^	324.1691	C_19_H_22_O_2_N_3_	324.1712	−0.0021	terezine D	*A. terreus*	+/−	++	−	+	−	+	Wang et al., 1995; Zhang et al., 2008
6.62	ESI^−^	340.1674	C_19_H_22_O_3_N_3_	340.1661	+0.0013	14-hydroxyterezine D	*A. terreus*	+/−	+	−	+/−	−	+	
4.80	ESI^−^	381.0975	C_21_H_17_O_7_	381.0974	+0.0001	mitorubrin	*A. terreus*	+/−	++	−	++	−	++	Büchi et al. (1965)
7.89	ESI^−^	356.1631	C_24_H_22_O_2_N	356.1651	−0.0020	protubonine A	*A. terreus*	+	+	−	++	−	0	Lee et al. (2011)
4.55	ESI^−^	335.0745	C_16_H_15_O_8_	335.0767	−0.0022	aspergillusone B	*A. terreus*	+/−	0	−	−	−	0	Nakashima et al. (2020)
5.93	ESI^−^	307.1524	C_17_H_23_O_5_	307.1546	−0.0022	(-)-12-acetoxy-1-deoxysydonic acid	*A. terreus*	0	−	−	−	−	−	Elsbaey et al., 2019; Li et al., 2015
7.49	ESI^−^	305.1406	C_17_H_21_O_5_	305.1389	+0.0017	7-deoxy-7,14-didehydro-12-acetoxysydonic acid	*A. terreus*	0	−	−	−	−	−	
10.18	ESI^−^	289.0389	C_13_H_9_O_6_N_2_	289.0461	−0.0072	fumisoquin C	*A. terreus*	−	−	−	−	−	+/−	Baccile et al. (2016)
6.11	ESI^−^	333.1487	C_17_H_21_O_5_N_2_	333.1450	+0.0037	N-methoxyseptorinol	*A. terreus*	+/−	+/−	−	+++	−	+	Devys et al. (1992)
5.62	ESI^−^	257.1273	C_15_H_17_O_2_N_2_	257.1290	−0.0017	1-(2′,6′-dimethylphenyl)-2-n-propyl-1,2-dihydropyridazine-3,6-dione	*A. terreus*	−	−	−	+++	−	−	Elissawy et al. (2019)
5.6	ESI^−^	319.1276	C_21_H_19_O_3_	319.1334	−0.0058	nigerapyrone B	*A. terreus*	−	−	−	++	−	−	Liu et al. (2011)
7.95	ESI^−^	461.2247	C_25_H_33_O_8_	461.2175	+0.0072	asnovolin G	*A. terreus*	++	−	−	−	−	−	Ishikawa et al. (2016)
5.04	ESI^−^	291.0492	C_14_H_11_O_7_	291.0505	−0.0013	aspergilol F	*A. terreus*	0	−	−	−	−	+/−	Wu et al. (2016)
9.69	ESI^−^	337.1022	C_20_H_17_O_5_	337.1076	−0.0054	4″-deoxy-3-hydroxyterphenyllin	*A. terreus*	+/−	0	−	+++	−	+/−	Guo et al. (2012)
5.66	ESI^−^	247.0285	C_9_H_11_O_6_S	247.0276	+0.0009	3-methoxy-6-methyl-5-(methylsulfonyl)benzene-1,2,4-triol	*A. terreus*	+/−	+++	−	+/−	−	+++	Yu et al. (2019)
7.35	ESI^−^	415.1357	C_23_H_19_O_4_N_4_	415.1406	−0.0049	isochaetominine B	*A. terreus*	−	+/−	−	−	−	+/−	Liao et al. (2015)
7.79	ESI^−^	285.1204	C_16_H_17_O_3_N_2_	285.1239	−0.0035	speradine B or G	*A. terreus*	−	−	−	+++	−	−	Hu et al. (2014)

a ”−”, no production; “0”, no enhancement; “+/−”, partial enhancement (not throughout the entire process); “+“, enhancement; “++“, strong enhancement (more than by 50% compared to monoculture); “+++“, very strong enhancement (more than twice compared to monoculture).

**FIGURE 3:**
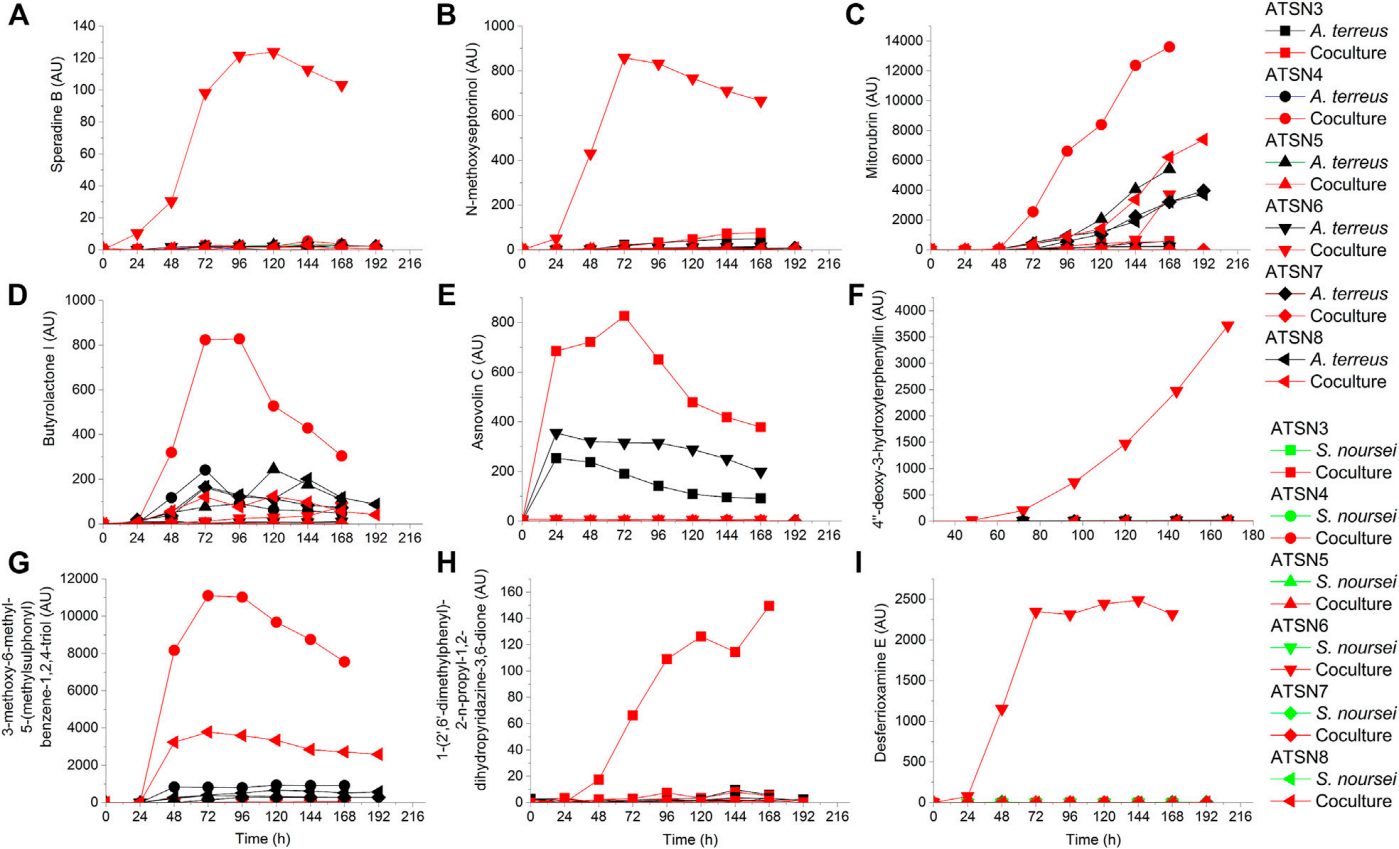
Production profiles of secondary metabolites that were markedly stimulated under the conditions of co-cultivation, namely **(A)** speradine B, **(B)** N-methoxyseptorinol, **(C)** mitorubrin, **(D)** butyrolactone I, **(E)** asnovolin C, **(F)** 4″-deoxy-3-hydroxyterphenyllin, **(G)** 3-methoxy-6-methyl-5-(methylsulphonyl)benzene-1,2,4-triol, **(H)** 1-(2′,6′-dimethylphenyl)-2-n-propyl-1,2-dihydropyridazine-3,6-dione, and **(I)** desferrioxamine **(E)**. AU—auxiliary units.

Compared to the results recorded for the monocultures, the co-cultivation approach led to the induction or marked stimulation of the biosynthesis of several secondary metabolites. Depending on the metabolite and the bioreactor run, the strength of the stimulatory effects differed considerably (as summarized in Table 2). The production profiles of selected metabolites that were strongly influenced by employing the co-cultivation approach are presented in Figure 3. The ATSN6 co-cultivation run was seen as particularly effective in the context of enhancing the biosynthetic capabilities of the investigated strains, as it visibly affected the production of molecules identified as speradine B or G (Figure 3A), N-methoxyseptorinol (Figure 3B), 4″-deoxy-3-hydroxyterphenyllin (Figure 3F) and desferrioxamine E (Figure 3I), the metabolites which were either absent or present only in trace amounts in the remaining experimental variants. The stimulatory effects were also recorded for the ATSN4 co-culture and involved the formation of 3-methoxy-6-methyl-5-(methylsulphonyl)benzene-1,2,4-triol (Figure 3G), butyrolactone I (Figure 3D) and mitorubrin (Figure 3C). In this context, the ATSN3 co-cultivation also led to notable outcomes, as it resulted in the visible enhancements in terms of asnovolin C (Figure 3E) and 1-(2′,6′-dimethylphenyl)-2-n-propyl-1,2-dihydropyridazine-3,6-dione (Figure 3H) levels.

**FIGURE 4:**
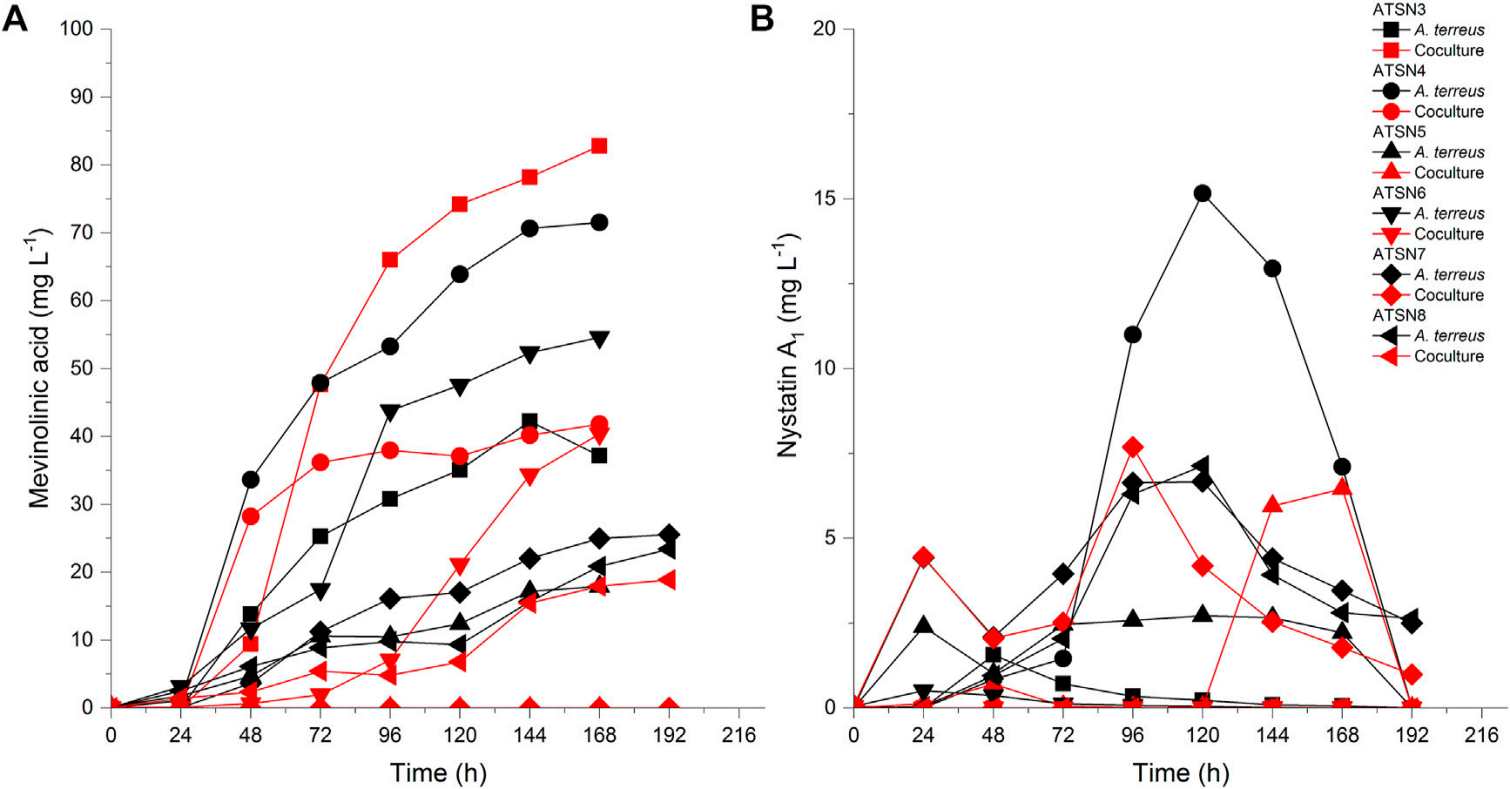
Production profiles of **(A)** mevinolinic acid and **(B)** nystatin A1 under the conditions of mono- and co-cultivation.

The presence of mevinolinic acid was confirmed in all tested variants except the ATSN7 co-culture. The highest titer of this metabolite (82.8 mg L^−1^) was recorded in the ATSN3 co-cultivation (Figure 4A). Among the six performed experiments, the ATSN3 co-culture was exceptional in terms of elevating the mevinolinic acid levels relative to the *A. terreus* monoculture. Similar observations concerning the stimulatory effects in ATSN3 were made with regard to the molecules biosynthetically related to mevinolinic acid, namely 4a,5-dihydromevinolinic acid (Supplementary Figure S21), and 3α-hydroxy-3,5-dihydromonacolin L (Supplementary Figure S22). In addition to the metabolites representing the mevinolinic acid pathway, *A. terreus* provided an array of products that were previously found in this species, namely (+)-geodin (Supplementary Figure S23), (+)-erdin (Supplementary Figure S24), asterric acid (Supplementary Figure S25), (+)-geodin hydrate (Supplementary Figure S26), butyrolactone I (Figure 3D), butyrolactone IV (Supplementary Figure S28), isoflavipucine (Supplementary Figure S29), dihydroflavipucine (Supplementary Figure S30), terreustoxin A or B (Supplementary Figure S31), aspereusin D (Supplementary Figure S32) and aspulvinone E (Supplementary Figure S33). Considering this group of molecules, the co-cultivation approach in ATSN4 was shown to be successful in terms of increasing the levels of butyrolactone I (Figure 3D) and aspulvinone E (Supplementary Figure S33), while the ATSN3 co-culture resulted in the increased levels of terreustoxin A or B (Supplementary Figure S31). The remaining metabolites assigned to *A. terreus* (Table 2), except for N-methoxyseptorinol, were previously demonstrated to be produced by various *Aspergilli*. This group of molecules encompassed, among others, terezine D (Supplementary Figure S34), mitorubrin (Figure 3C), protubonine A (Supplementary Figure S37), and aspergillusone B (Supplementary Figure S38). The influence of co-cultivation on the production of these secondary metabolites varied greatly depending on the compound and the bioreactor run (Table 2).

As far as the production of nystatin A1 by *S. noursei* was concerned, the highest titer of this metabolite (15.2 mg L^−1^) was reached in the ATSN4 monoculture (Figure 4B). Only in the ATSN5 run was the biosynthesis of nystatin A1 in the co-culture improved relative to the monoculture. However, this effect could be observed not earlier than 144 h after the inoculation (Figure 4B). Apart from nystatin A1, the study revealed several other *S. noursei* secondary metabolites that were previously identified as the products of *Streptomyces* (Table 2). The influence of co-cultivation on the formation of these molecules varied greatly and depended on the product and the bioreactor run. However, it was noticed that the ATSN4, ATSN5, and ATSN6 co-cultures were the only ones that showed positive effects in this regard. Above all, the strong enhancement of actinofuranone A (Supplementary Figure S16) and deshydroxynocardamine (Supplementary Figure S15) formation was observed in the ATSN5 and ATSN6 runs, respectively. The stimulatory influence of co-cultivation was also noted for argvalin (Supplementary Figure S18), albonoursin (Supplementary Figure S17), streptoglutarimide F (Supplementary Figure S5), phenatic acid (Supplementary Figure S6), eurystatin A or D (Supplementary Figure S10), actiphenol (Supplementary Figure S7), and obscurolide B3 (Supplementary Figure S12).

**FIGURE 5:**
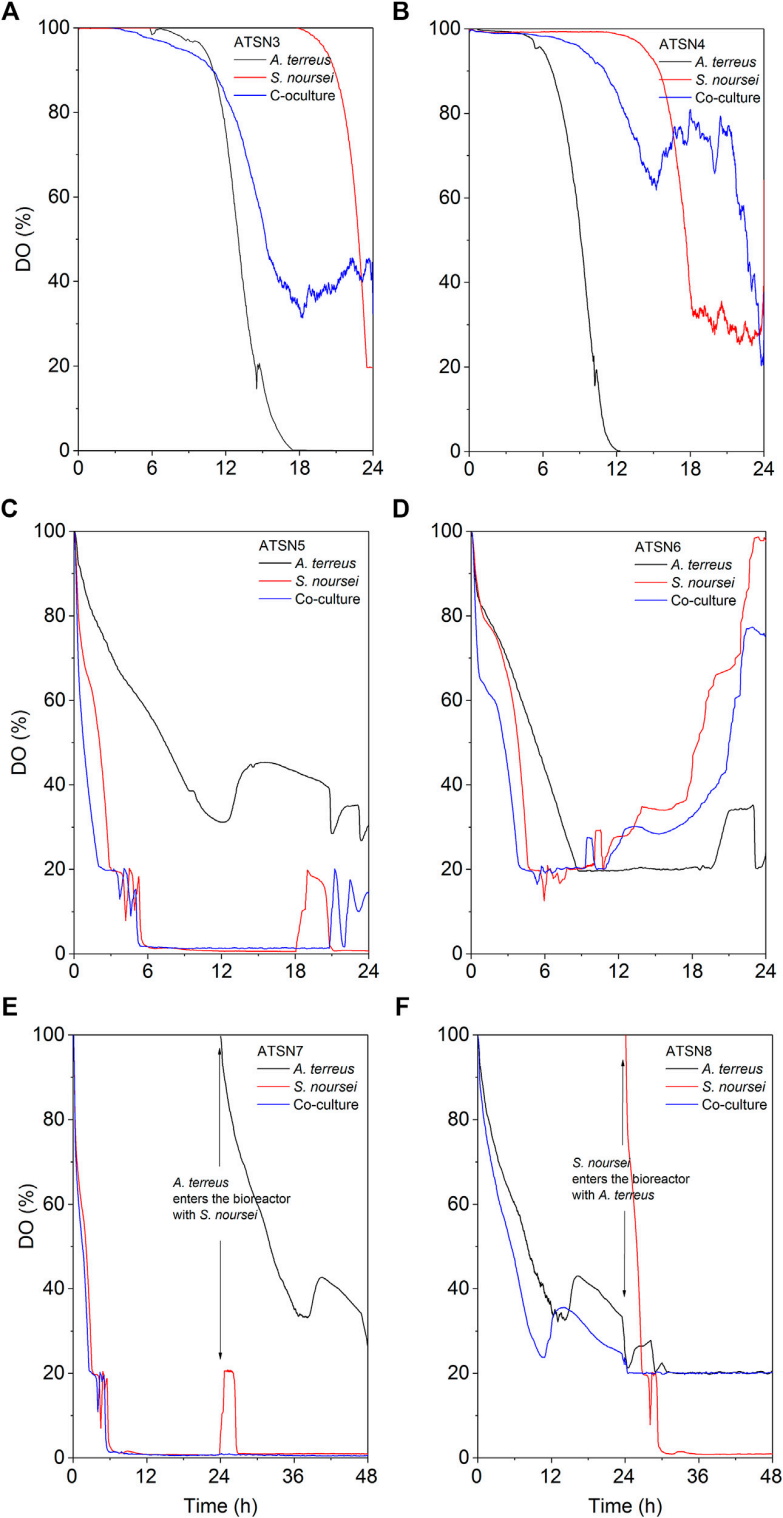
Changes of dissolved oxygen (DO) level in A. *terreus* and S. *noursei* co-cultures and the respective control monocultures in the **(A)** ATSN3, **(B)** ATSN4, **(C)** ATSN5, **(D)** ATSN6, **(E)** ATSN7, and **(F)** ATSN8 experiments during the first 24 h (48 h for ATSN7 and ATSN8) of cultivation.

**FIGURE 6:**
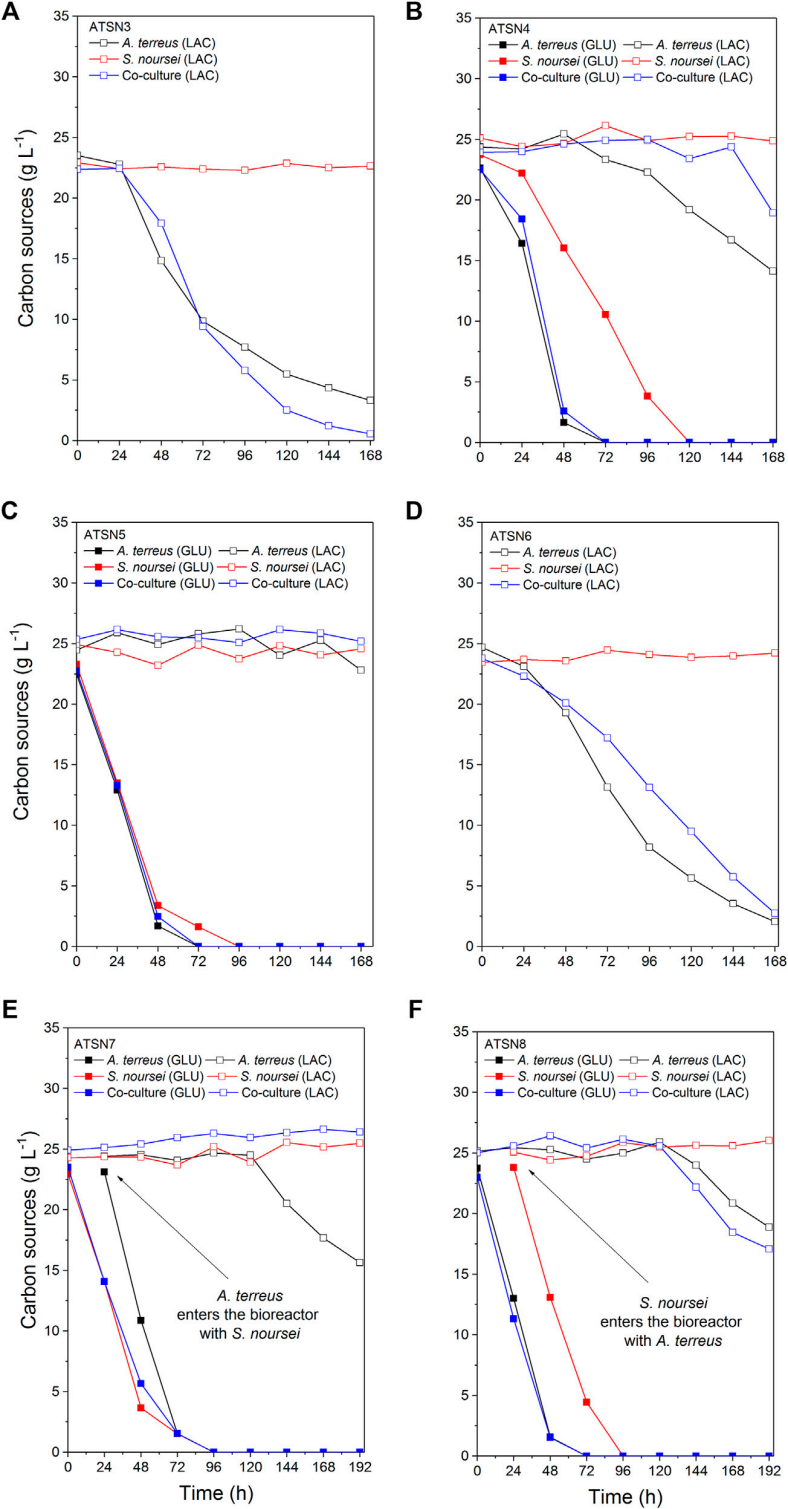
Temporal changes of glucose and lactose concentration in A. *terreus* and S. *noursei* co-cultures and the corresponding monocultures in the **(A)** ATSN3, **(B)** ATSN4, **(C)** ATSN5, **(D)** ATSN6, **(E)** ATSN7, and **(F)** ATSN8 experiments.

**FIGURE 7:**
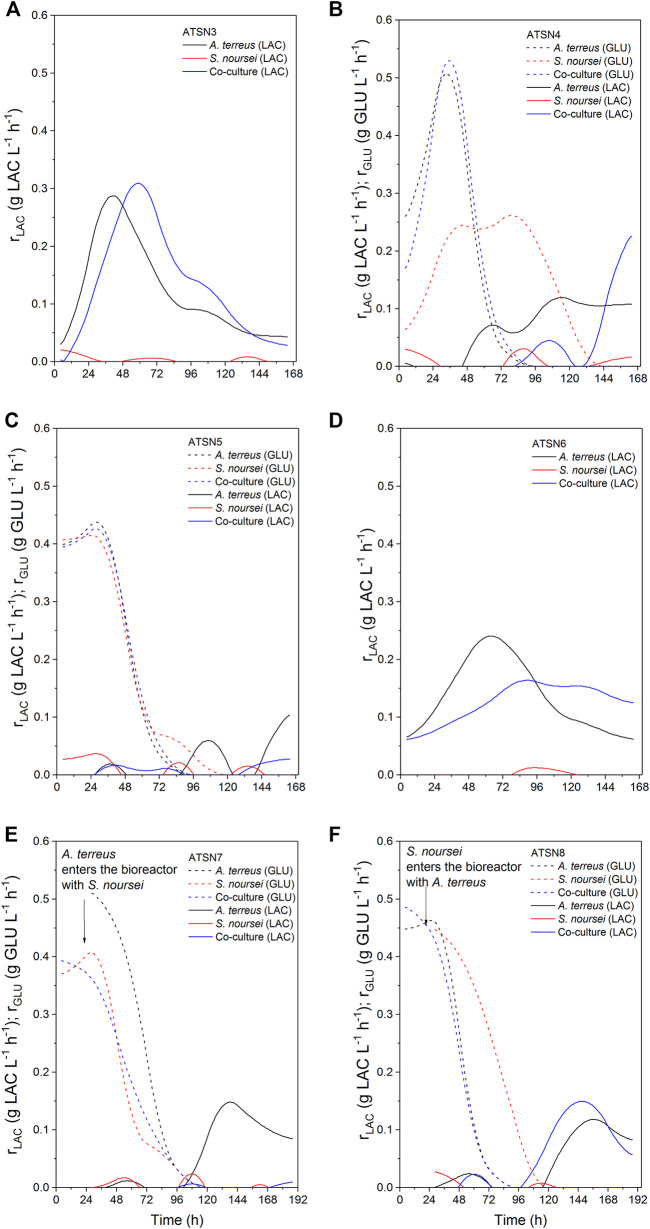
Kinetics of carbon substrates (glucose and lactose) uptake in A. *terreus* and S. *noursei* co-cultures and the corresponding monocultures in the **(A)** ATSN3, **(B)** ATSN4, **(C)** ATSN5, **(D)** ATSN6, **(E)** ATSN7, and **(F)** ATSN8 experiments.

The results concerning dissolved oxygen profiles (Figure 5) and carbon substrates utilization (Figures 6, 7) in “*A. terreus* vs *S. noursei*” co-cultures were monitored throughout the study. As far as the ATSN3 and ATSN4 runs were concerned, they were both initiated with the use of spores but differed with respect to the employed carbon substrates. Lactose was applied as the carbon source in ATSN3, whereas lactose and glucose were used in ATSN4. Even though *S. noursei* showed no capability to assimilate lactose in the ATSN3 monoculture (Figures 6A, 7A), the growth of *S. noursei* biomass was observed there. In ATSN3 co-culture the increase of lactose consumption rate was moved in time by about 12 h compared to *A. terreus* monoculture run, but both r_LAC_ curve shapes were practically identical and maximum volumetric lactose uptake rates were around 0.3 g LAC L^−1^ h^−1^ (Figure 7A). While *S. noursei* did not assimilate oxygen until 18 h of the ATSN3 run, it took *A. terreus* only 9 h to start oxygen consumption (Figure 5A). The DO profiles in the *A. terreus* monoculture and the co-culture were similar until 18 h, but then DO in *A. terreus* monoculture dropped to zero, whereas the co-culture displayed no decrease in its level (Figure 5A). The oxygen consumption in the ATSN4 run started around 6 h earlier than in the ATSN3 experiment and this was observed both for *A. terreus* and *S. noursei* (Figure 5B). As long as glucose was present in the ATSN4 medium, hardly any lactose utilization was recorded in the co-culture and *A. terreus* monoculture. Furthermore, *S. noursei* showed no lactose utilization in the ATSN4 monoculture, what was in agreement with the previous observations made during the ATSN3 experiment (Figure 6B). It was also noticed that the profiles of glucose concentration and r_GLU_ recorded for the ATSN4 co-culture very much resembled the ones observed for the corresponding *A. terreus* monoculture (Figure 7B).

The subsequent two runs, namely ATSN5 and ATSN6, were initiated with the use of 24-h precultures instead of spores. In ATSN5, the time courses of glucose concentration and r_GLU_ were highly similar among all three variants, i.e., the co-culture and the monocultures of *A. terreus* and *S. noursei* (Figure 7C). The consumption of lactose was observed solely in the *A. terreus* monoculture but not before 100 h of the run. As far as the DO profiles were concerned, there was a striking similarity between the data collected for the monoculture of *S. noursei* and the co-culture (Figure 5C). In ATSN6 it was noticed that the maximum lactose uptake rate was even 1.6-fold lower (r_LAC_ = 0.15 g LAC L^−1^ h^−1^) in the co-culture than in the *A. terreus* monoculture (r_LAC_ = 0.25 g LAC L^−1^ h^−1^). What is more, the r_LAC_ values recorded for the ATSN6 co-culture were lower than the ones observed for the corresponding *A. terreus* monoculture until 96 h of the run (Figure 7D).

In the subsequent experiment, namely ATSN7, the co-cultivation was initiated by transferring the 24-h flask preculture of *A. terreus* into the already developed 24-h bioreactor culture of *S. noursei*. In this variant, the profiles of glucose concentration (Figure 6E), glucose uptake rates (Figure 7E), and dissolved oxygen (Figure 5E) were observed to be practically identical in the co-culture and the *S. noursei* monoculture. Furthermore, no lactose utilization was recorded in the co-culture, whereas in the *A. terreus* monoculture lactose was assimilated after glucose depletion, i.e., starting from 96 h of the run, and reached its peak value (about 0.15 g LAC L^−1^ h^−1^) at 144 h (Figure 7E).

In the last experiment conducted within the present study, designated ATSN8, the co-culture was started by introducing the flask preculture of *S. noursei* into the bioreactor culture of *A. terreus*. This time, not only was lactose visibly assimilated in the co-culture, but the profiles of lactose concentration and lactose uptake rates in the co-culture and *A. terreus* monoculture were strikingly similar (Figures 6F, 7F). In both variants, lactose utilization started between 96 and 120 h of the run, which was the time corresponding to the depletion of glucose in the broth (Figure 6F). The similarity between the co-culture and *A. terreus* monoculture was also noticed with respect to glucose concentration and uptake rate profiles (Figures 6F, 7F) as well as the dissolved oxygen levels (Figure 5F).

**FIGURE 8:**
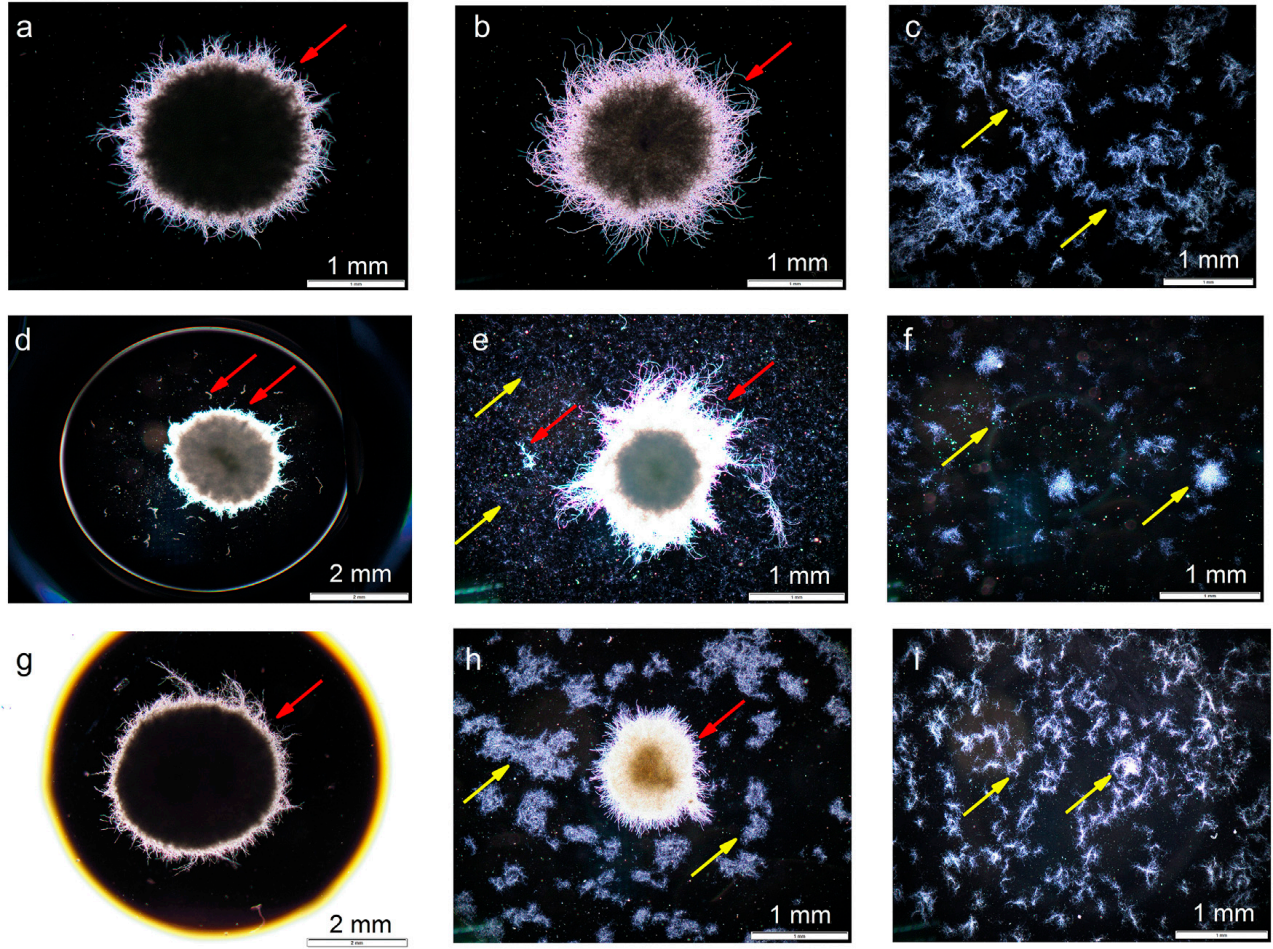
Microscopic images corresponding to **(A)** ATSN4 monoculture of *A. terreus*, **(B)** ATSN4 co-culture, **(C)** ATSN4 monoculture of *S. noursei*, **(D)** ATSN6 monoculture of *A. terreus*, **(E)** ATSN6 co-culture, **(F)** ATSN6 monoculture of *S. noursei*, **(G)** ATSN7 monoculture of *A. terreus*, **(H)** ATSN7 co-culture, and **(I)** ATSN7 monoculture of *S. noursei*. All images were taken at 48 h of the cultivation process. Red and yellow lines depict *A. terreus* and *S. noursei*, respectively.

The experiments were complemented with the microscopic observations of filamentous structures. The selected examples of images are presented in Figure 8. Mycelial pellets and clumps were the predominant forms exhibited by *A. terreus* (Figures 8A,B, D,E, G,H) and *S. noursei* (Figures 8C,E,F,H,I), respectively.

## 4 Discussion

In the present study, the bioreactor co-cultures *A. terreus* and *S. noursei* were characterized in the context of secondary metabolites production and bioprocess kinetics. The main intention behind this work was to provide a comprehensive view of the biosynthetic capabilities exhibited in the co-cultures and compare them with the results recorded for the corresponding monocultures. In total, a rich catalog of 50 secondary metabolites was analyzed over the course of the study. A major part of the work focused on the identification of these metabolic products based on mass spectra corresponding to the tested culture variants. Another broad dataset included the time courses of dissolved oxygen (DO) concentration, the levels of glucose and lactose, and their volumetric uptake rates.

Among the 50 identified secondary metabolites, 19 and 31 molecules were found to be produced by *S. noursei* and *A. terreus*, respectively (Table 2). Assigning the metabolites to their producers was mostly performed through the careful comparative analysis of monocultures. Some of these products had already been isolated from *S. noursei* or *A. terreus* and characterized prior to this study. For example, *S. noursei* was shown to be the source of nystatin A1, albonoursin as well as the biosynthetically related molecules known as cycloheximide, actiphenol, and phenatic acid (Brautaset et al., 2000; Lautru et al., 2002; Yin et al., 2014). The catalog of previously characterized *A. terreus* metabolites includes mevinolinic acid, 3α-hydroxy-3,5-dihydromonacolin L, 4a,5-dihydromevinolinic acid, (+)-geodin, asterric acid, (+)-erdin, (+)-geodin hydrate, butyrolactone I and IV, isoflavipucine, dihydroisoflavipucine, aspulvinone E and the terreustoxins (Calam et al., 1947; Curtis et al., 1960; Ojima et al., 1973; Alberts et al., 1980; Albers-Schönberg et al., 1981; Nakamura et al., 1990; Rao et al., 2000; Gressler et al., 2011; Feng et al., 2019). In addition to these products, the list of secondary metabolites identified throughout this study included many other molecules, which have not been yet isolated from *S. noursei* or *A. terreus* (Table 2).

As the spores practically do not utilize oxygen until the emergence of germ tubes, the DO profiles can be used as the indicators of spores germination (Yanagita, 1957). In the ATSN3 run, in which lactose was employed as a carbon source, the onset of oxygen utilization by *S. noursei* and *A. terreus* was observed at 18 and 9 h of the run, respectively (Figure 5A). In other words, the spores of *A. terreus* started to germinate relatively early compared to *S. noursei*. This behavior was also recorded in the ATSN4 experiment, in which both lactose and glucose were present in the medium (Figure 5B). Hence, having only lactose (as in ATSN3) or both lactose and glucose (as in ATSN4) in the medium was not the factor that defined the relative time of germination of the two strains.

Under the conditions applied throughout the study, there were no signs of lactose assimilation by *S. noursei*. A different behavior was previously recorded for *S. rimosus*, which is known to be capable of lactose utilization under similar bioprocess conditions (Boruta et al., 2021). It should be mentioned, however, that the incapability of lactose consumption did not prevent the visible growth of *S. noursei* in those ATSN monocultures in which there was no glucose added to the medium and the components of yeast extract must have been used as the sources of carbon for biomass development. In other words, the presence of *S. noursei* was manifested in the co-cultures even in the absence of glucose. It should be noted that according to the information provided by the manufacturer of yeast extract used in the study (BD Bionutrients Technical Manual, BD, United States) this product contains not only a rich mixture of amino acids but also a considerable amount of carbohydrates in its composition (0.13 g g^−1^) that potentially could be used as a source of carbon. In the ATSN3 experiment the DO profiles of the co-culture and the *A. terreus* monoculture resembled one another only up to 18 h of the run, the time which corresponded to the germination of *S. noursei* (Figure 5A). After this time, the DO level continued to decrease in the *A. terreus* monoculture but not in the co-culture. Hence, the presence of *S. noursei* exerted an inhibitory effect on the development of *A. terreus*, as reflected by the inhibition of oxygen consumption in the co-culture compared to the *A. terreus* monoculture (Figure 5A). However, this effect proved to be stronger in the ATSN4 run, in which glucose was used as a medium component. What is more, the presence of glucose in ATSN4 resulted in the earlier germination of *A. terreus* and *S. noursei* compared with ATSN3, as indicated by the times corresponding to the onset of oxygen consumption for the monocultures (Figure 5B). It is also worth mentioning that in the ATSN6 run, which did not involve the use of glucose, the maximum value of volumetric lactose uptake rate in the ATSN6 co-culture was considerably lower than that in the corresponding *A. terreus* monoculture (Figure 7D). This reflected the inhibitory effects exerted by *S. noursei* on *A. terreus* in the co-culture. Altogether, the study exemplified the strategy that can be used to monitor the activity of strains in the co-cultures, namely using the substrates that are metabolized solely by one of the co-cultured microorganisms. In this context, investigating substrate assimilation is a complementary method that can be employed in concert with the TICs and mass spectra analyses. Here, lactose utilization in the co-culture reflected the growth of *A. terreus*, as *S. noursei* displayed no capability to use this sugar. The strategy based on lactose level monitoring could not be used in the case of “*A. terreus* vs *S. rimosus*” co-cultures since *A. terreus* and *S. rimosus* are both known to metabolize lactose (Boruta et al., 2021).

One of the key issues to be considered when planning the co-cultivation experiments is the domination of a more “aggressive” strain over its partner. This effect is mostly associated with the relative growth rates of the co-cultured microorganisms and their capabilities to biosynthesize metabolites exhibiting antimicrobial activity. Obtaining a co-culture showing hardly any difference in terms of product formation, substrate consumption and morphological characteristics in comparison with one of the corresponding monocultures is not a surprising event, especially when conducting the preliminary runs. This phenomenon was very often observed in our previous works related to submerged co-cultivation of filamentous strains (Boruta et al., 2019, 2020, 2021; Boruta and Ścigaczewska, 2021). The domination can be assessed by considering the biosynthetic outcomes of the co-culture, as reflected by the total ion chromatograms (TICs) obtained by using liquid chromatography coupled with mass spectrometry, the time courses of substrates concentration and their uptake rates, as well as the dissolved oxygen levels. The more the profile recorded for the co-culture resembles the one obtained for the monoculture, the more similar are these two variants in terms of bioprocess outcomes. This comparative approach was applied in our previous study on the co-cultivation of *S. rimosus* and *A. terreus* (Boruta et al., 2021). It was demonstrated that the *S. rimosus* dominated *A. terreus* unless the latter had been given a 24-h growth advantage in the bioreactor. Importantly, this phenomenon was observed regardless of the medium composition (Boruta et al., 2021). In the present work, replacing *S. rimosus* with *S. noursei* as a co-culture partner of *A. terreus* had profound effects on the outcomes of co-cultivation, namely the fungus could no longer be perceived as a likely loser in the “actinomycete vs fungus” confrontation. In fact, when the co-culture was initiated with the use of spores, as in the ATSN3 and ATSN4 runs, the “victory” of *A. terreus* over *S. noursei* was observed. The similarity between the TICs recorded for the co-culture and the monoculture of *A. terreus* was evident in these cases (Supplementary Figure S51 and S52). The similarity of glucose level and glucose uptake rate profiles between the two variants was also noticeable in ATSN3 and ATSN4 (Figures 7A,B). Moreover, the consumption of lactose in the ATSN4 co-culture indicated that *A. terreus* was active there because *S. noursei* did not display any ability to assimilate lactose. Interesting observations were also made with regard to the ATSN5 and ATSN6 co-cultures, which were initiated by applying the 24-h precultures. In these two experiments, the process outcomes were dependent on the medium composition. In the ATSN5 run, which involved the medium containing lactose and glucose, the domination of *S. noursei* over *A. terreus* was evident. In this case, the *S. noursei* monoculture and the co-culture displayed visible similarities in terms of the TICs (Supplementary Figure S53) and dissolved oxygen profiles (Figure 5C). What is more, there was no lactose utilization in the ATSN5 co-culture (Figure 6C), what indicated that *A. terreus* was either eliminated or greatly inhibited compared with the monoculture. Interestingly, the domination of *S. noursei* was no longer seen in the ATSN6 run, in which glucose was eliminated from the list of substrates. The ATSN6 run was unique in the sense that no organism could be unequivocally indicated as being a “winner” of the clash. Despite the similarity in terms of lactose uptake rates (Figure 7D) and concentration profiles (Figure 6D) noted between the co-culture and *A. terreus* monoculture, after consulting the TICs (Supplementary Figure S54) it become clear that the outcome of the ATSN6 should not be perceived as a one-sided “victory” but rather a “draw”. None of the monoculture profiles represented by the TICs aligned well with the dataset recorded for the co-culture (Supplementary Figure S54), what led to a conclusion that both organisms visibly participated in the final biosynthetic outcome of the ATSN6 co-cultivation process. Considering the typical “win or lose” scenarios that were previously observed in the “*A. terreus* vs *S. rimosus*” study (Boruta et al., 2021) and in our remaining works on submerged co-cultivation, seeing the “draw” in the ATSN6 clash between *A. terreus* and *S. noursei* could be regarded as rather surprising. For example, in the study on the co-cultivation of *A. terreus* with the fungus *Penicillium rubens*, the co-existence of two strains that would generate observable levels of secondary metabolites required a non-standard inoculation procedure based on preculture volume adjustments (Boruta et al., 2020). Other works also demonstrated the importance of designing a specific inoculation scheme to prevent the overgrowth of the producing strain (Ezaki et al., 1992; Slattery et al., 2001; Luti and Mavituna, 2011; Carlson et al., 2015; Shin et al., 2018; Liang et al., 2020). In the present effort, achieving the simultaneous biosynthetic activity of two strains in the co-culture occurred by chance, without any targeted efforts to achieve the “draw” effect. Apparently, the differences with regard to growth rate and the capabilities to biosynthesize antimicrobial molecules were not as strong for the „*A. terreus* vs *S. noursei*” pair as they were for the previously examined “*A. terreus* vs *S. rimosus*” system (Boruta et al., 2021). Whereas the domination of *S. rimosus* over *A. terreus* could be associated with the biosynthesis of antifungal metabolites known as rimocidins (Boruta et al., 2021), it was not possible to unequivocally indicate the “chemical weapons” that *S. noursei* could have used to establish its potential domination over the fungal rival. Two aspects were important in terms of reaching the concerted development and the biosynthetic activity of the two strains in the ATSN6 experiment, namely the inoculation scheme and medium composition. Concerning the inoculation approach, using the 24-h precultures instead of spores meant that the burden of spore germination and initial biomass proliferation in the bioreactor were avoided. In other words, there was a portion of already developed biomass introduced into the bioreactor, which was less likely to be kept inactive than the spores in their pre-germination phase. Regarding the medium composition, it should be realized that the ATSN5 run was, just like ATSN6, based on the “preculture vs preculture” inoculation strategy, but in contrast to ATSN6 it led to a “victory” of *S. noursei* over *A. terreus* rather than the “draw”. This was due to the presence of glucose in the ATSN5 medium and the lack thereof in the ATSN6 process. Whereas the presence of glucose favored the development of *S. noursei* in the ATSN5 co-culture, the absence of this sugar in the ATSN6 medium resulted in equalizing the survival chances for the co-cultivated *S. noursei* and *A. terreus*. Most importantly, compared with other ATSN co-cultures, the ATSN6 “draw” was particularly effective in terms of stimulating the production of secondary metabolites (Figure 3), including the ones identified as the products of *A. terreus* (speradine B or G, N-methoxyseptorinol, and 4″-deoxy-3-hydroxyterphenyllin), as well as of *S. noursei* (desferrioxamine E). Hence, establishing a co-culture system that is not dominated by any of the strains can be viewed as a promising approach of stimulating the biosynthetic pathways.

The ATSN7 and ATSN8 co-cultures were based on the concept of providing a 24-h growth advantage to *S. noursei* or *A. terreus*, respectively. In these experiments, the organism that was granted the advantage eventually dominated the co-culture. This was in agreement with the observation made previously for *A. terreus* and *S. rimosus*, i.e., the strain introduced to the bioreactor 24 h before its competitor always turned out to be the “winner” of the clash (Boruta et al., 2021). It should also be mentioned that the difference between ATSN7 and ATSN8 runs was strictly associated with the inoculation scenario, whereas the initial composition of the growth medium was the same and included glucose and lactose as carbon sources. Therefore, there was no medium-related advantage provided to any of the participating strains.

The discussion regarding the “winners” and “losers” of submerged co-cultivation can also be conducted from the morphological perspective. The images that were presented in Figure 8 correspond to the experiments that represent different outcomes of the “*A. terreus* vs *S. noursei*” confrontation, namely ATSN4 (Figures 8A–C), ATSN6 (Figures 8D–F), and ATSN7 (Figures 8G–I). In the ATSN4 co-culture, which was dominated by *A. terreus*, the growth of *S. noursei* was practically non-observable (Figure 8B). In the ATSN6 co-cultivation, which resulted in the “draw”, the growth of both microorganisms was evident (Figure 8E). Finally, in the ATSN7 co-culture dominated by *S. noursei*, the pellets of *A. terreus* seemed underdeveloped compared to the ones seen in the *A. terreus* monoculture, i.e., they reached smaller sizes and less densely packed core regions (compare Figures 8G,H). Hence, the aforementioned findings referring to the biosynthetic activity and bioprocess behavior of “*A. terreus* vs *S. noursei*” co-cultures align well with the morphological observations.

*S. noursei* is widely known as a producer of nystatin A1, an effective antifungal substance. Enhancing the secretion of antifungals in response to the presence of a fungus in co-culture may be regarded as a way of gaining an advantage in the competitive environment. However, such a scenario was not confirmed in the present study and the highest titers of nystatin A1 were recorded for the ATSN4 monoculture (Figure 4B). Hence, the interactions of *S. noursei* with *A. terreus* in the co-culture failed to result in a marked stimulation of nystatin A1 biosynthesis compared with the levels noted for the monocultures (Figure 4B). On the other hand, the co-cultivation approach led to the improved biosynthesis of mevinolinic acid (β-hydroxy acid form of lovastatin) by *A. terreus*. This effect was visible in the ATSN3 co-culture, which was initiated with the use of spores and involved the use of medium containing lactose (but not glucose) as the carbon source. The effectiveness of lactose-containing medium in terms of elevating mevinolinic acid production stood in agreement with previous findings (reviewed by Mulder et al., 2015). It should be noted, however, that the co-culture-related stimulatory effect on mevinolinic acid production was not a universally observed phenomenon but rather an exceptional situation, i.e., it took place only in one of the investigated “mono-vs co-cultivation” scenarios, namely ATSN 3 (Figure 4A).

## 5 Conclusion

To conclude, this study provided the first description of the co-cultures involving *A. terreus* and *S. noursei*, two industrially relevant microorganisms. Instead of focusing on a limited number of key products and restraining to small-volume cultivations, this effort was designed as a comprehensive analysis of biosynthetic repertoires and bioprocess kinetics at the bioreactor scale. Most importantly, the submerged co-cultures of *A. terreus* and *S. noursei* were shown to be effective platforms for stimulating the biosynthesis of secondary metabolites in stirred tank bioreactors. The demonstrated possibility to achieve the concurrent development of both species in the co-culture opens the door for various future studies on microbial interactions. In this context, the present work revealed important differences between the “*A. terreus* vs *S. noursei*” system and the previously characterized “*A. terreus* vs *S. rimosus*” co-cultivation (Boruta et al., 2021). Importantly, the study highlighted the methodological approach of using the substrates that are selectively metabolized by the individual species to assess the activity of co-culture members. Even though the study did not show the enhancement of nystatin A1 biosynthesis in the bacterial-fungal co-culture, the results of the work indicated that bioreactor co-cultivation is a promising approach of stimulating the production of lovastatin and other bioactive metabolites originating from filamentous microorganisms.

## Data Availability

The original contributions presented in the study are included in the article/Supplementary Material, further inquiries can be directed to the corresponding authors.
