# Supplementary material for: Production of secondary metabolites in stirred tank bioreactor co-cultures of Streptomyces noursei and Aspergillus terreus
Source: Front Bioeng Biotechnol. 2022 Sep 29;10:1011220. doi: 10.3389/fbioe.2022.1011220 (PMC9557299; doi:10.3389/fbioe.2022.1011220)
Supplement: Supplementary file 1 [file DataSheet1.PDF]

Supplementary Material

Identification of the product at *m/z*=280.1520 (ESI$^-$)

A molecule of experimental monoisotopic mass at negative ionisation (ESI$^-$) equal to 280.1520 has its elemental composition of $[M-H]^-$ $C_{15}H_{22}O_4N$, which corresponded to the theoretical monoisotopic mass of 280.1549. There are four potential *Streptomyces* sp. metabolites that fit to *m/z*=280.1520 [Δ(*m/z*)=+0.0029]. These are: 210A, secocycloheximide, cycloheximide and A75943.

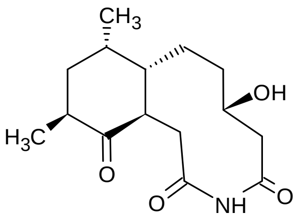

210A

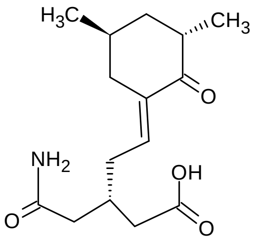

secocycloheximide

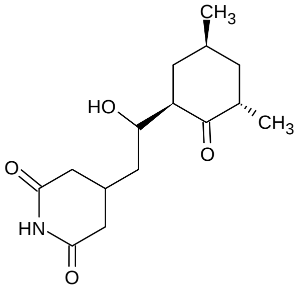

cycloheximide

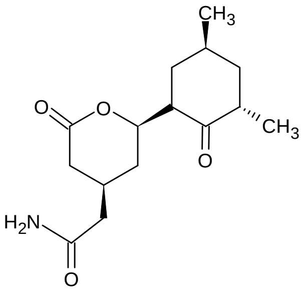

A75943

The molecules of *m/z*=280.1520 were eluted at three various retention times: t_{R1}=5.28 min, t_{R2}=5.77 min and t_{R3}=5.92 min and therefore they were not the same. The analysis of fragmentation was made with the use of ACD/MS Fragmenter software. Upon characteristic fragmentation pattern it was recognized that at t_{R1}=5.28 min. cycloheximide was eluted:

1. It formed a fragment $C_7H_8O_3N$ of *m/z*=154.0534 [Δ(*m/z*)=+0.0025]. Nevertheless, this fragment was also observed at t_{R2}=5.77 min. Out of these four metabolites only cycloheximide and A75943 could generate this fragment.
2. Only cycloheximide could accept water molecule due to opening the ring with the secondary nitrogen atom to form an adduct molecule of *m/z*=298.1627 [Δ(*m/z*)=+0.0027]. That is why only cycloheximide forms another fragment of *m/z*=172.0614, [Δ(*m/z*)=−0.0004], which was the fragment $C_7H_8O_3N$ (mentioned in point 1) with water attached $C_7H_8O_3N+H_2O$.

It was recognized that at t_{R2}=5.77 min A75943 was eluted. Its presence is revealed by the fragment $C_7H_8O_3N$ of *m/z*=154.0534 [Δ(*m/z*)=+0.0025] and it could not be cycloheximide as it was found above.

At t_{R3}=5.92 min either secocycloheximide or 210A were eluted. The detailed analysis revealed the presence of the characteristic fragment that could be only formed by cycloheximide $C_{14}H_{22}O_2N$ being the parent molecule with CHO_2 removed. Its monoisotopic experimental mass is 236.1635 [Δ(*m/z*)=+0.0016]. Therefore only secocycloheximide was eluted here.

Identification of the product at *m/z*=310.1324 (ESI$^-$)

At t_R=4.83 min (negative ionisation) two characteristic masses were found *m/z*=310.1324 [Δ(*m/z*)=−0.0033] corresponding to the formula $[M-H]^-$: $C_{15}H_{20}O_6N$ and *m/z*=621.2701 [Δ(*m/z*)=−0.0042], whose mass was twice as high the basic molecule. Carbon ^{13}C pattern revealed that the latter was just a dimer of $C_{15}H_{20}O_6N$ as the following masses were found:

Dimers of streptoglutarimide F:

Number of ^{13}C in the molecule	Experimental *m/z* of a molecule	Experimental *m/z* of a dimer	Number of ^{13}C in the dimer
0	310.1324	621.2701	0
1	311.1341	622.2731	2
		623.2768	3
2	312.1451	624.2814	4
		625.2867	5
3	313.1500	626.2928	6
		627.2998	7
4	314.1566	628.2966	8

Therefore *m/z*=310.1324 [Δ(*m/z*)=−0.0033] was under consideration. Two *Streptomyces* metabolites could be attributed to this mass:

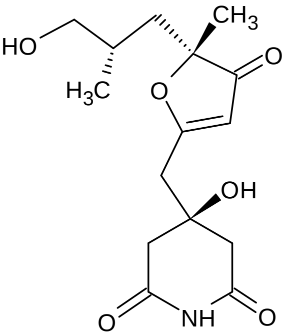

Streptoglutarimide E

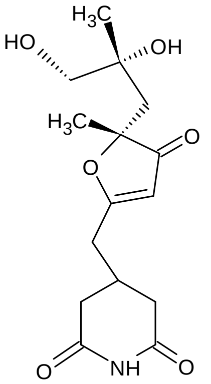

Streptoglutarimide F

The analysis of fragmentation was made with the use of ACD/MS Fragmenter software. Three characteristic fragments were found only for streptoglutarimide F ($[M-H]^-$formula: $C_{15}H_{20}O_6N$) only and they fitted to the experimental fragments. These were:

1. $C_{15}H_{20}O_5N$ (OH lost) of *m/z*=294.1302 [Δ(*m/z*)=+0.0038]
2. $C_{14}H_{20}O_4N$ (CH_2NO lost) of *m/z*=266.1431 [Δ(*m/z*)=−0.0069]
3. $C_{11}H_{12}O_4N$ ($C_4H_8O_2$ lost) of *m/z*=222.0765 [Δ(*m/z*)=+0.0005]

Streptoglutarimide F was also detected at t_R=4.83 min at ESI$^+$ as the ion of *m/z*=312.1459 [Δ(*m/z*)=−0.0012].

Identification of the product at *m/z*=292.1167 (ESI$^-$)

At t_{R1}=5.34 min and t_{R2}=6.31 min (negative ionisation) the experimental mass 292.1167 [Δ(*m/z*)=+0.0018] was found and various retention times indicated on two various metabolites of the same molecular weight. There were three candidates produced by *Streptomyces* sp.:

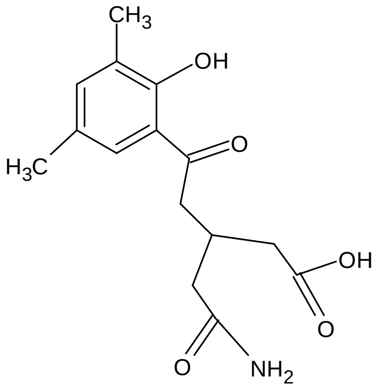

Phenatic acid A

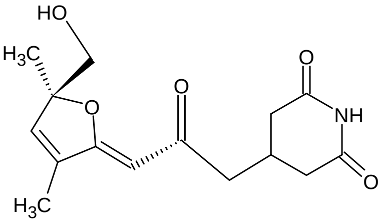

Streptoglutarimide C

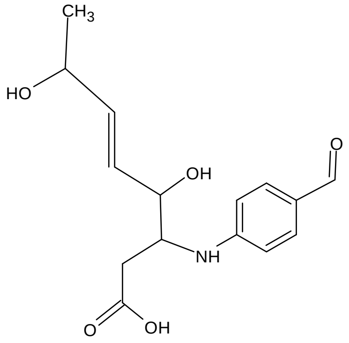

Obscurolide C2

Theis *m/z* corresponded to [M−H]$^-$formula: $C_{15}H_{18}O_5N$.
The analysis of M-H fragmentation at t_{R1}=5.34 min revealed the following experimental fragments:

1. $C_{15}H_{16}O_4N$ (H_2O lost) of *m/z*=274.1042 [Δ(*m/z*)=+0.0037]
2. $C_{14}H_{16}O_2N$ (CH_2O_3 lost) of *m/z*=230.1158 [Δ(*m/z*)=+0.0023]
3. $C_7H_{10}O_4N$ (C_8H_8O lost) of *m/z*=172.0614 [Δ(*m/z*)=−0.0004]
4. $C_7H_8O_3N$ ($C_8H_{10}O_2$ lost) of *m/z*=154.0479 [Δ(*m/z*)=+0.0025]

Upon analysis by ACD/MS Fragmenter software these fragment could be formed by phenatic acid A only.
Analysis of fragments for t_{R2}=6.31 min due to low signal of this metabolite did not reveal whether it was obscurolide C2 or streptoglutarimide C.

Identification of the product at *m/z*=274.1042 (ESI$^-$)

Also at t_{R2}=6.31 min there was an [M−H]$^-$ ion of *m/z*=274.1042 and [M−H]$^-$ formula $C_{15}H_{16}O_4N$. It differed from phenatic acid by mass of water (and such an ion was observed at phenatic acid retention time). However in this case its elution is at t_{R2}=6.31 min, the same time in which either obscurolide C2 or streptoglutarimide C (ultimately they were not distinguished) were eluted. The analysis with the use of ACD/MS Fragmenter revealed that neither obscurolide C2 nor streptoglutarimide C could lose water molecule in negative ionisation. What is more, two characteristic fragments of *m/z* 230.1158 and 163.0753 were observed and upon the simulation of fragmentation by ACD/MS Fragmenter. They could not be attributed either to obscurolide C2 or streptoglutarimide C. It unfortunately meant that at least two various molecules must have had the same retention time.

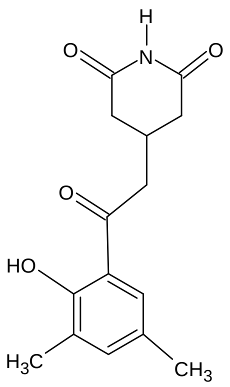

3-(2-Hydroxy-3,5-dimethylphenacyl)glutarimide

That is why *m/z*=274.1042 was treated as an individual molecule and out of *Streptomyces* metabolites a very common metabolite 3-(2-hydroxy-3,5-dimethylphenacyl)glutarimide also called actiphenol could be attributed to this mass.

The analysis of fragments with the use of ACD/MS Fragmenter revealed that:

1. $C_{14}H_{16}O_2N$ (CO_2 lost) of *m/z* 230.1158 [$\Delta(m/z)$=+0.0023]
2. $C_{10}H_{11}O_2$ ($C_5H_5O_2N$ lost) of *m/z* 163.0753 [$\Delta(m/z)$=+0.0006]

come from parent compound actiphenol (to be more precise 3-(2-Hydroxy-3,5-dimethylphenacyl)glutarimide).

Actiphenol, phenatic acid and cycloheximide are originated from the same module polyketide synthase and their simultaneous presence in the cultivation broth is another evidence for the correct identification of these metabolites

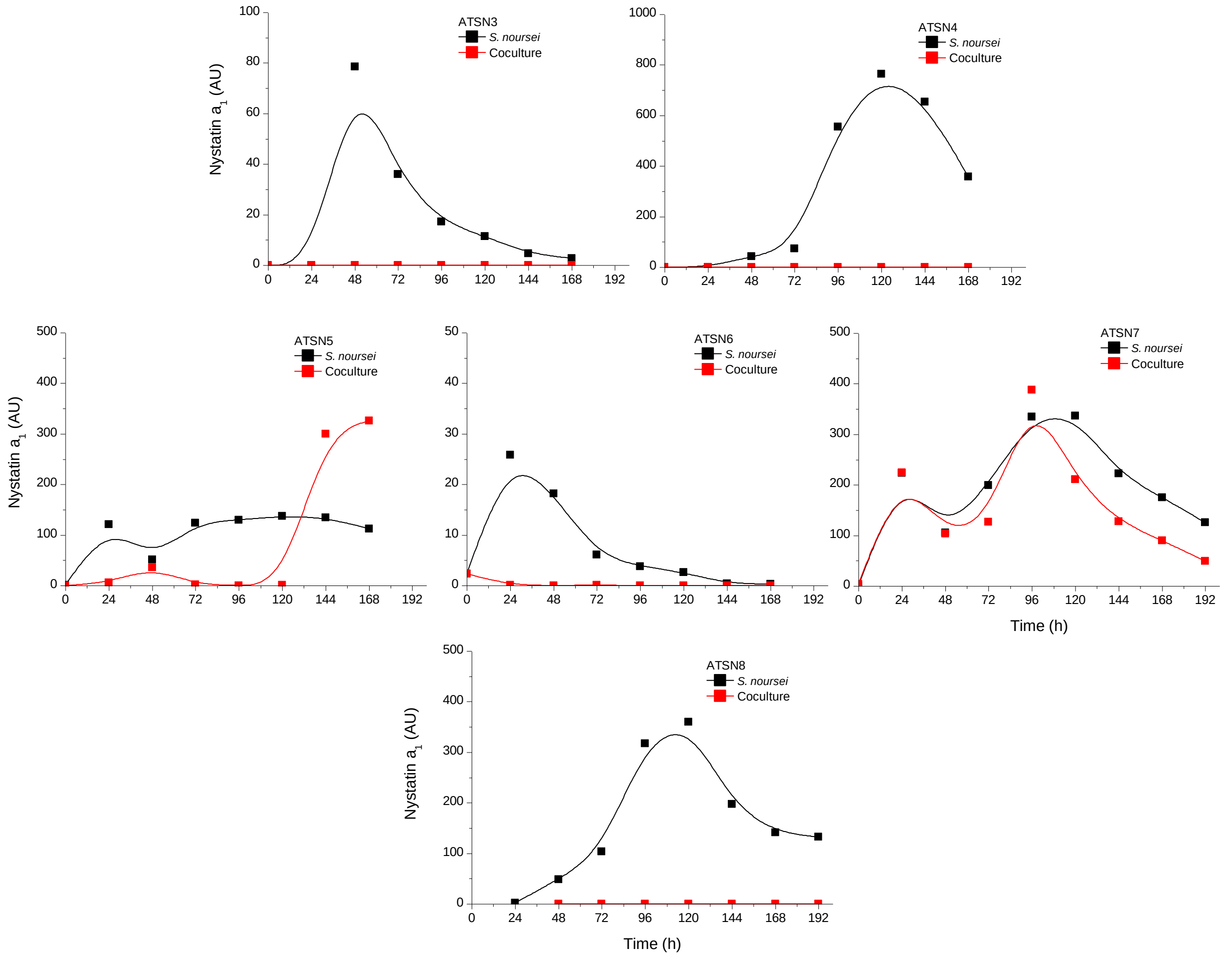

Fig. S1 Time courses of nystatin A_1 production in the *Aspergillus terreus* and *Streptomyces noursei* co-cultures and the corresponding monoculture controls of *S. noursei*. AU-auxiliary units

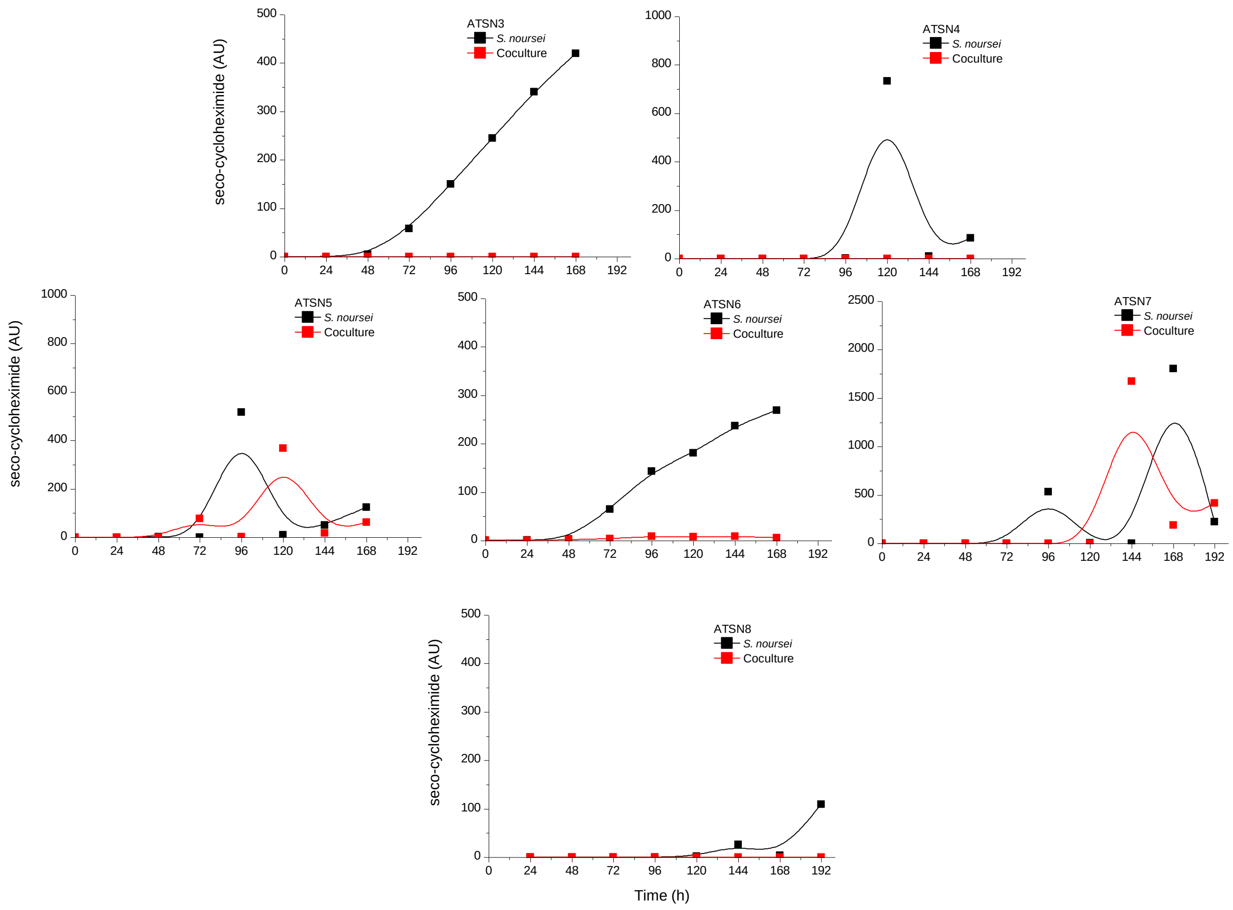

Fig. S2 Time courses of seco-cycloheximide production in the *Aspergillus terreus* and *Streptomyces noursei* co-cultures and the corresponding monoculture controls of *S. noursei*. AU-auxiliary units

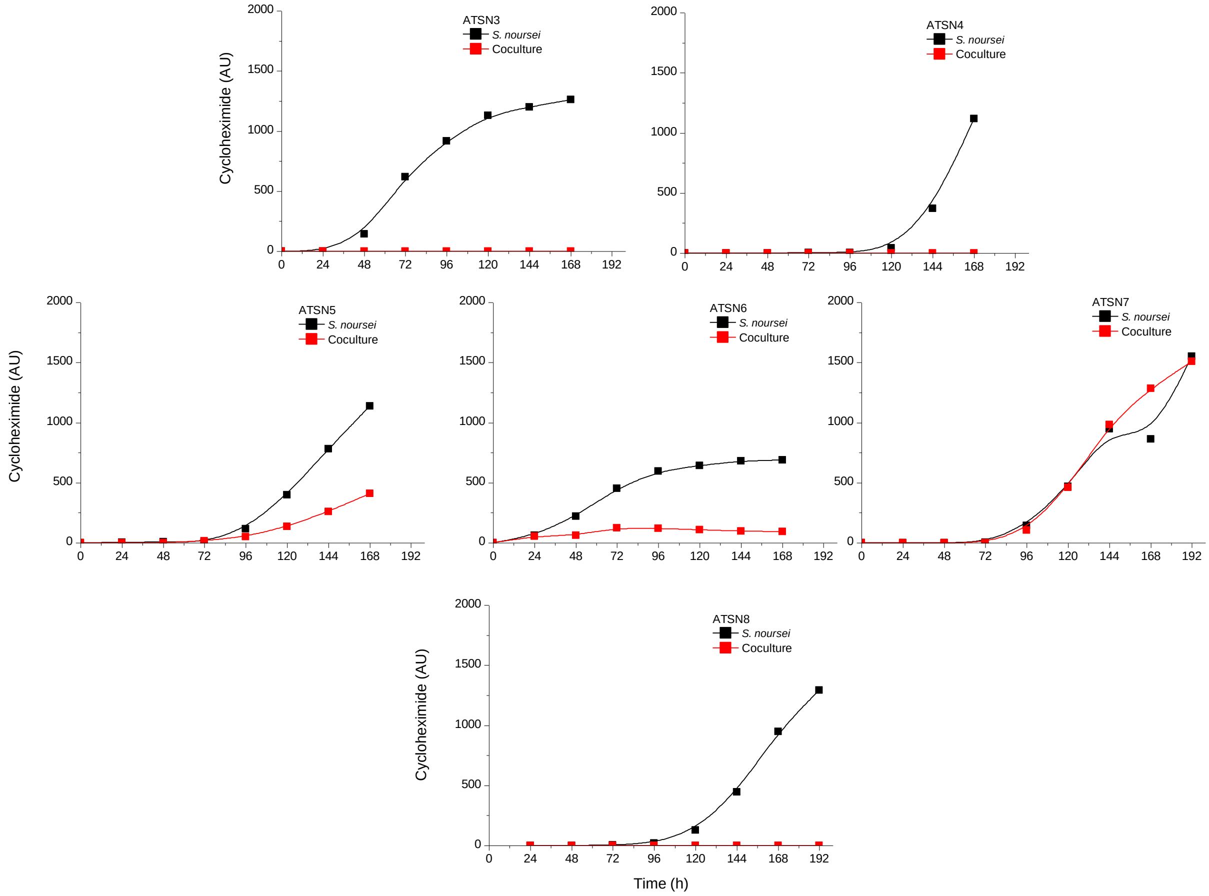

Fig. S3 Time courses of cycloheximide production in the *Aspergillus terreus* and *Streptomyces noursei* co-cultures and the corresponding monoculture controls of *S. noursei*. AU-auxiliary units

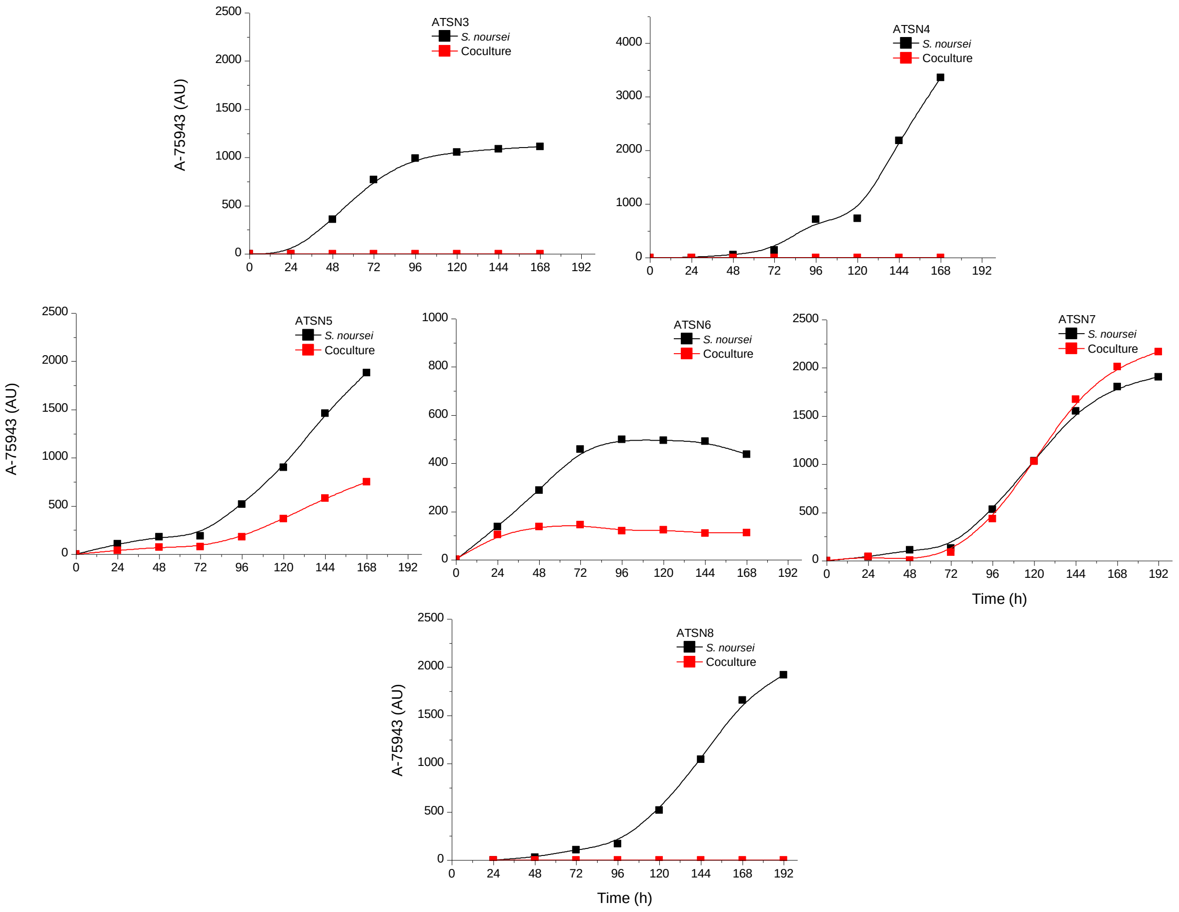

Fig. S4 Time courses of A75943 production in the *Aspergillus terreus* and *Streptomyces noursei* co-cultures and the corresponding monoculture controls of *S. noursei*. AU-auxiliary units

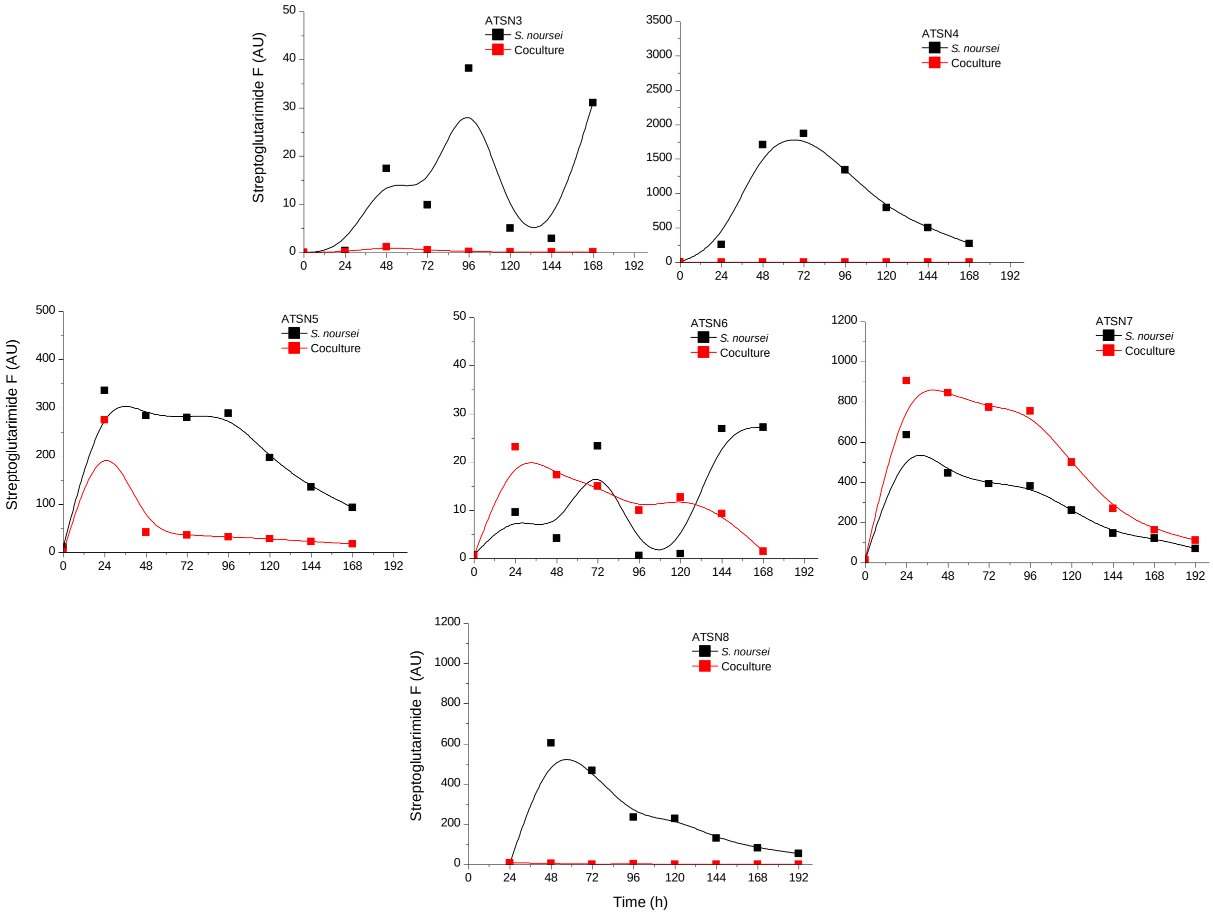

Fig. S5 Time courses of streptoglutarimide F production in the *Aspergillus terreus* and *Streptomyces noursei* co-cultures and the corresponding monoculture controls of *S. noursei*. AU-auxiliary units

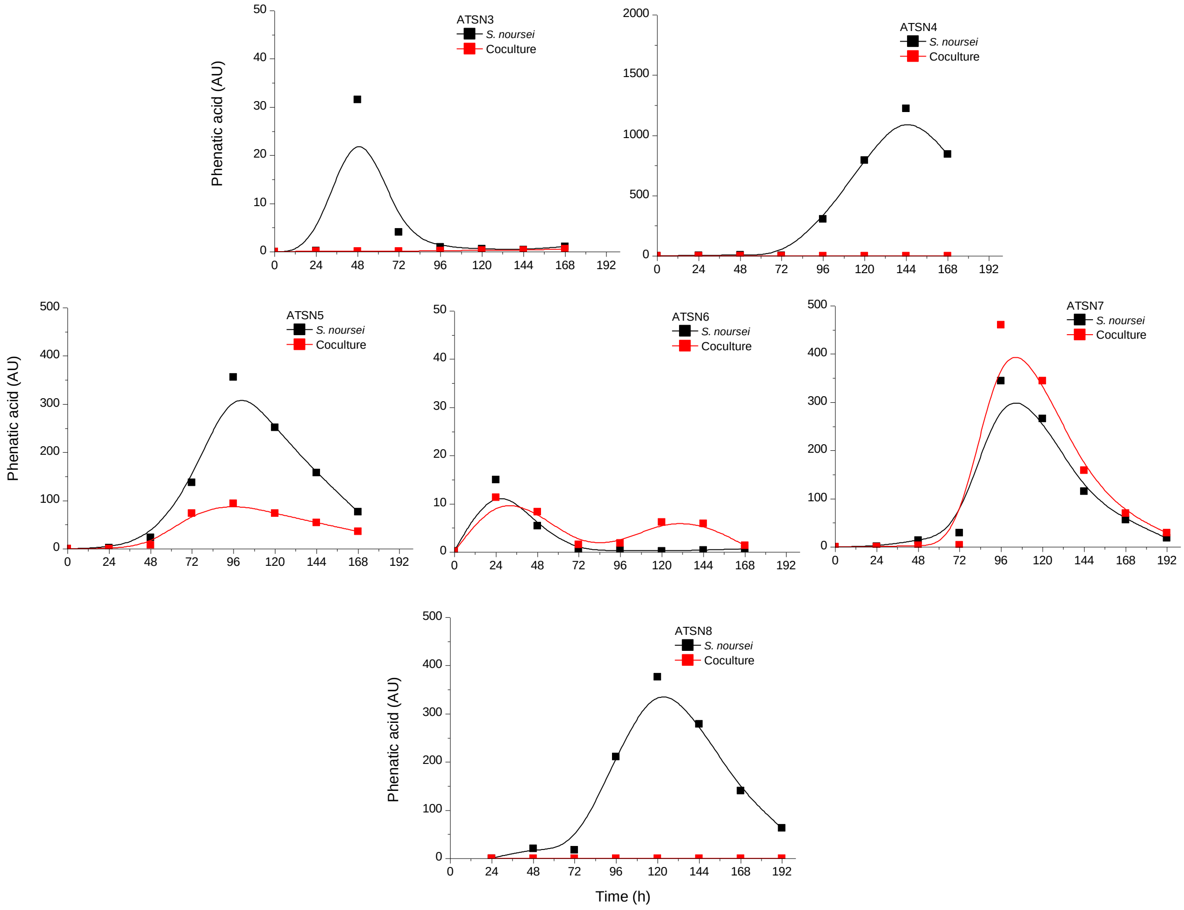

Fig. S6 Time courses of phenatic acid production in the *Aspergillus terreus* and *Streptomyces noursei* co-cultures and the corresponding monoculture controls of *S. noursei*. AU-auxiliary units

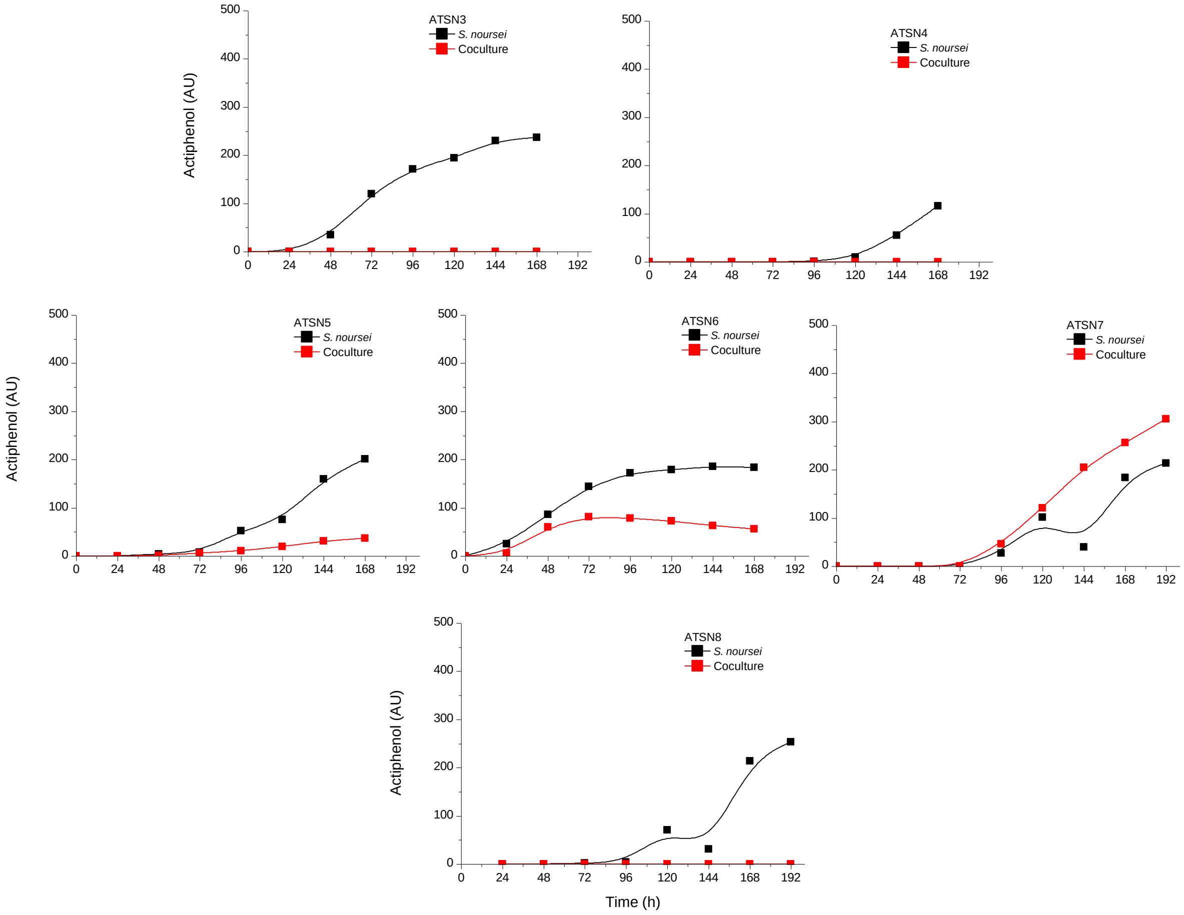

Fig. S7 Time courses of 3-(2-Hydroxy-3,5-dimethylphenacyl)glutarimide (actiphenol) production in the *Aspergillus terreus* and *Streptomyces noursei* co-cultures and the corresponding monoculture controls of *S. noursei*. AU-auxiliary units

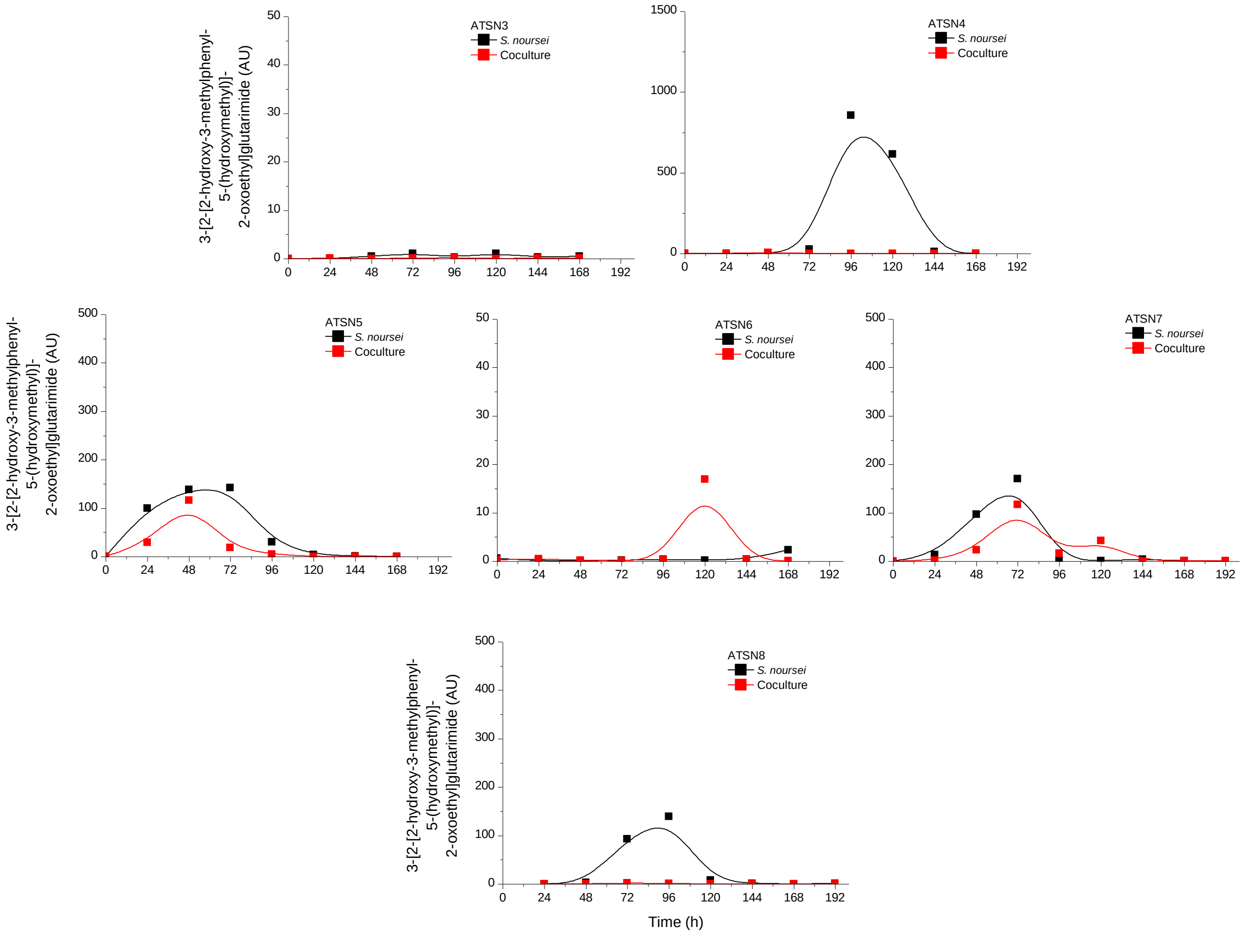

Fig. S8 Time courses of 3-[2-[2-hydroxy-3-methylphenyl-5-(hydroxymethyl)]-2-oxoethyl]glutarimide production in the *Aspergillus terreus* and *Streptomyces noursei* co-cultures and the corresponding monoculture controls of *S. noursei*. AU-auxiliary units

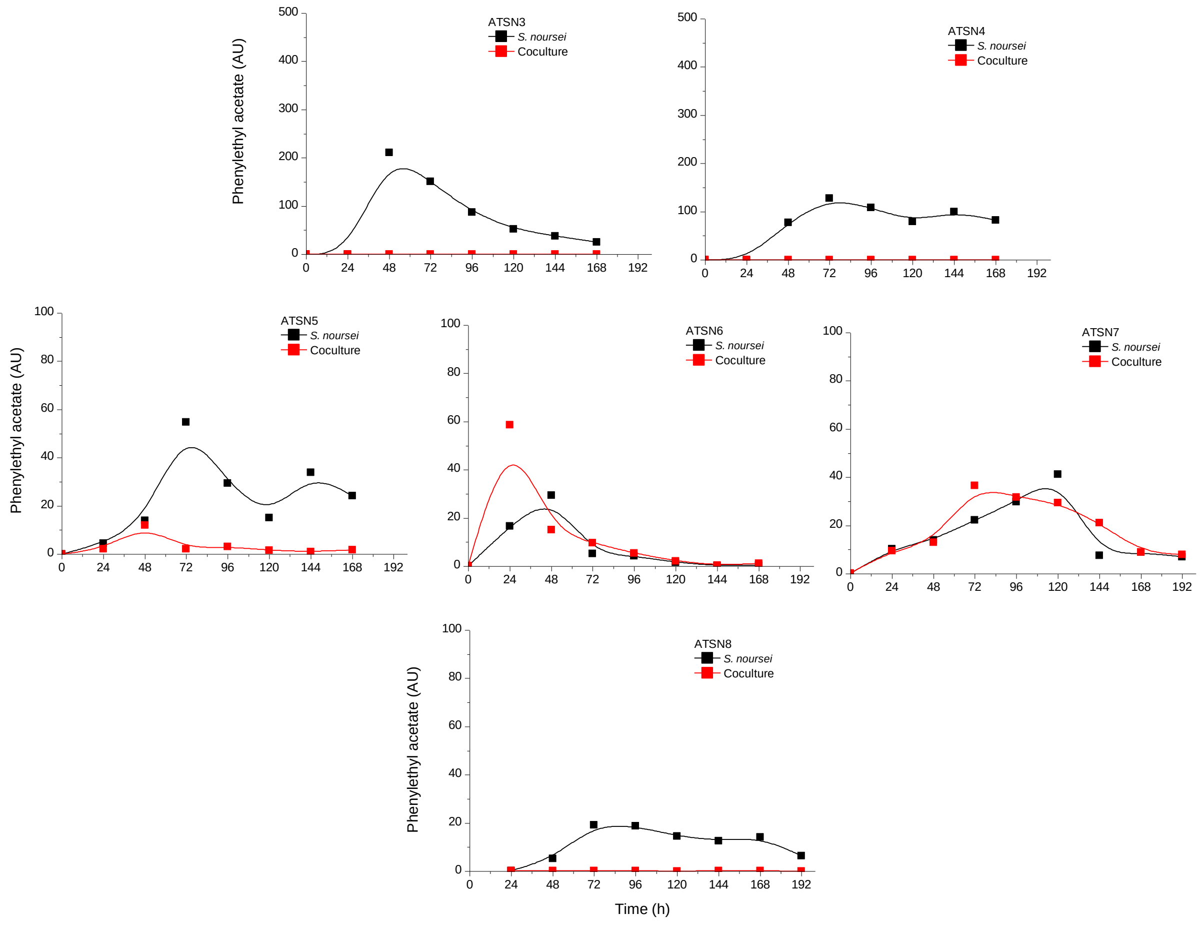

Fig. S9 Time courses of 2-phenylethyl acetate production in the *Aspergillus terreus* and *Streptomyces noursei* co-cultures and the corresponding monoculture controls of *S. noursei*. AU-auxiliary units

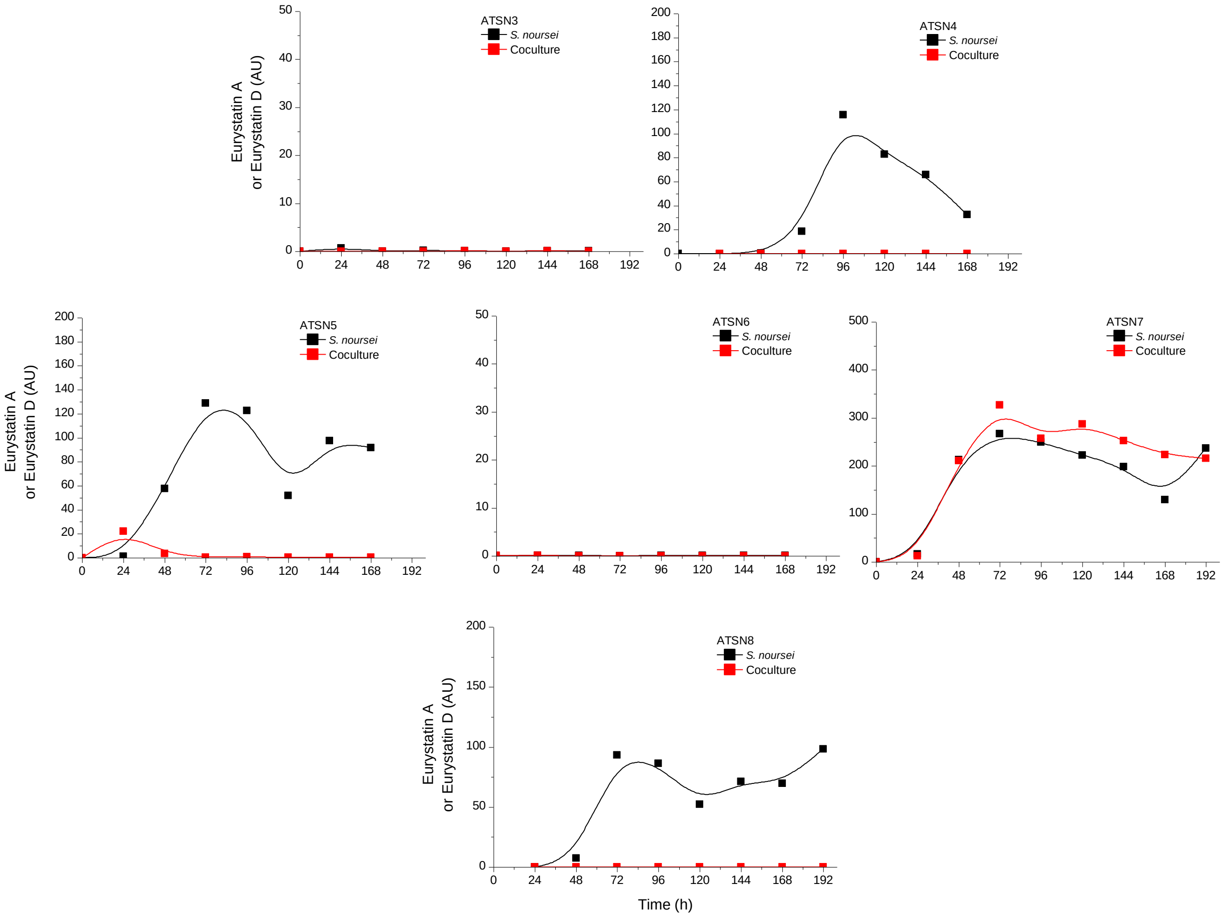

Fig. S10 Time courses of eurystatin A or D production in the *Aspergillus terreus* and *Streptomyces noursei* co-cultures and the corresponding monoculture controls of *S. noursei*. AU-auxiliary units

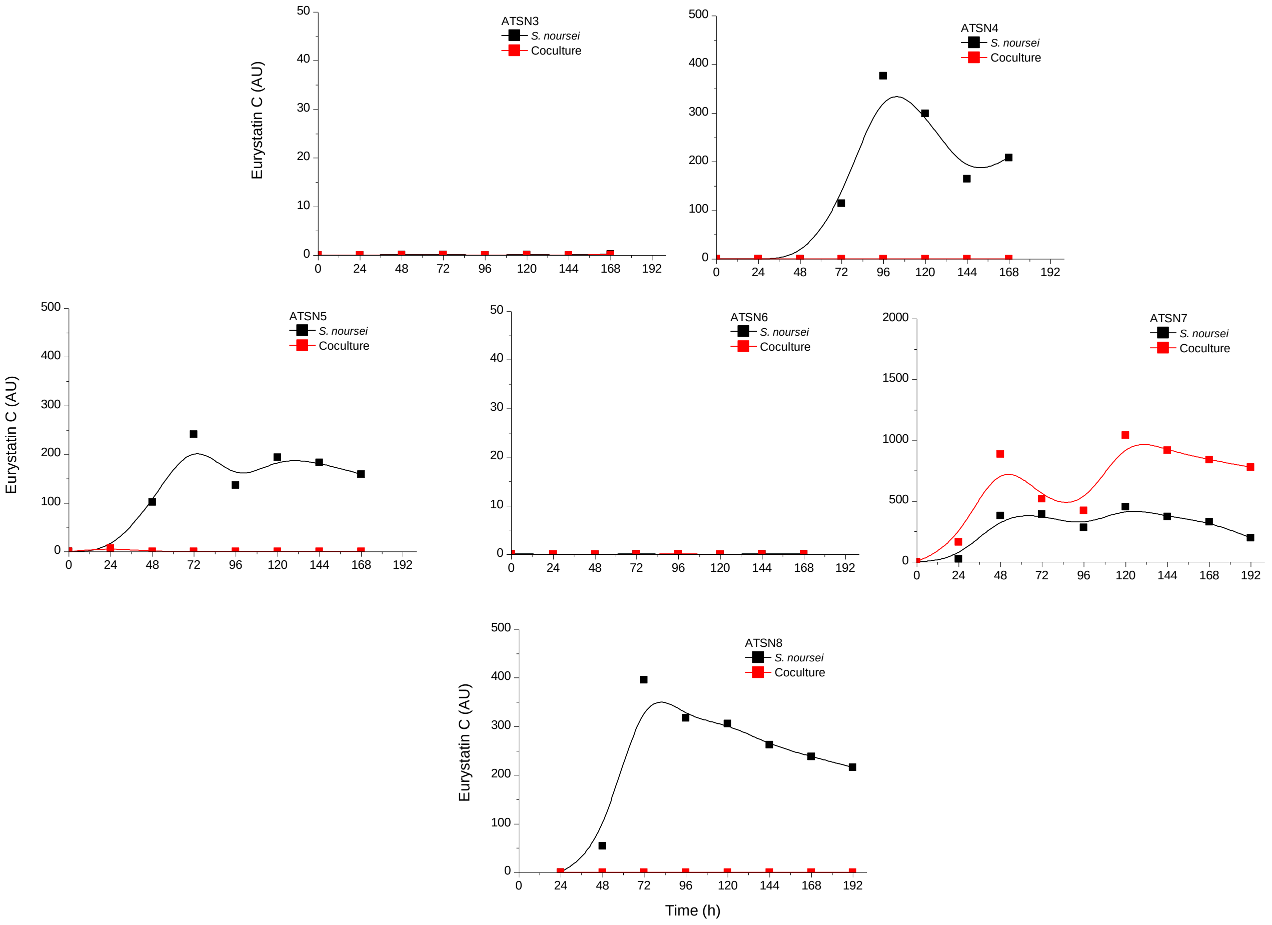

Fig. S11 Time courses of eurystatin C production in the *Aspergillus terreus* and *Streptomyces noursei* co-cultures and the corresponding monoculture controls of *S. noursei*. AU-auxiliary units

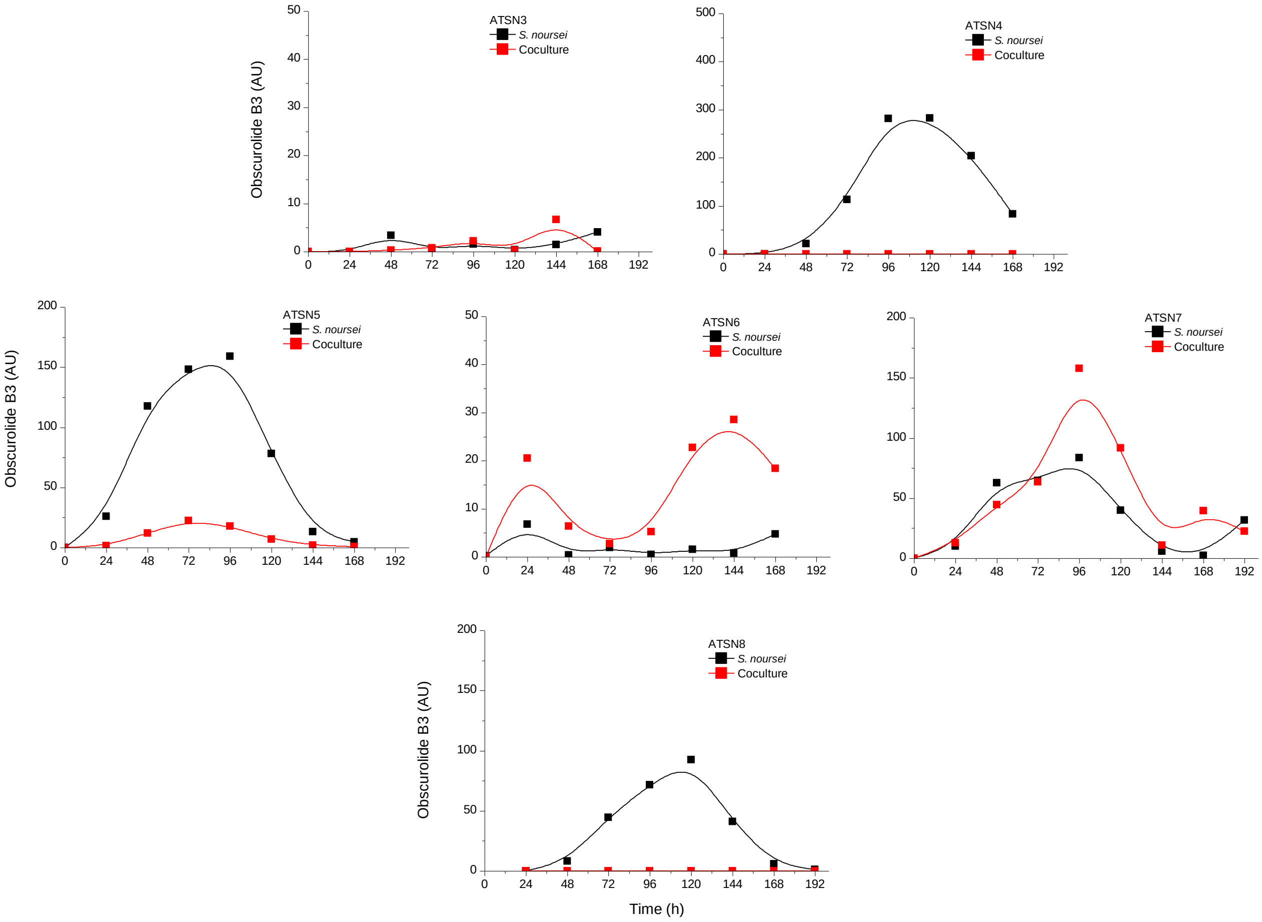

Fig. S12 Time courses of obscurolide B3 production in the *Aspergillus terreus* and *Streptomyces noursei* co-cultures and the corresponding monoculture controls of *S. noursei*. AU-auxiliary units

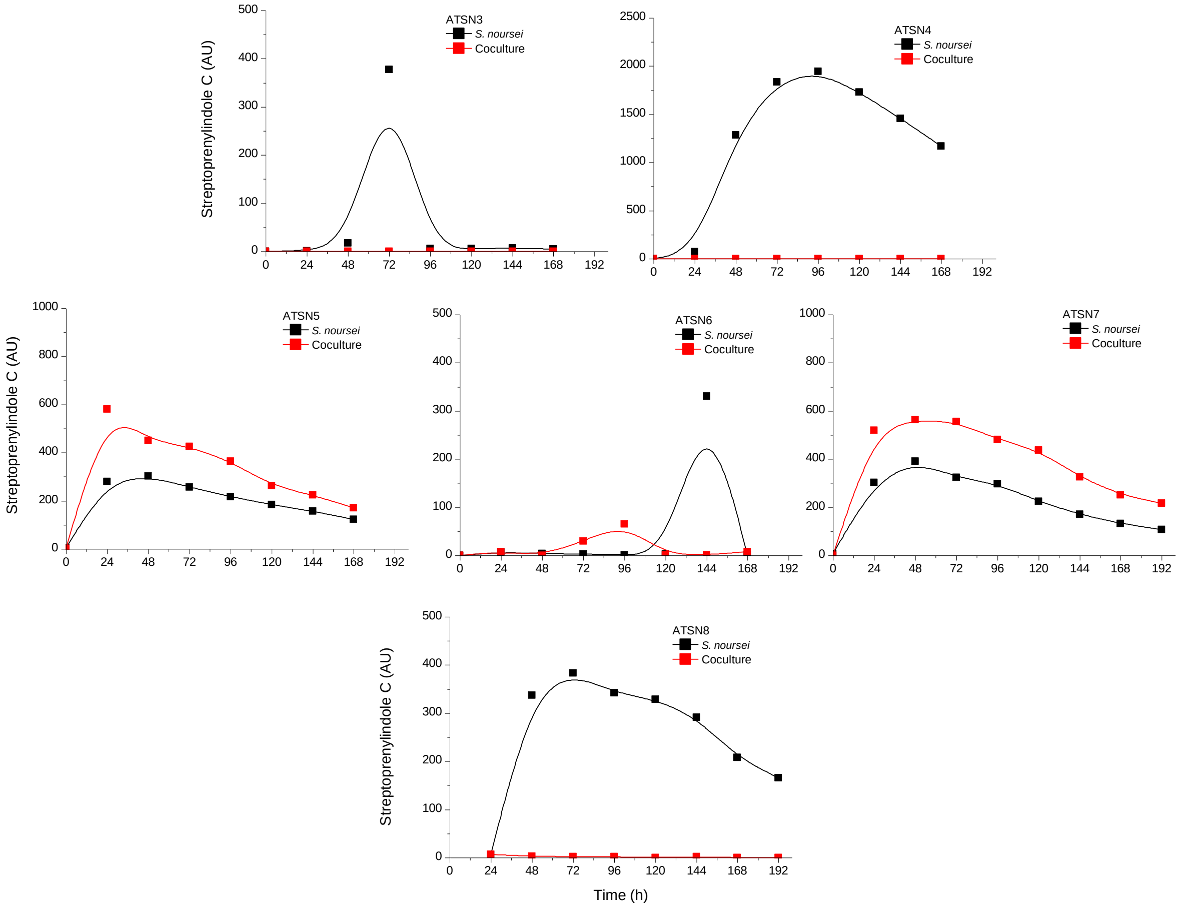

Fig. S13 Time courses of streptoprenylindole C production in the *Aspergillus terreus* and *Streptomyces noursei* co-cultures and the corresponding monoculture controls of *S. noursei*. AU-auxiliary units

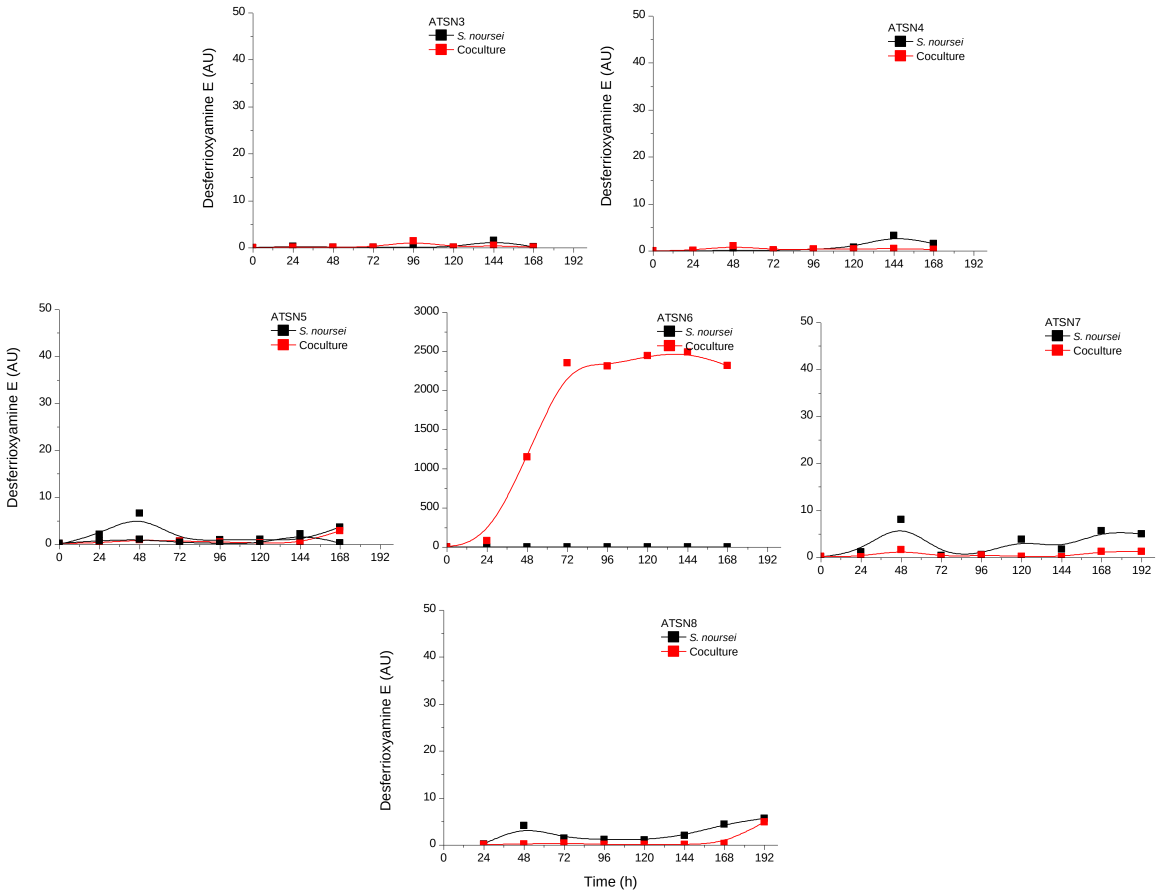

Fig. S14 Time courses of desferrioxamine E production in the *Aspergillus terreus* and *Streptomyces noursei* co-cultures and the corresponding monoculture controls of *S. noursei*. AU-auxiliary units

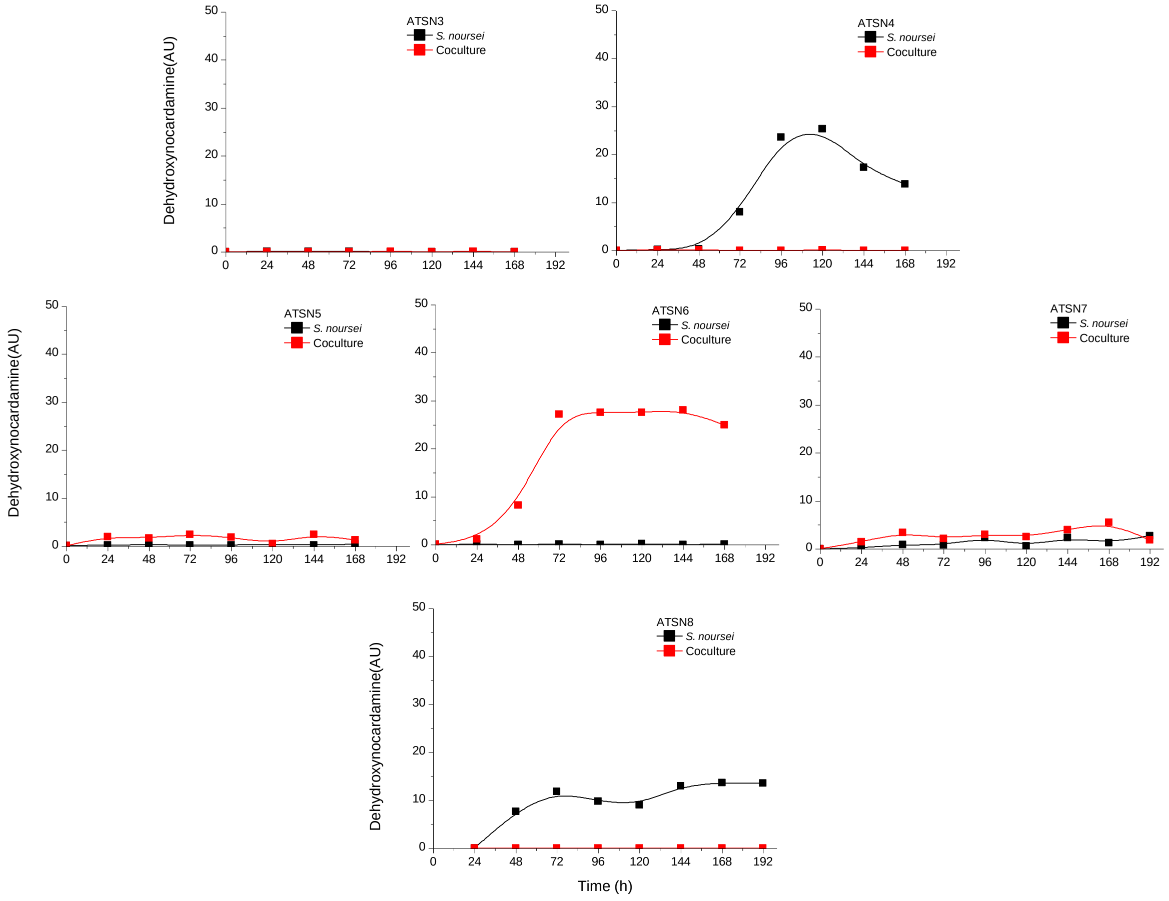

Fig. S15 Time courses of deshydroxynocardamine production in the *Aspergillus terreus* and *Streptomyces noursei* co-cultures and the corresponding monoculture controls of *S. noursei*. AU-auxiliary units

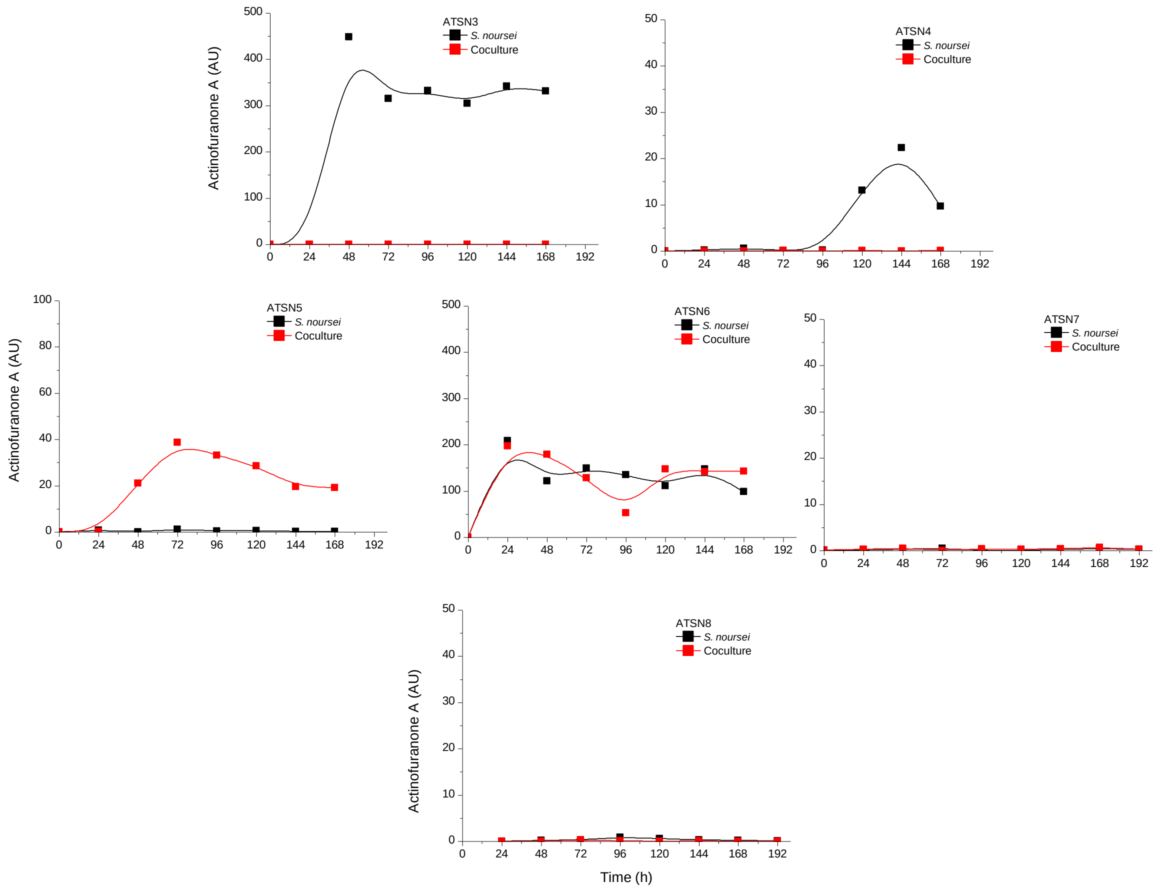

Fig. S16 Time courses of actinofuranone A production in the *Aspergillus terreus* and *Streptomyces noursei* co-cultures and the corresponding monoculture controls of *S. noursei*. AU-auxiliary units

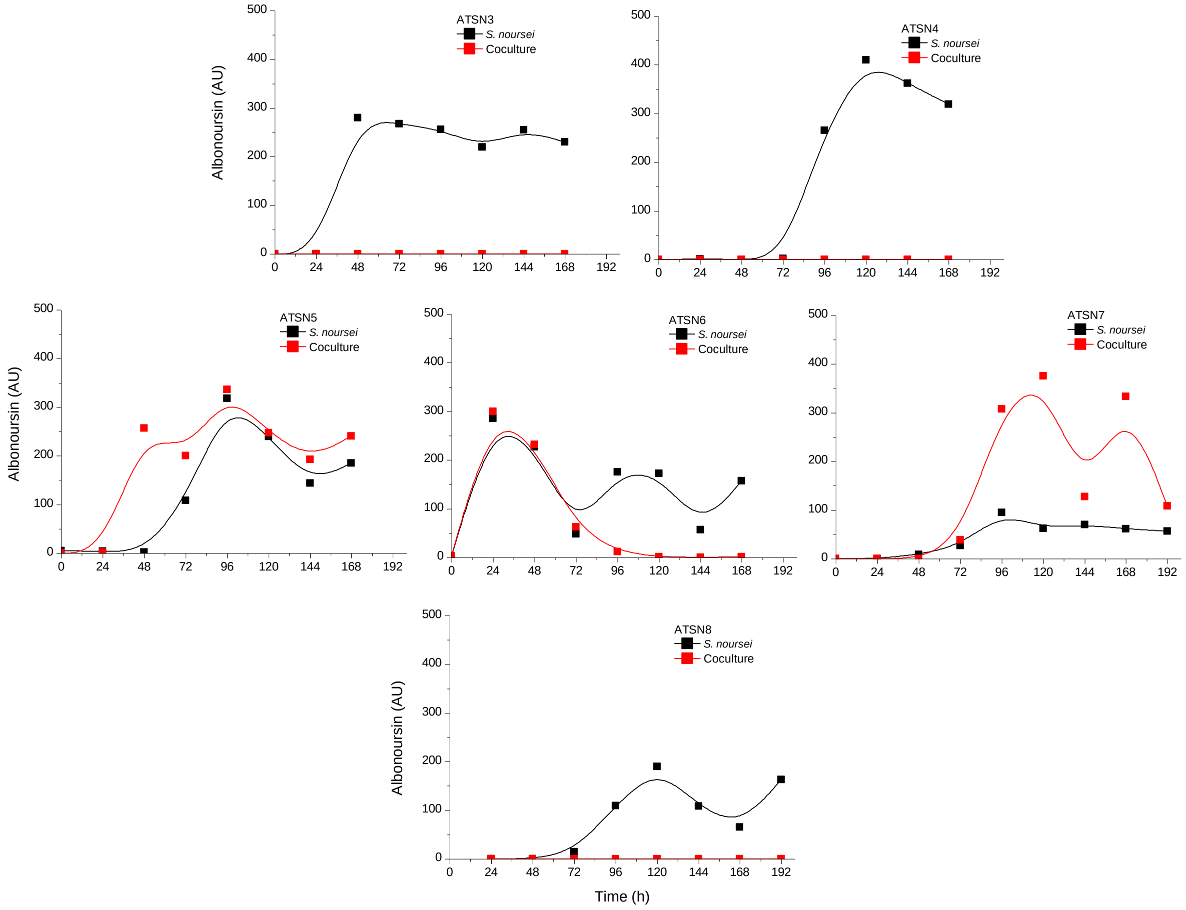

Fig. S17 Time courses of albonoursin production in the *Aspergillus terreus* and *Streptomyces noursei* co-cultures and the corresponding monoculture controls of *S. noursei*. AU-auxiliary units

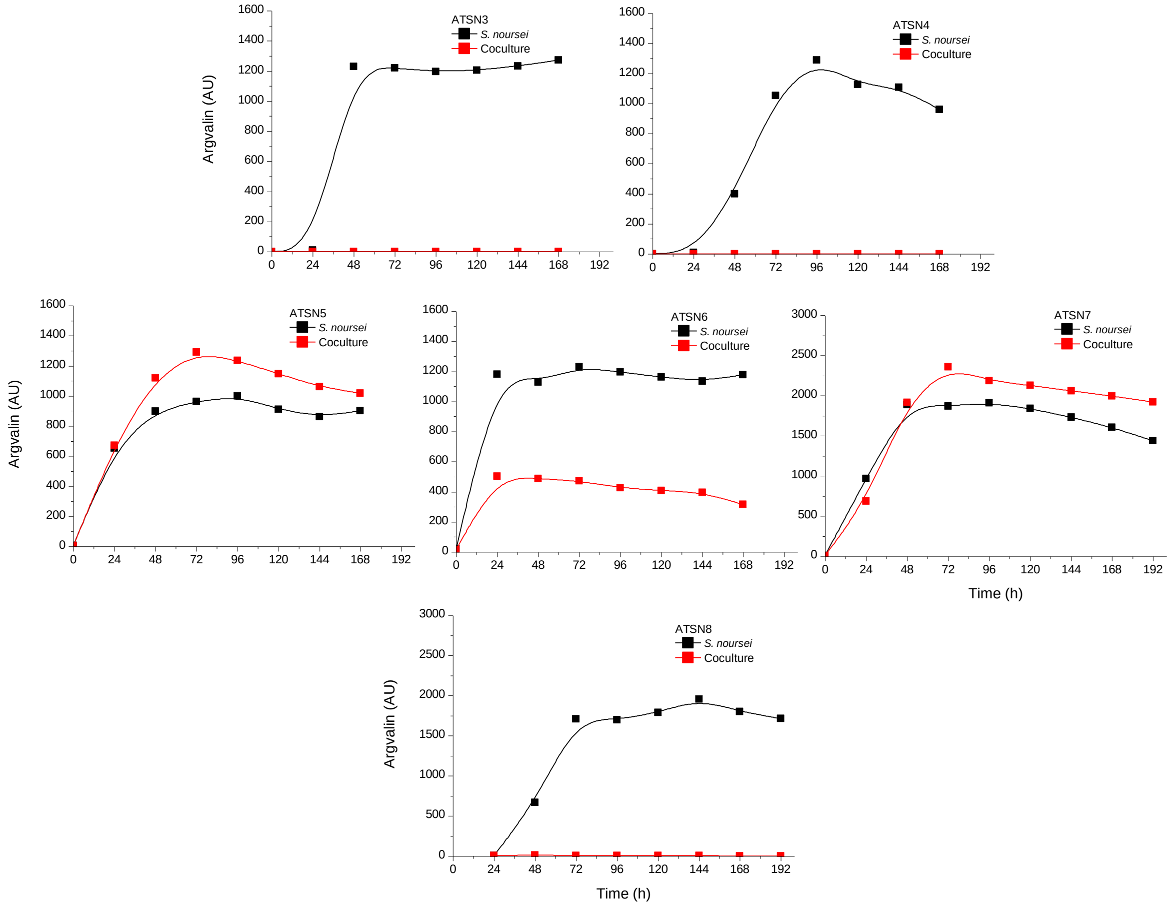

Fig. S18 Time courses of argvalin production in the *Aspergillus terreus* and *Streptomyces noursei* co-cultures and the corresponding monoculture controls of *S. noursei*. AU-auxiliary units

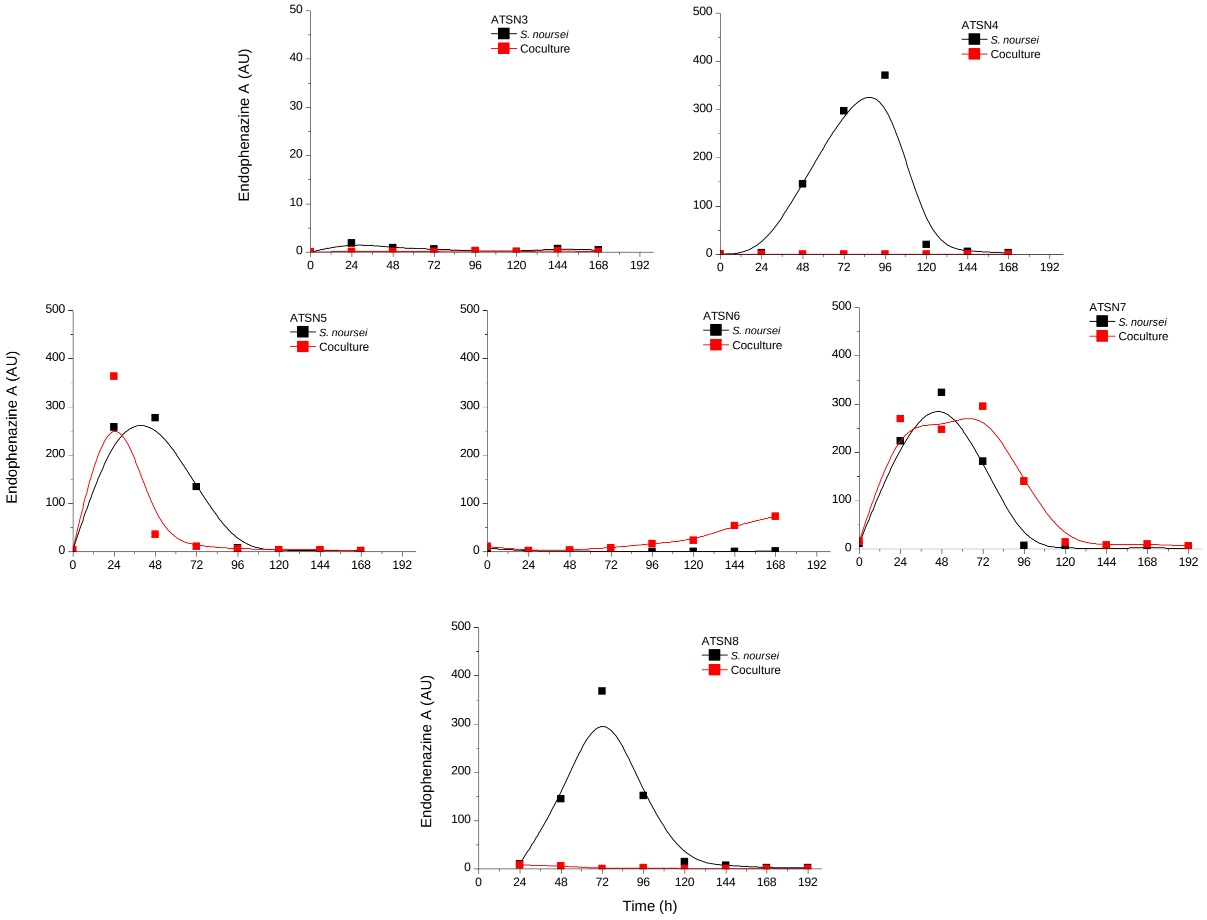

Fig. S19 Time courses of endophenazine A production in the *Aspergillus terreus* and *Streptomyces noursei* co-cultures and the corresponding monoculture controls of *S. noursei*. AU-auxiliary units

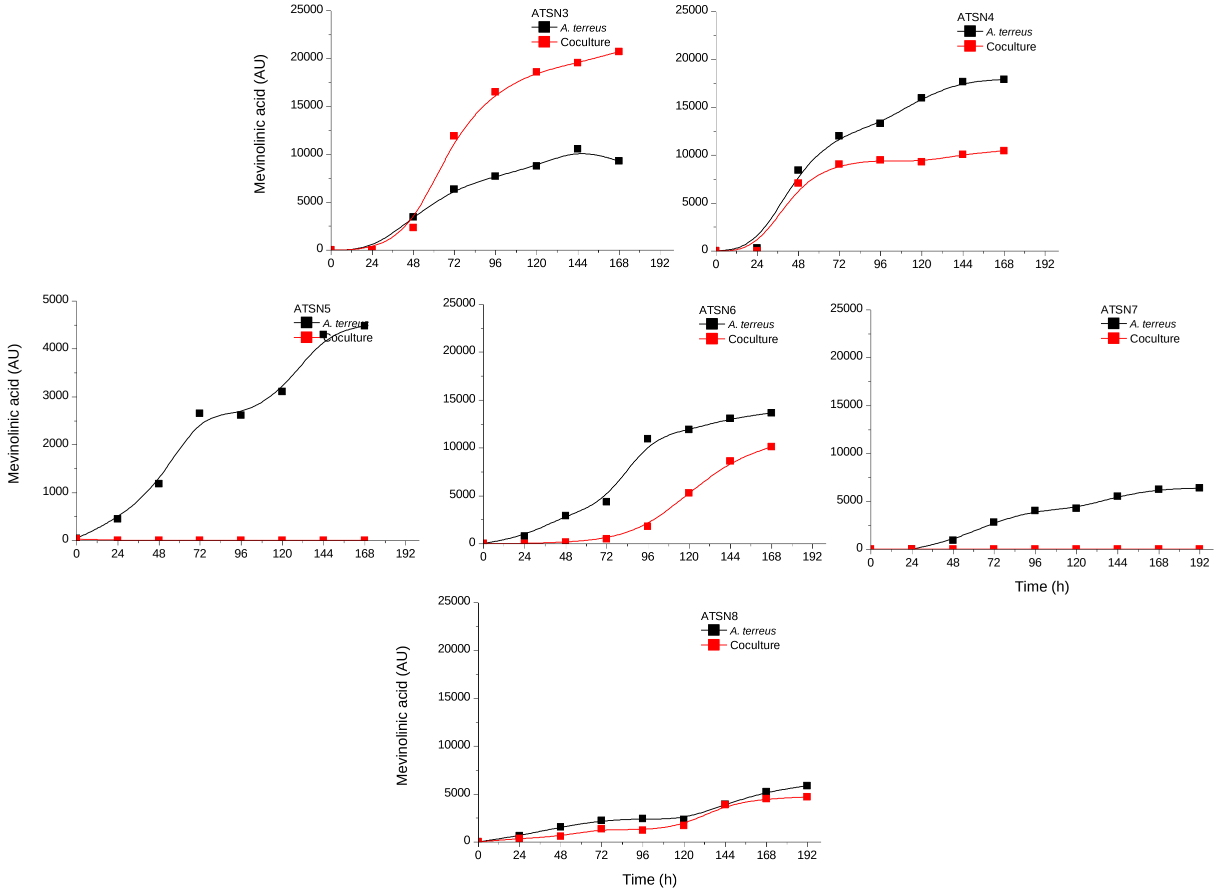

Fig. S20 Time courses of on mevinolinic acid production in the *Aspergillus terreus* and *Streptomyces noursei* co-cultures and the corresponding monoculture controls of *A. terreus*. AU-auxiliary units

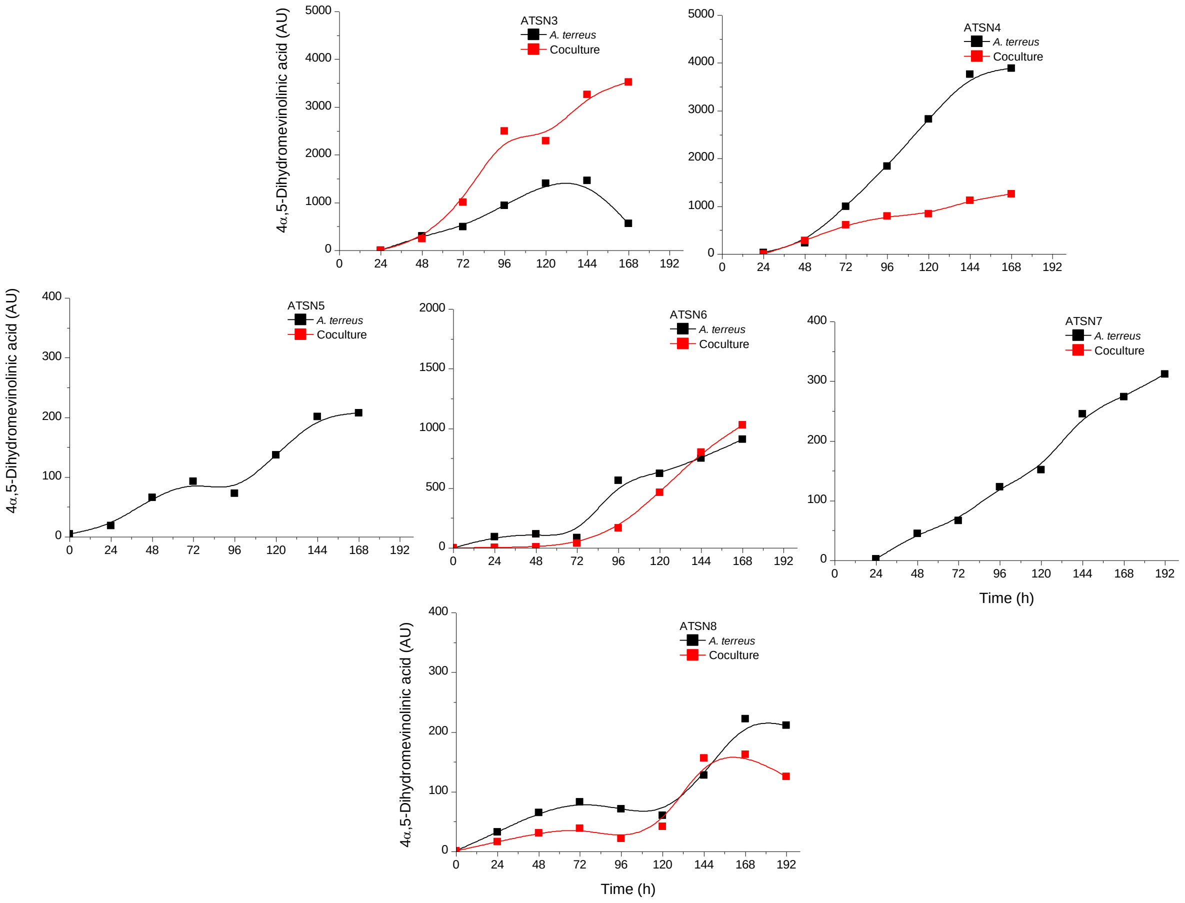

Fig. S21 Time courses of 4a,5-dihydromevinolinic production in the *Aspergillus terreus* and *Streptomyces noursei* co-cultures and the corresponding monoculture controls of *A. terreus*. AU-auxiliary units

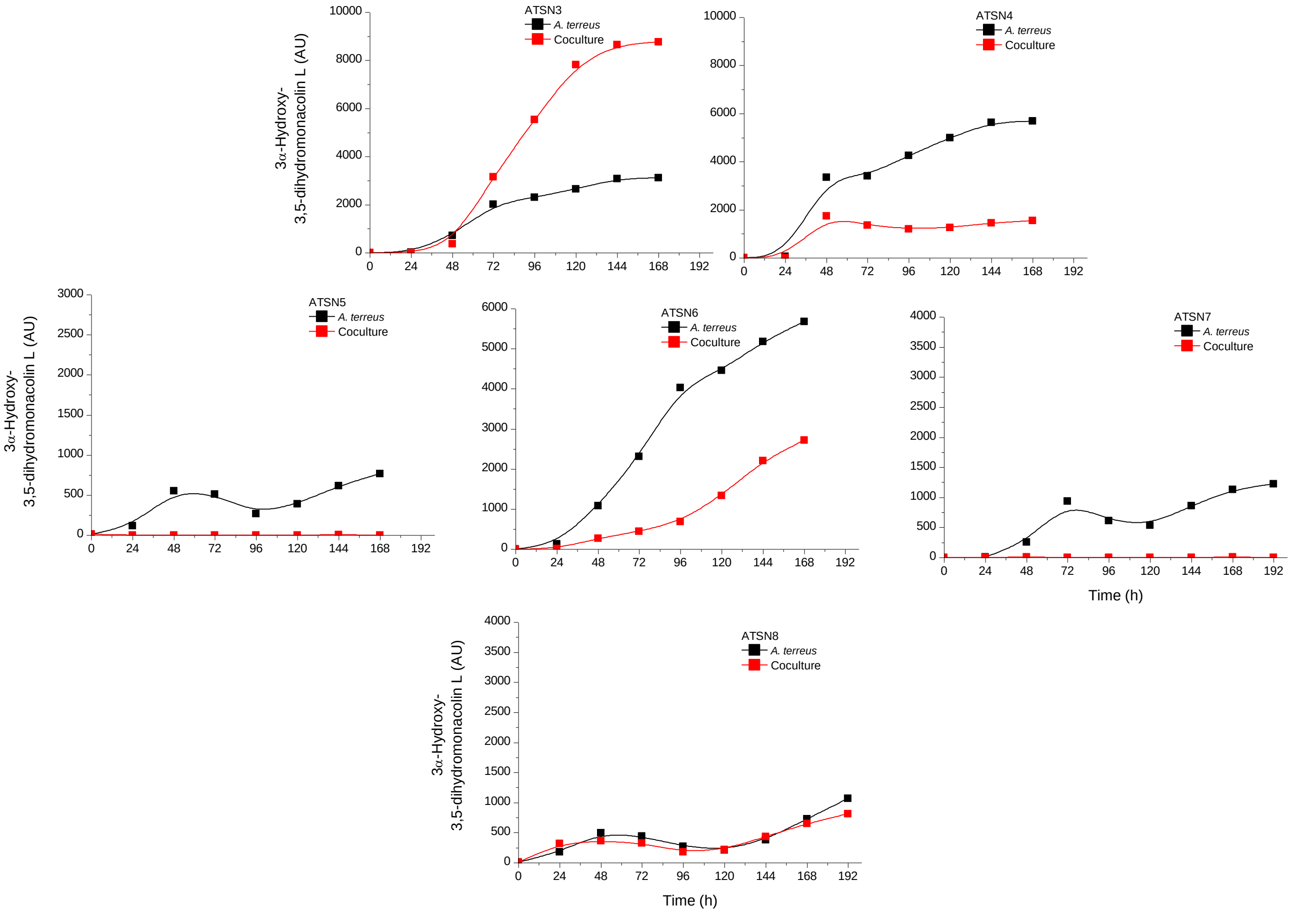

Fig. S22 Time courses of 3α-hydroxy-3,5-dihydromonacolin L production in the *Aspergillus terreus* and *Streptomyces noursei* co-cultures and the corresponding monoculture controls of *A. terreus*. AU-auxiliary units

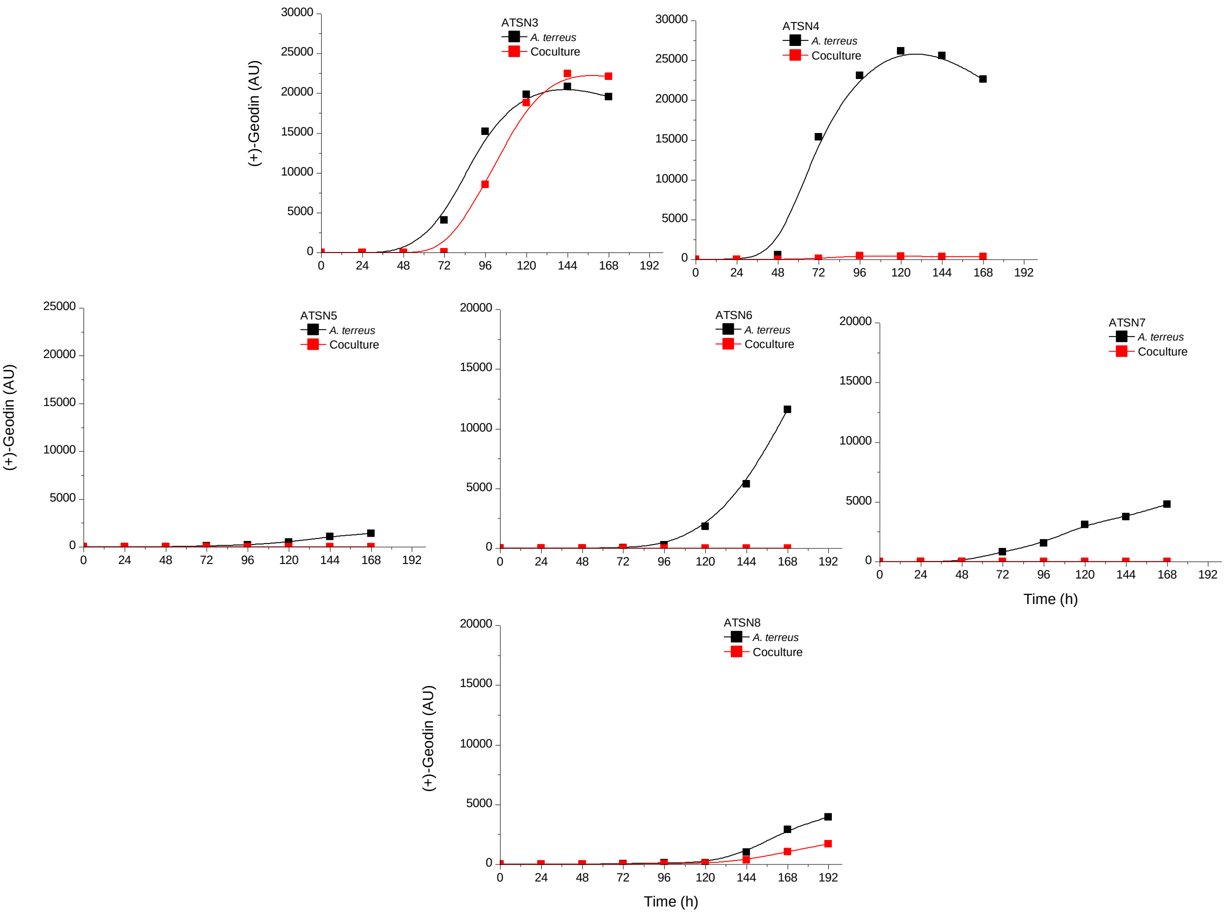

Fig. S23 Time courses of (+)-geodin production in the *Aspergillus terreus* and *Streptomyces noursei* co-cultures and the corresponding monoculture controls of *A. terreus*. AU-auxiliary units

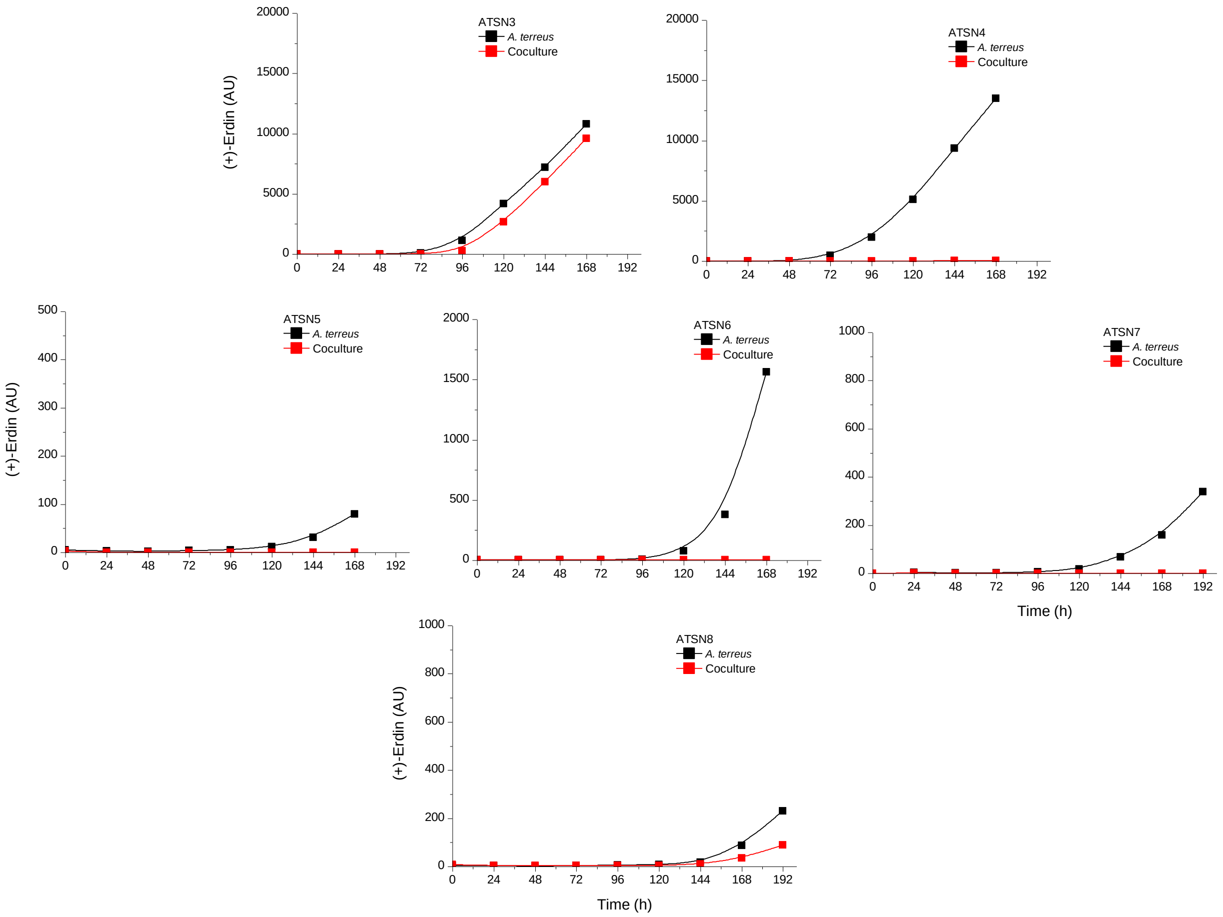

Fig. S24 Time courses of (+)-erdin production in the *Aspergillus terreus* and *Streptomyces noursei* co-cultures and the corresponding monoculture controls of *A. terreus*. AU-auxiliary units

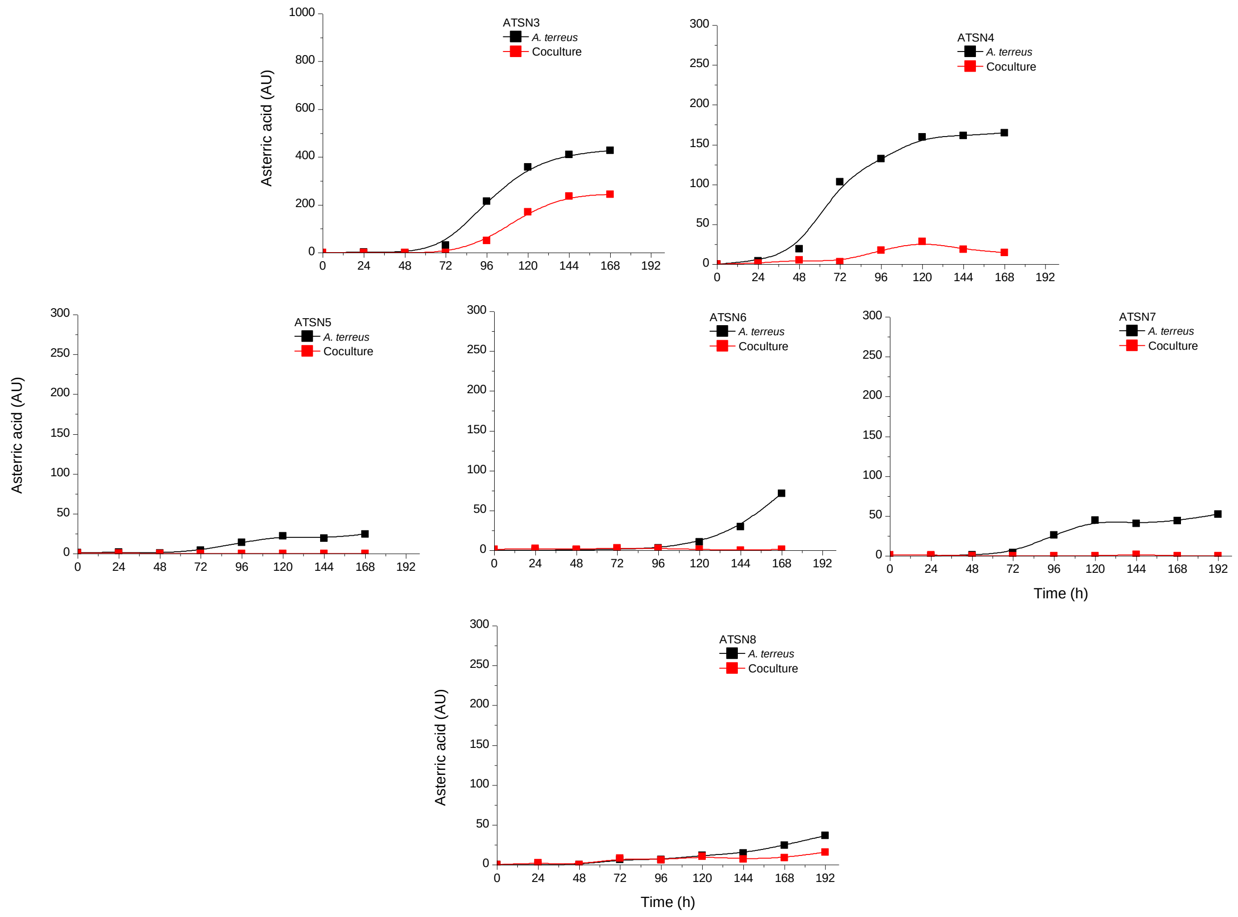

Fig. S25 Time courses of asterric acid production in the *Aspergillus terreus* and *Streptomyces noursei* co-cultures and the corresponding monoculture controls of *A. terreus*. AU-auxiliary units

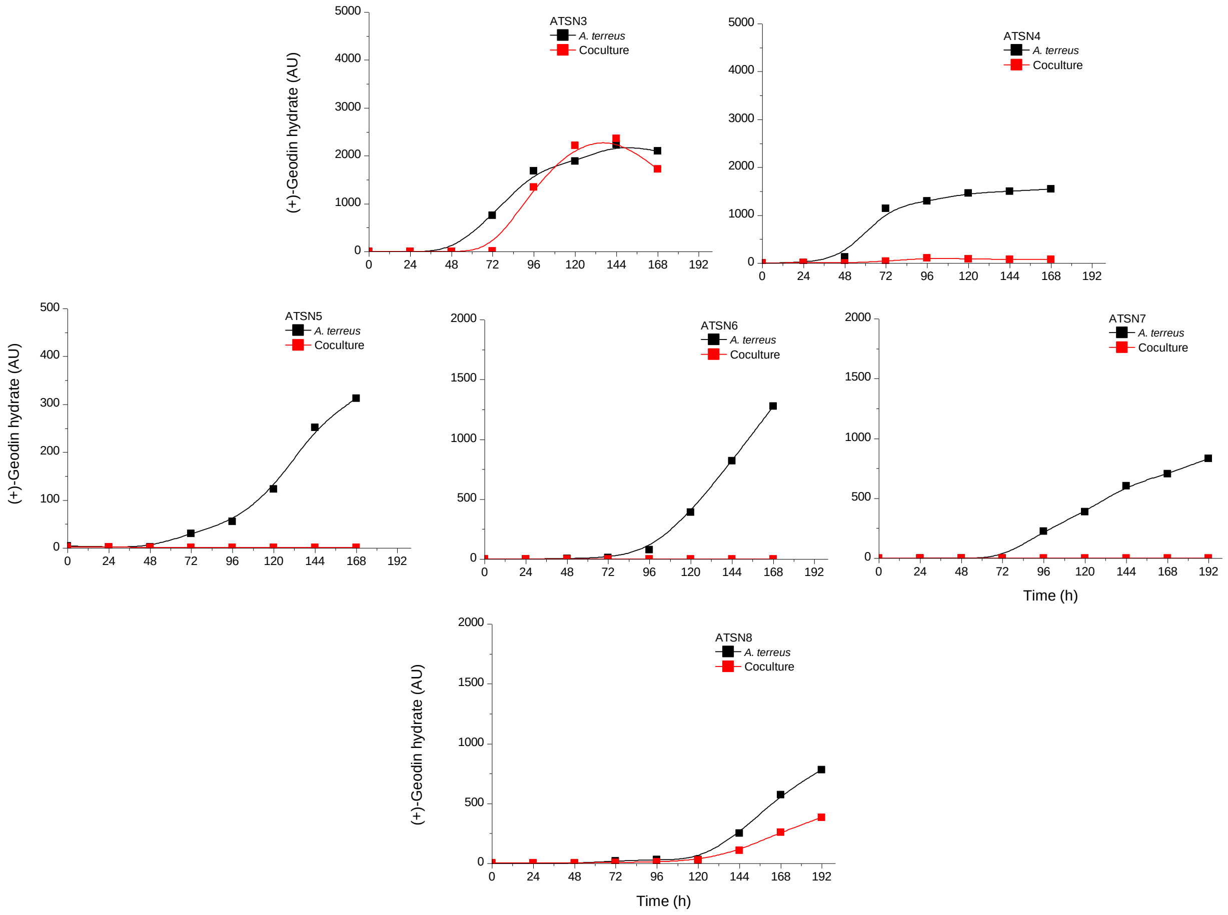

Fig. S26 Time courses of (+)-geodin hydrate production in the *Aspergillus terreus* and *Streptomyces noursei* co-cultures and the corresponding monoculture controls of *A. terreus*. AU-auxiliary units

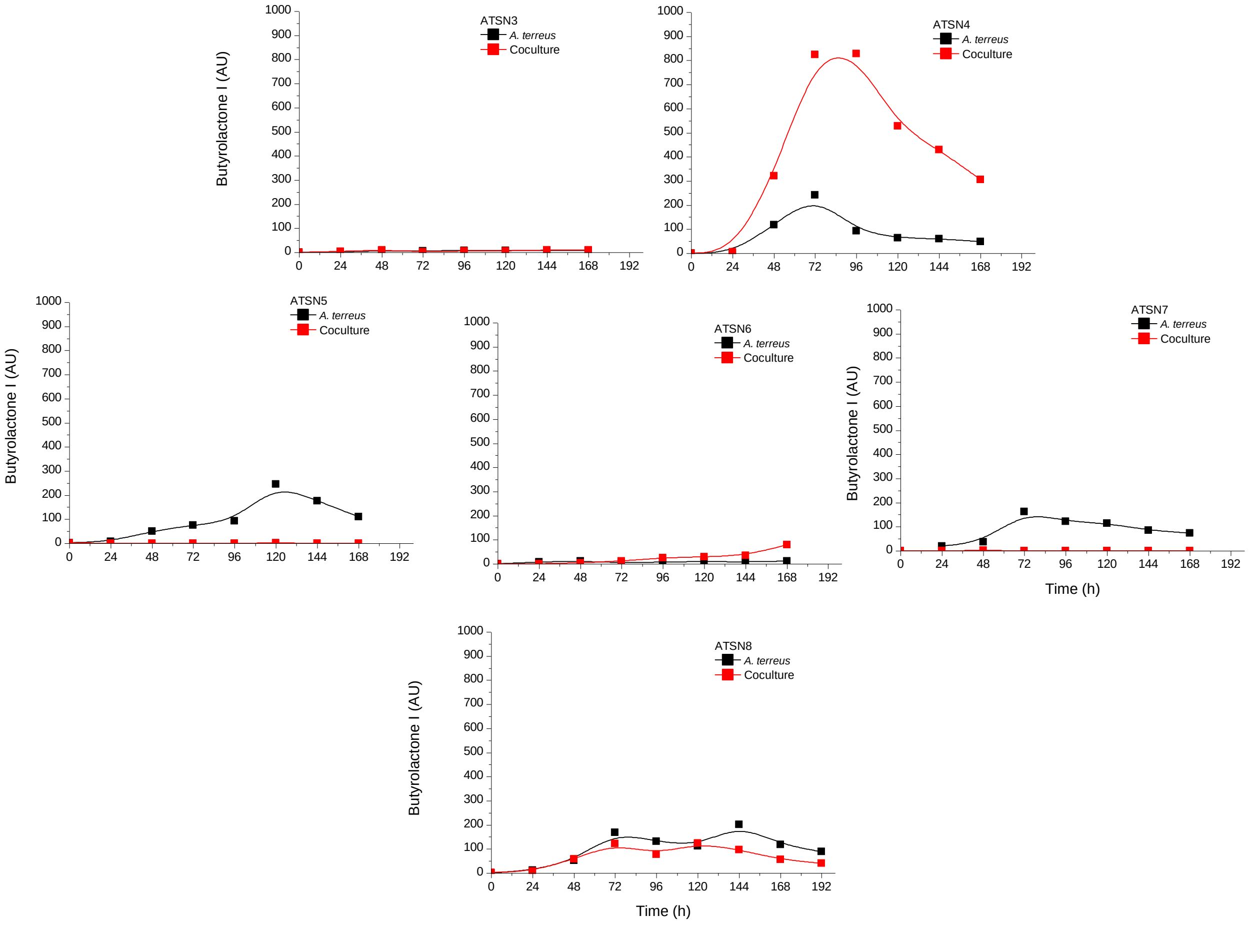

Fig. S27 Time courses of butyrolactone I production in the *Aspergillus terreus* and *Streptomyces noursei* co-cultures and the corresponding monoculture controls of *A. terreus*. AU-auxiliary units

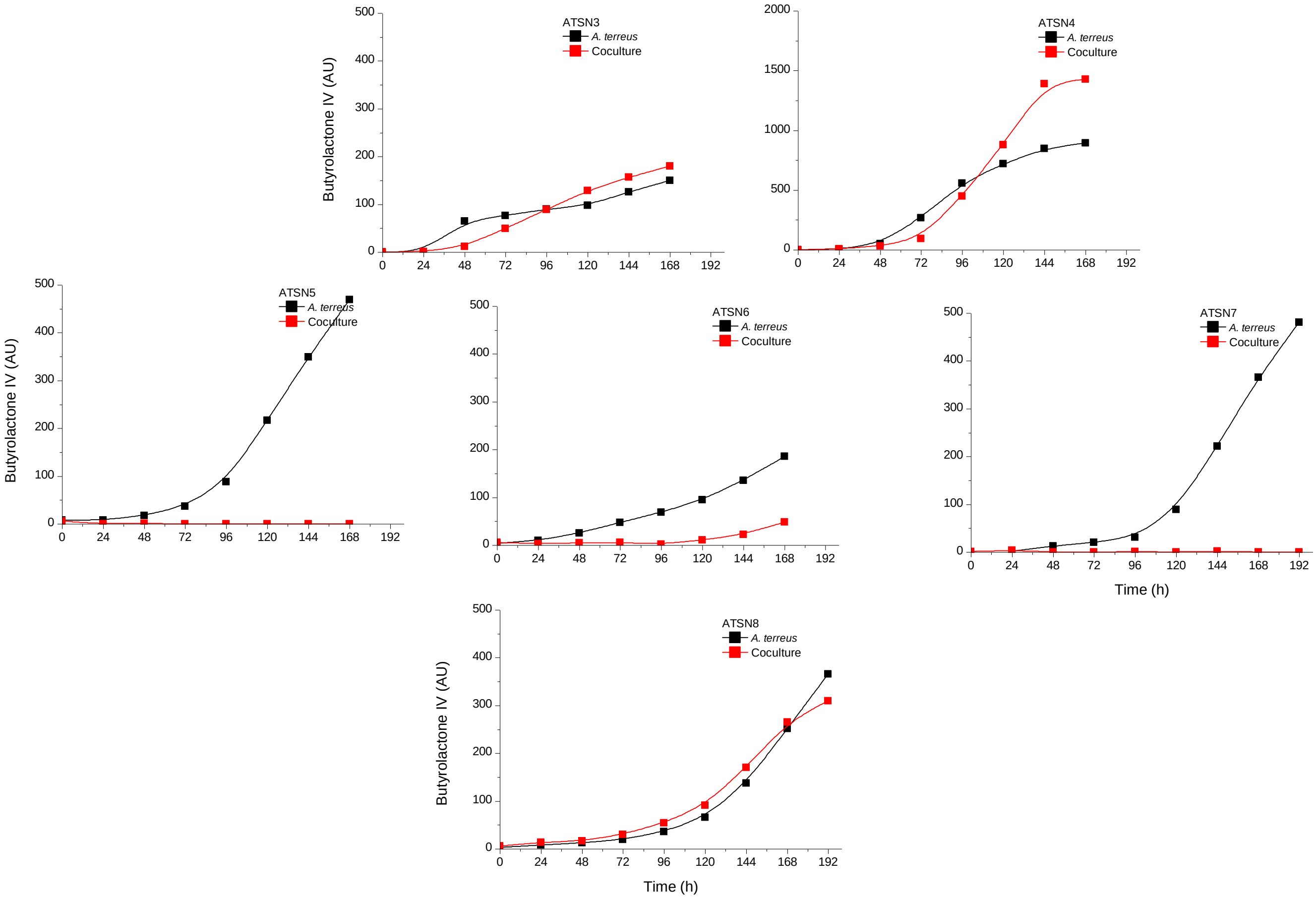

Fig. S28 Time courses of butyrolactone IV production in the *Aspergillus terreus* and *Streptomyces noursei* co-cultures and the corresponding monoculture controls of *A. terreus*. AU-auxiliary units

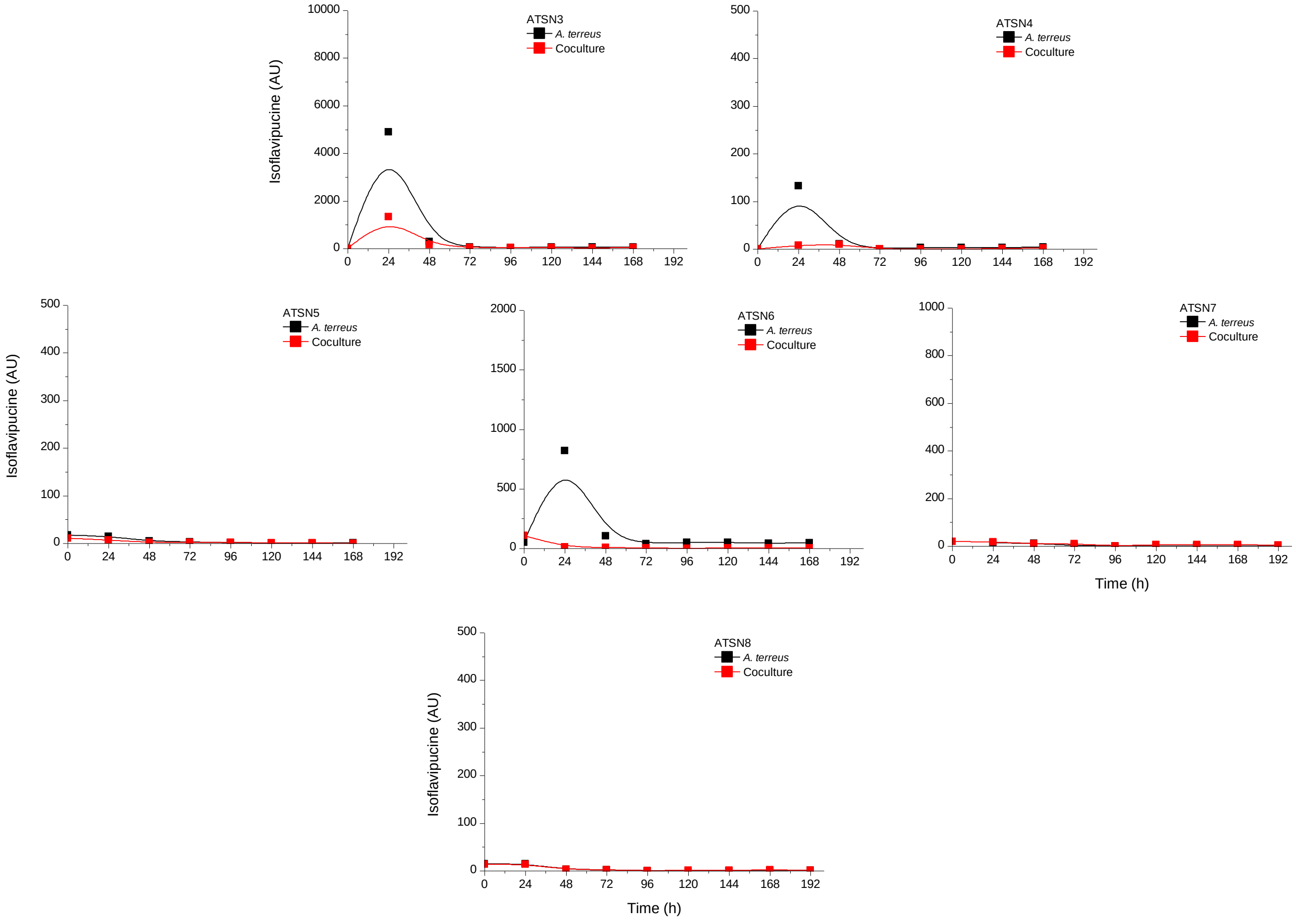

Fig. S29 Time courses of isoflavipucine production in the *Aspergillus terreus* and *Streptomyces noursei* co-cultures and the corresponding monoculture controls of *A. terreus*. AU-auxiliary units

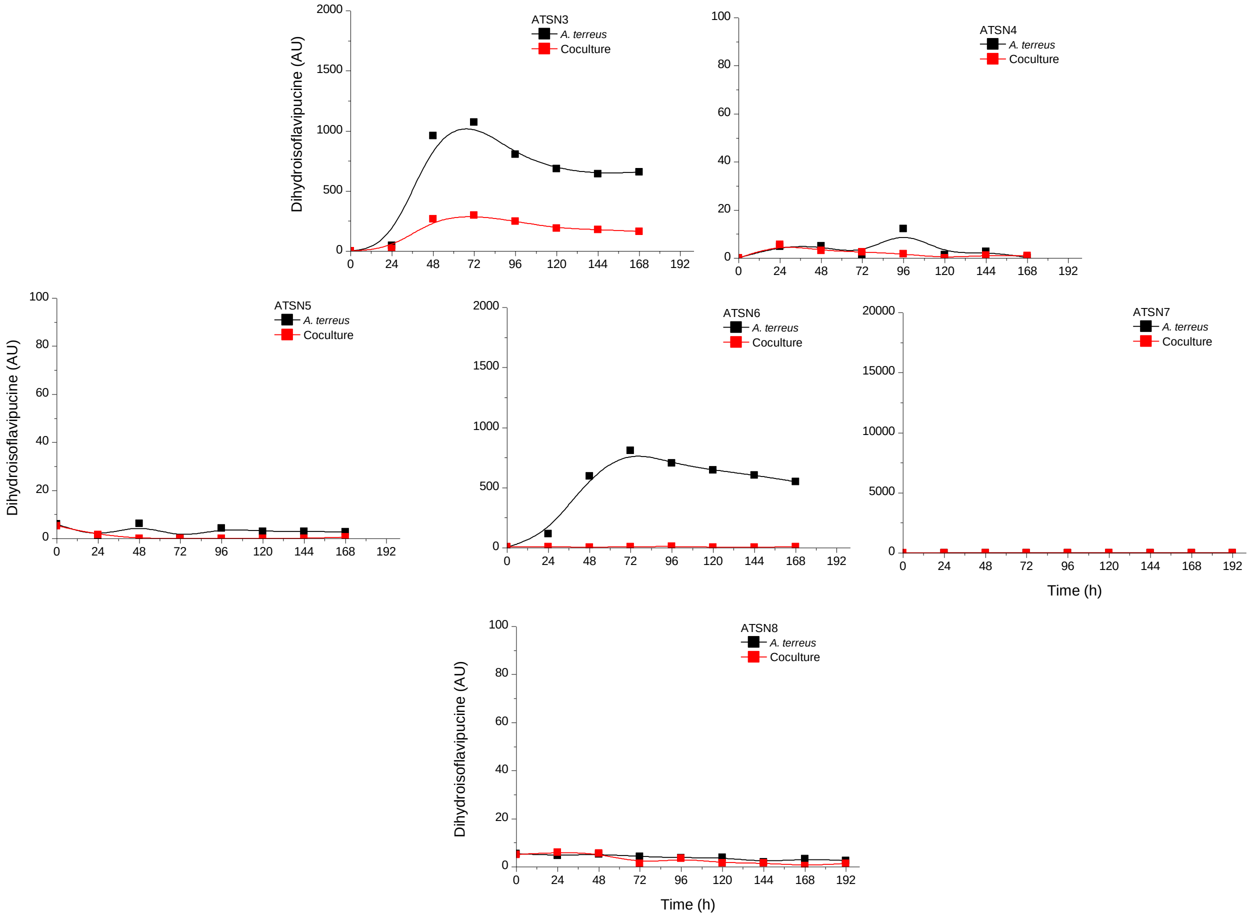

Fig. S30 Time courses of dihydroisoflavipucine production in the *Aspergillus terreus* and *Streptomyces noursei* co-cultures and the corresponding monoculture controls of *A. terreus*. AU-auxiliary units

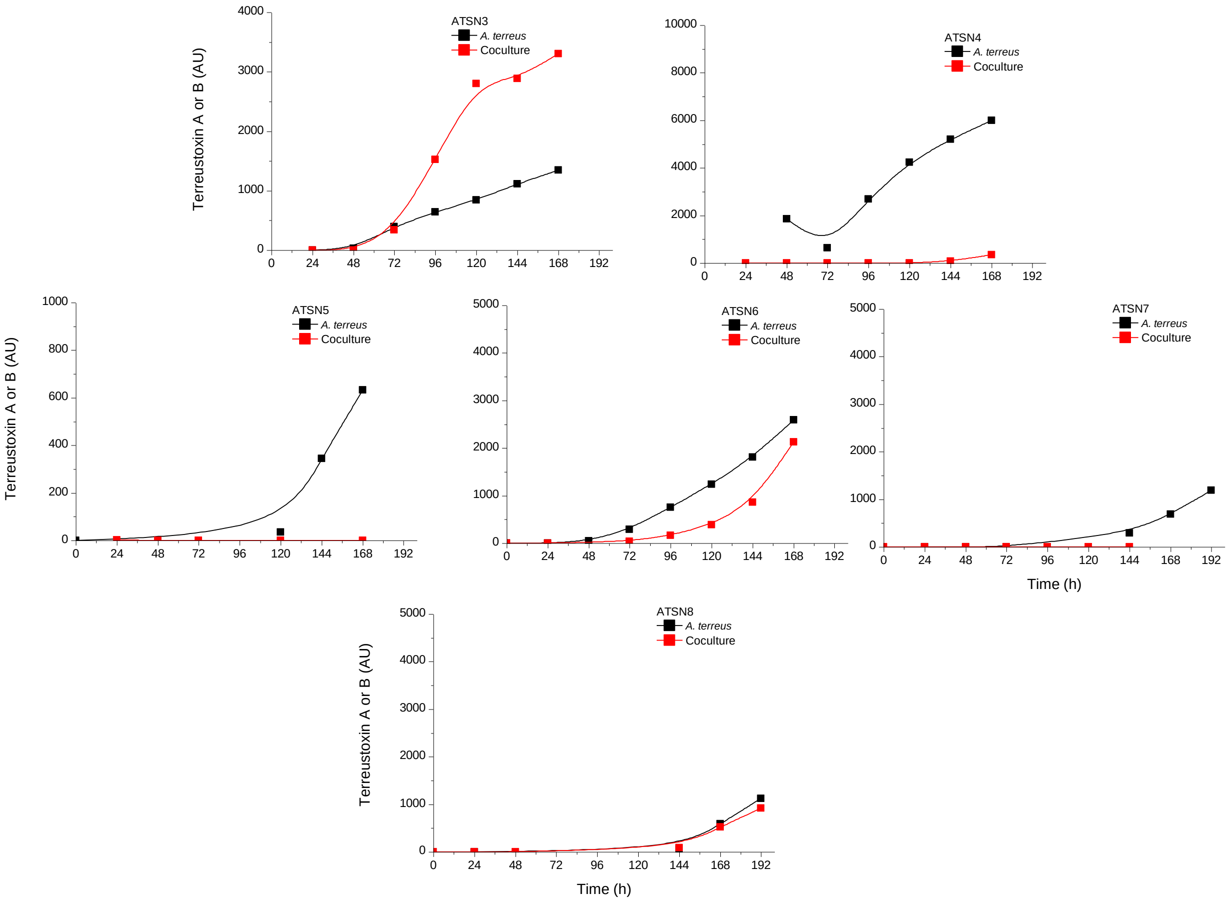

Fig. S31 Time courses of terreustoxin A or B production in the *Aspergillus terreus* and *Streptomyces noursei* co-cultures and the corresponding monoculture controls of *A. terreus*. AU-auxiliary units

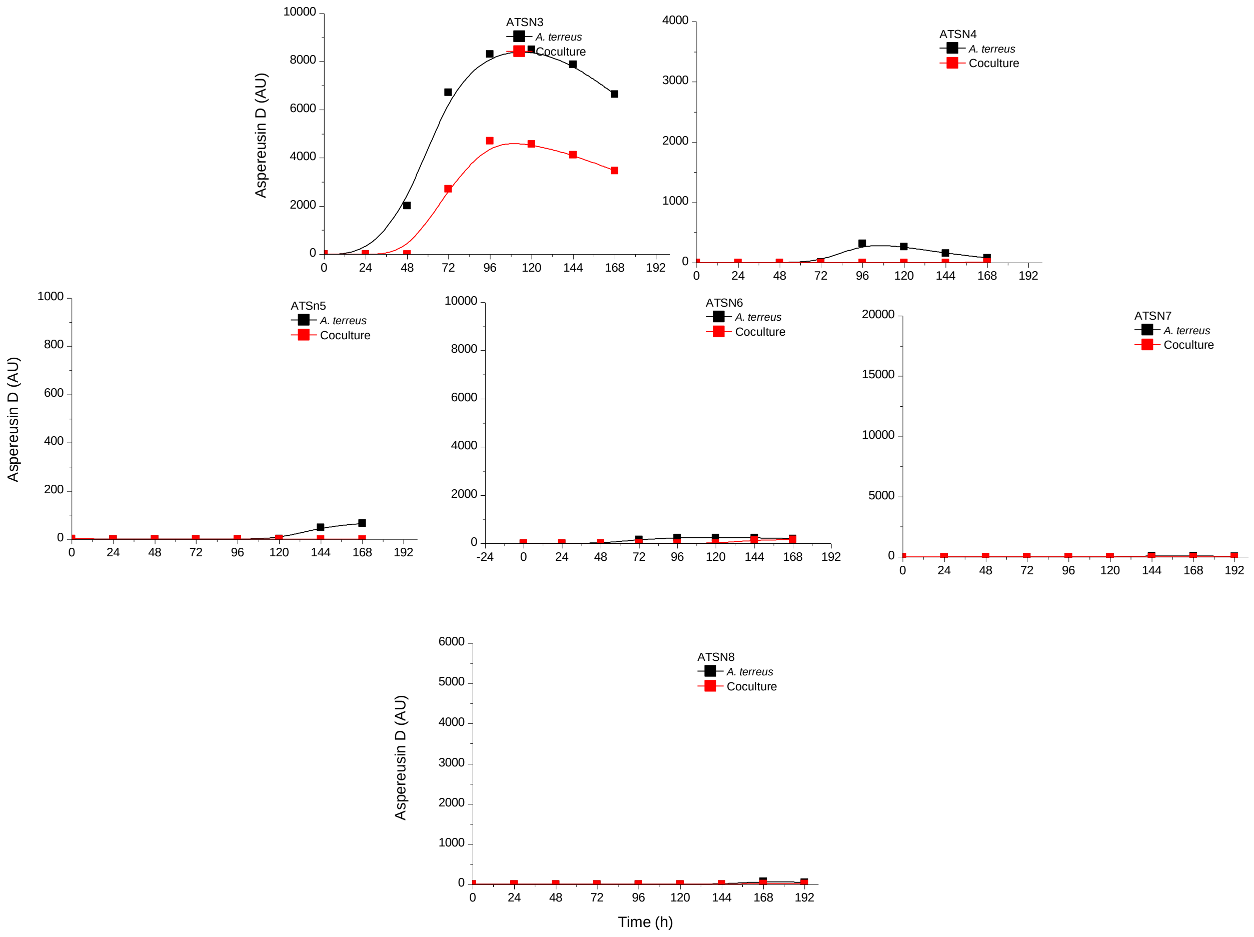

Fig. S32 Time courses of aspereusin D production in the *Aspergillus terreus* and *Streptomyces noursei* co-cultures and the corresponding monoculture controls of *A. terreus*. AU-auxiliary units

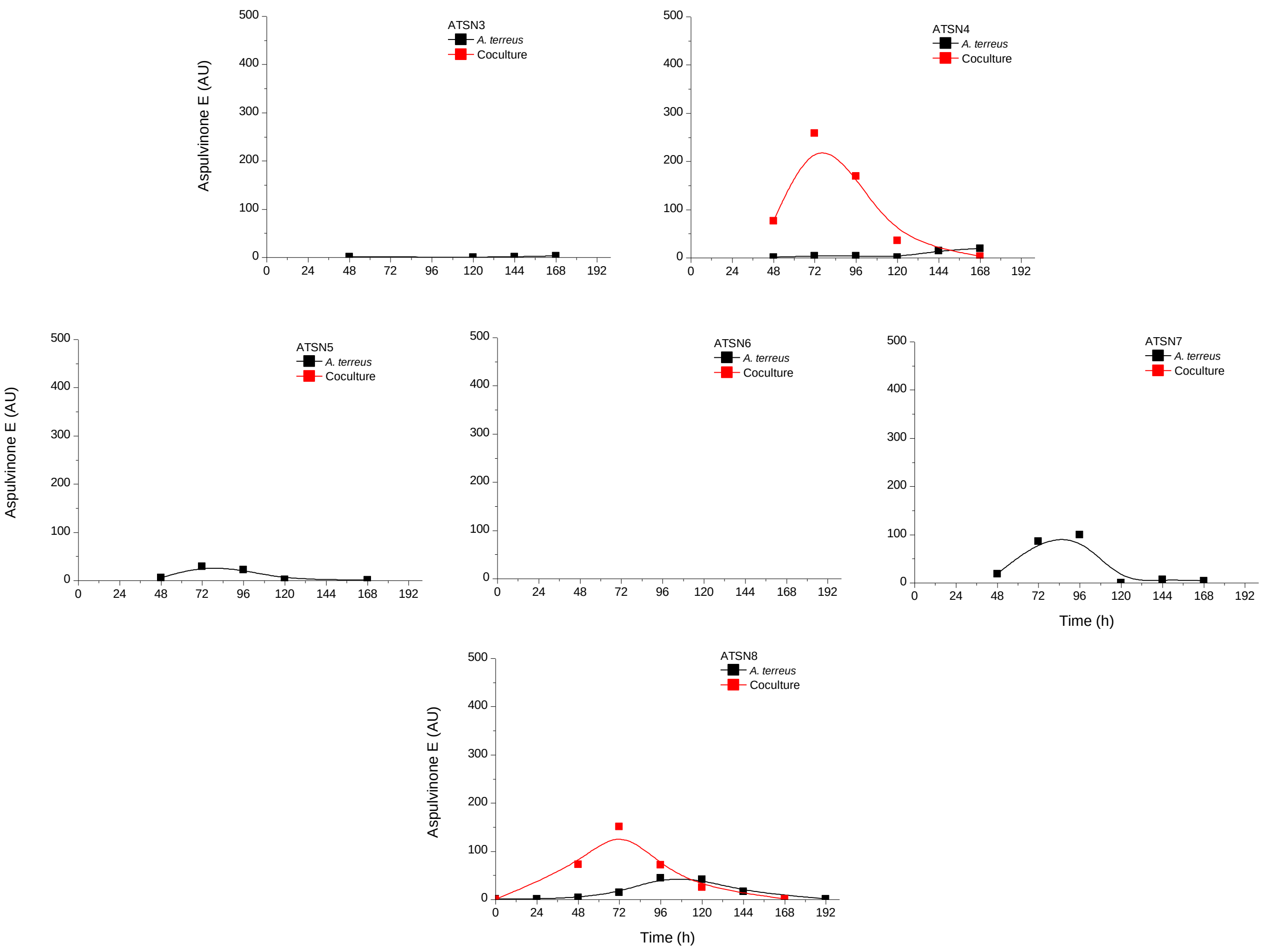

Fig. S33 Time courses of aspulvinone E production in the *Aspergillus terreus* and *Streptomyces noursei* co-cultures and the corresponding monoculture controls of *A. terreus*. AU-auxiliary units

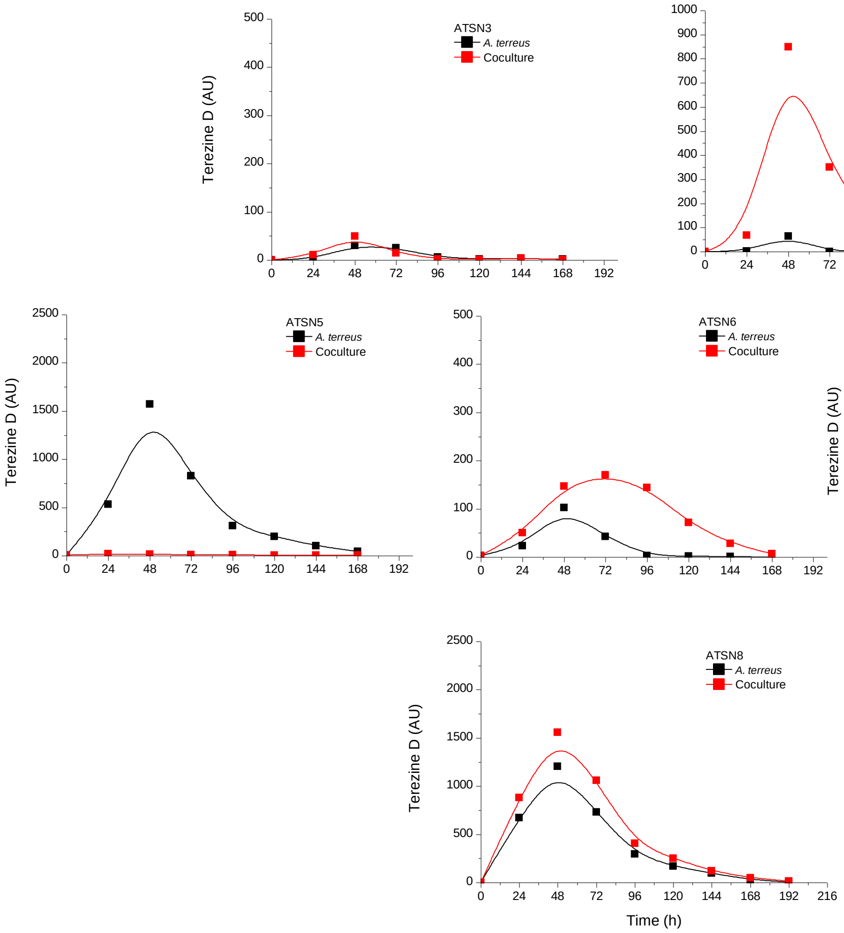

Fig. S34 Time courses of terezine D production in the *Aspergillus terreus* and *Streptomyces noursei* co-cultures and the corresponding monoculture controls of *A. terreus*. AU-auxiliary units

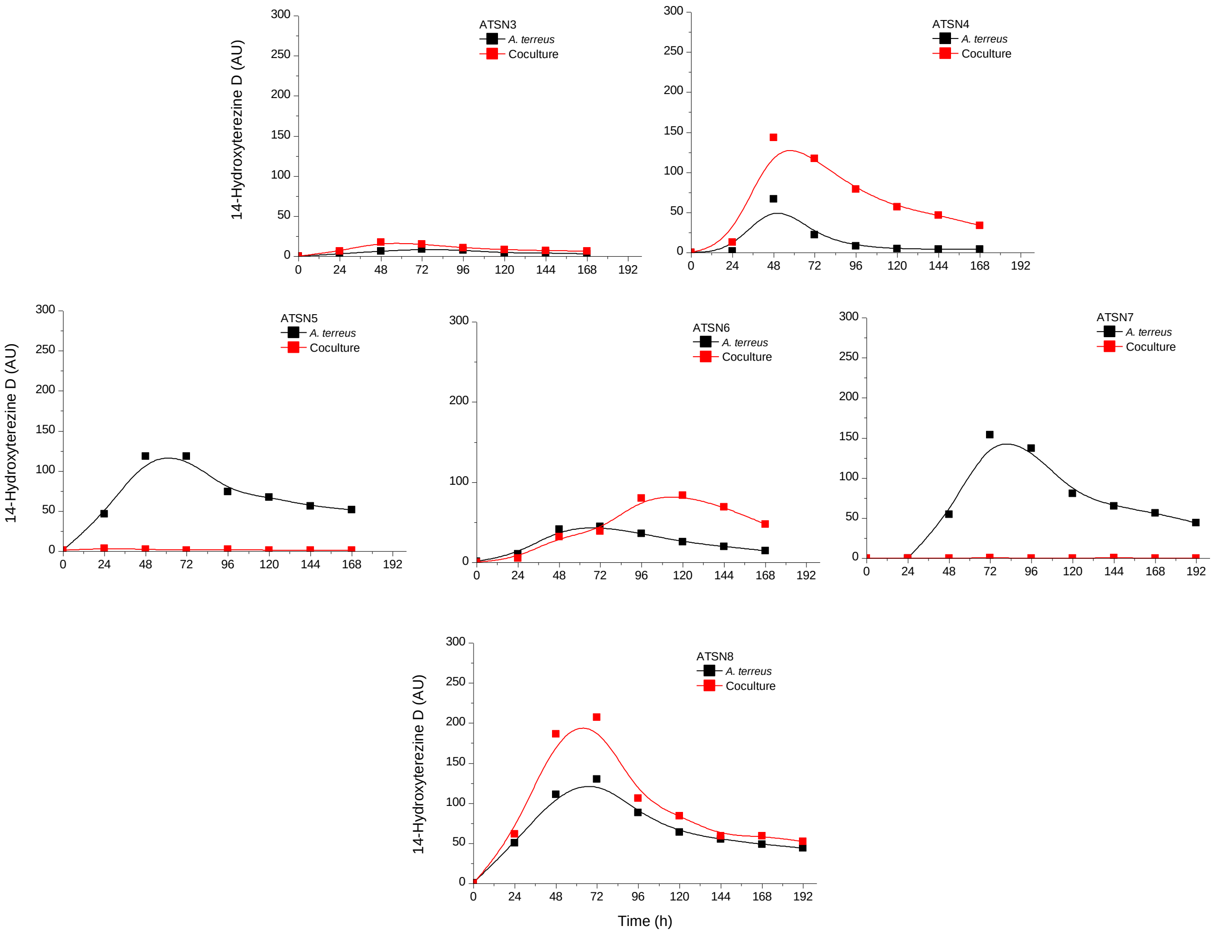

Fig. S35 Time courses of 14-hydroxyterezine D production in the *Aspergillus terreus* and *Streptomyces noursei* co-cultures and the corresponding monoculture controls of *A. terreus*. AU-auxiliary units

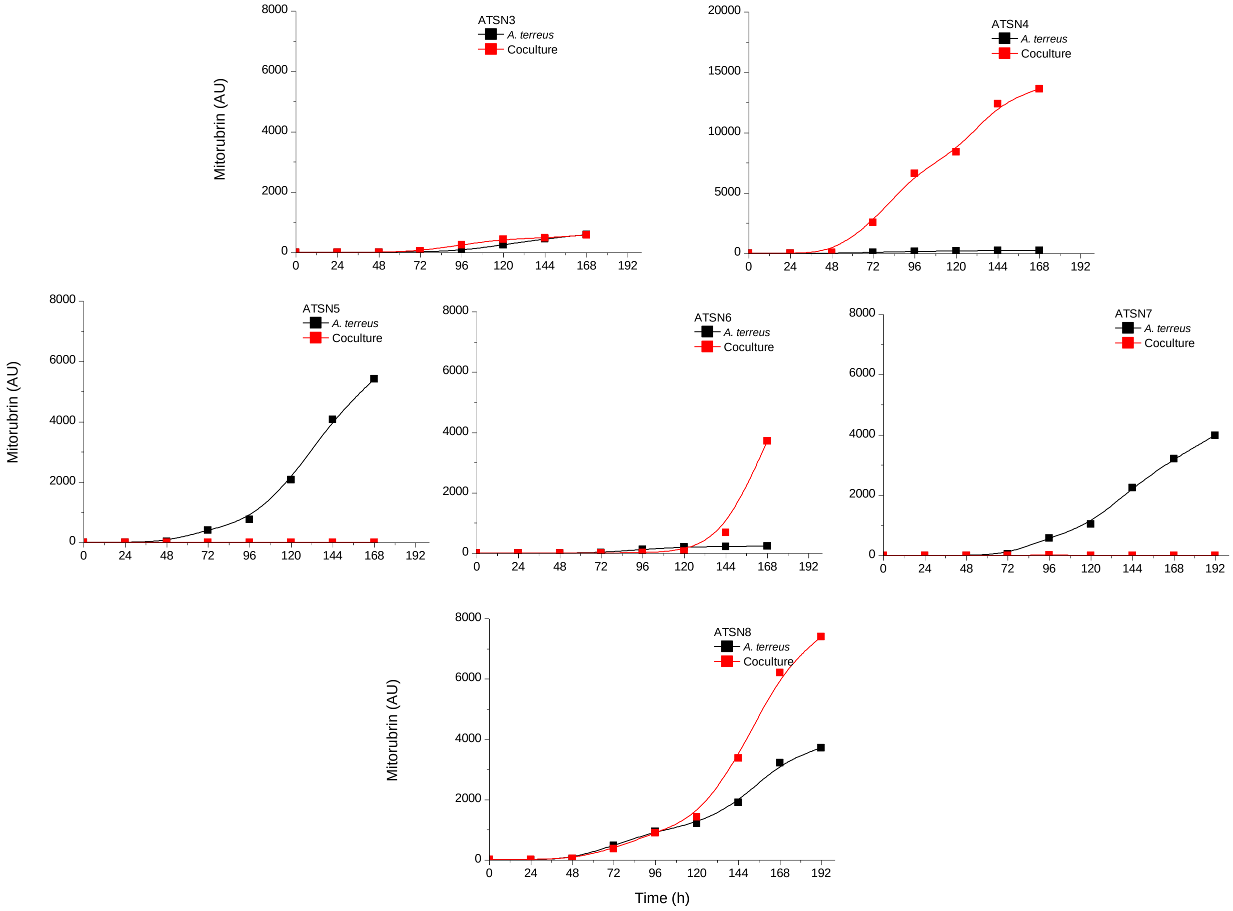

Fig. S36 Time courses of mitorubrin production in the *Aspergillus terreus* and *Streptomyces noursei* co-cultures and the corresponding monoculture controls of *A. terreus*. AU-auxiliary units

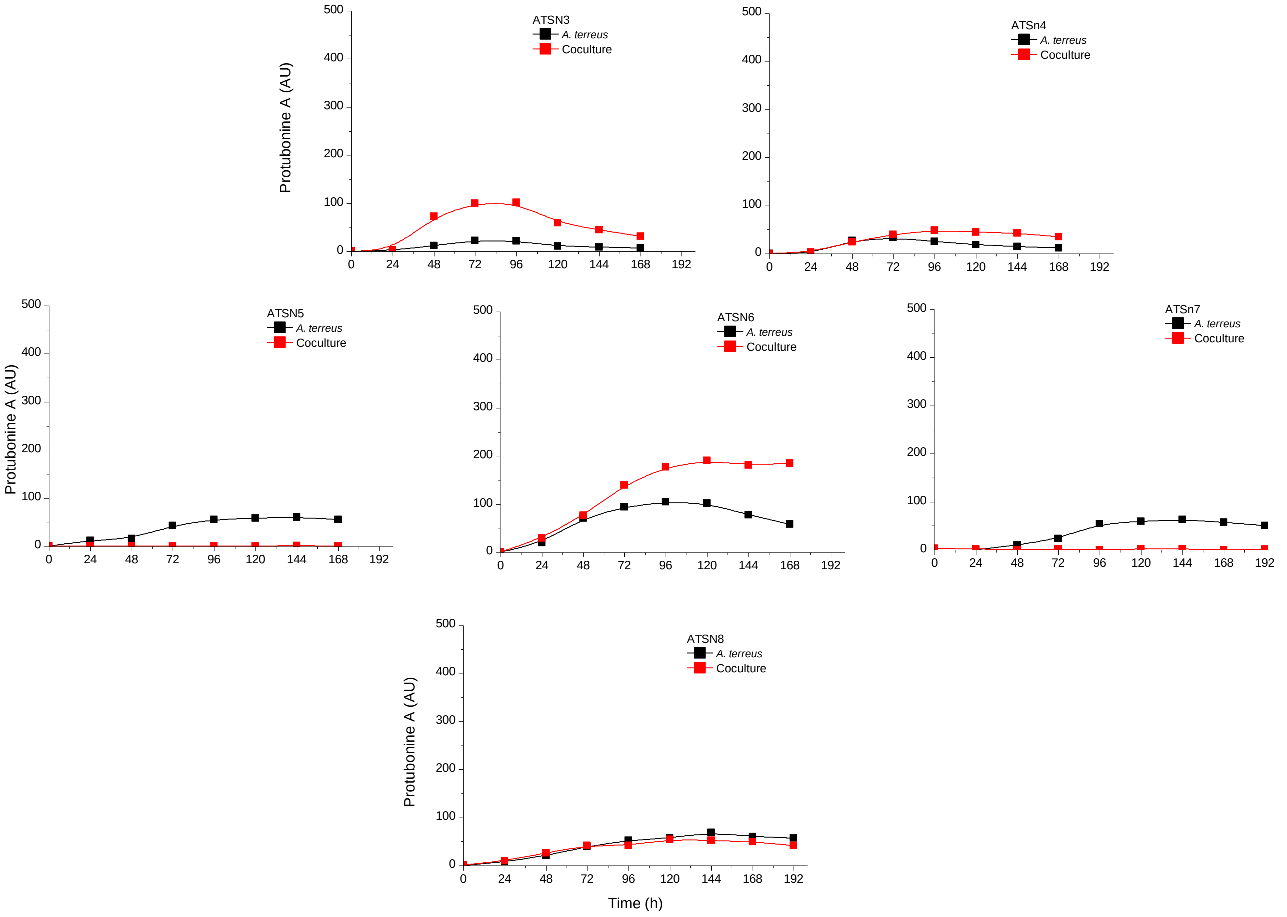

Fig. S37 Time courses of protubonine A production in the *Aspergillus terreus* and *Streptomyces noursei* co-cultures and the corresponding monoculture controls of *A. terreus*. AU-auxiliary units

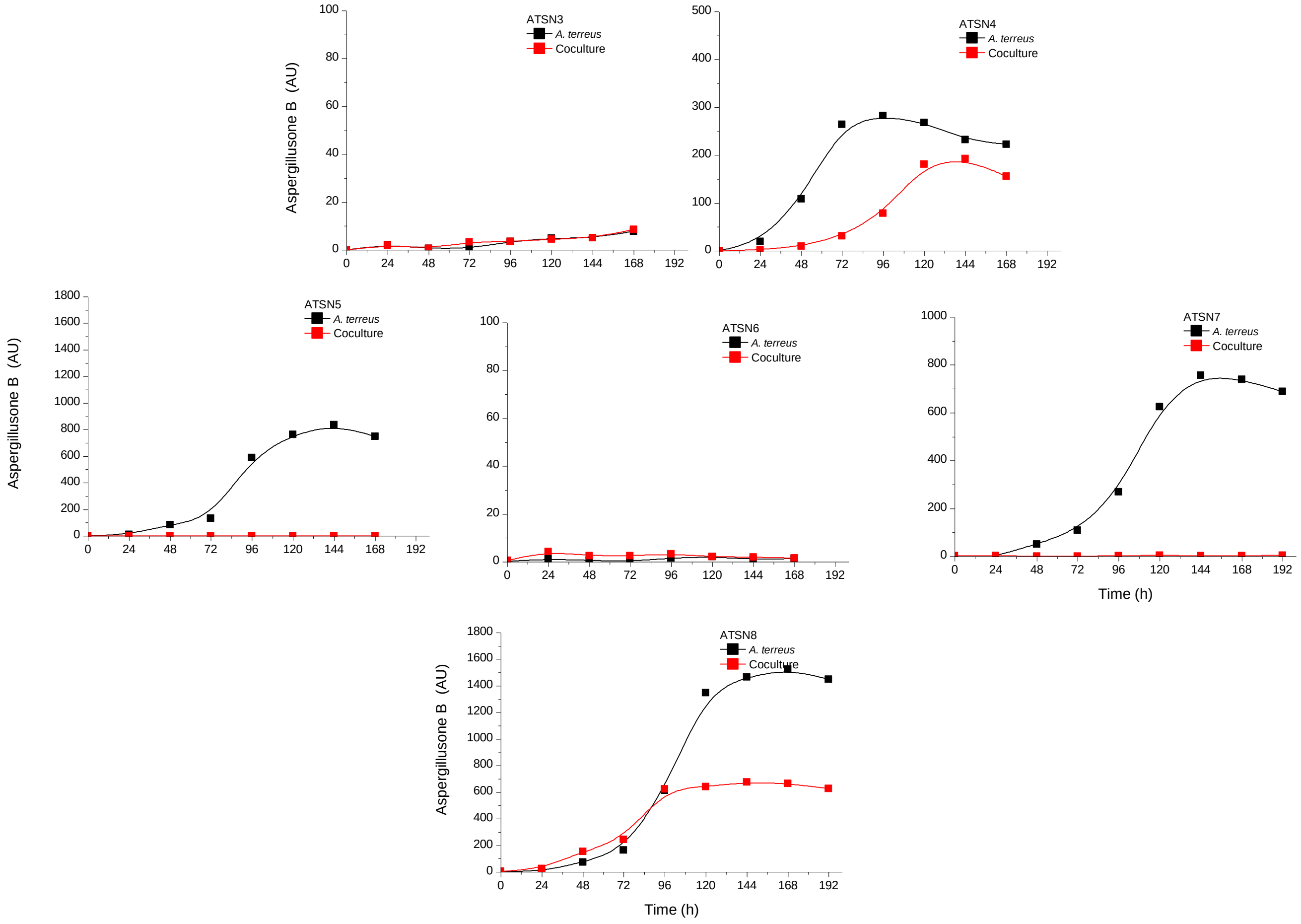

Fig. S38 Time courses of aspergillusone B production in the *Aspergillus terreus* and *Streptomyces noursei* co-cultures and the corresponding monoculture controls of *A. terreus*. AU-auxiliary units

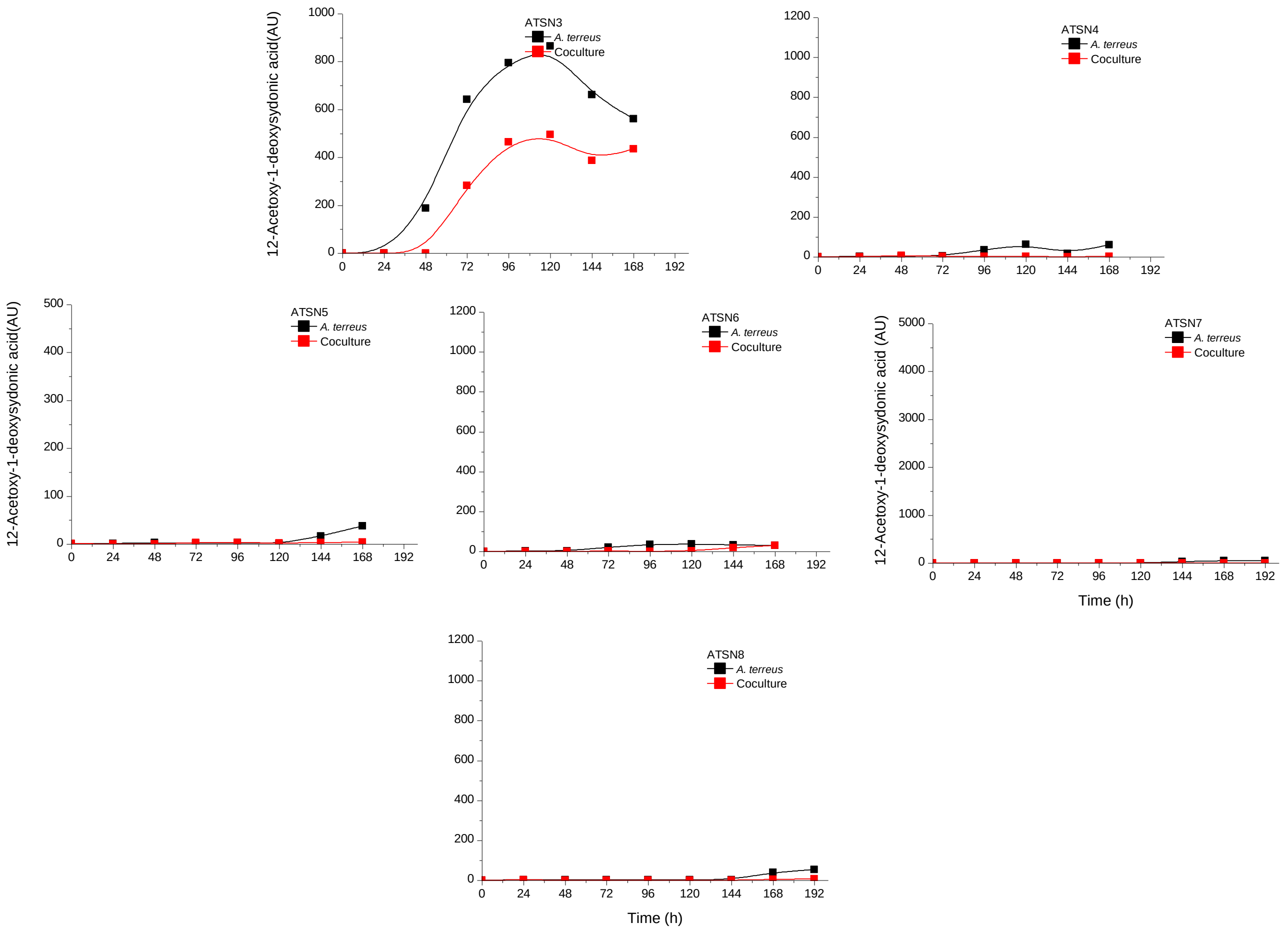

Fig. S39 Time courses of (-)-12-acetoxy-1-deoxysydonic acid production in the *Aspergillus terreus* and *Streptomyces noursei* co-cultures and the corresponding monoculture controls of *A. terreus*. AU-auxiliary units

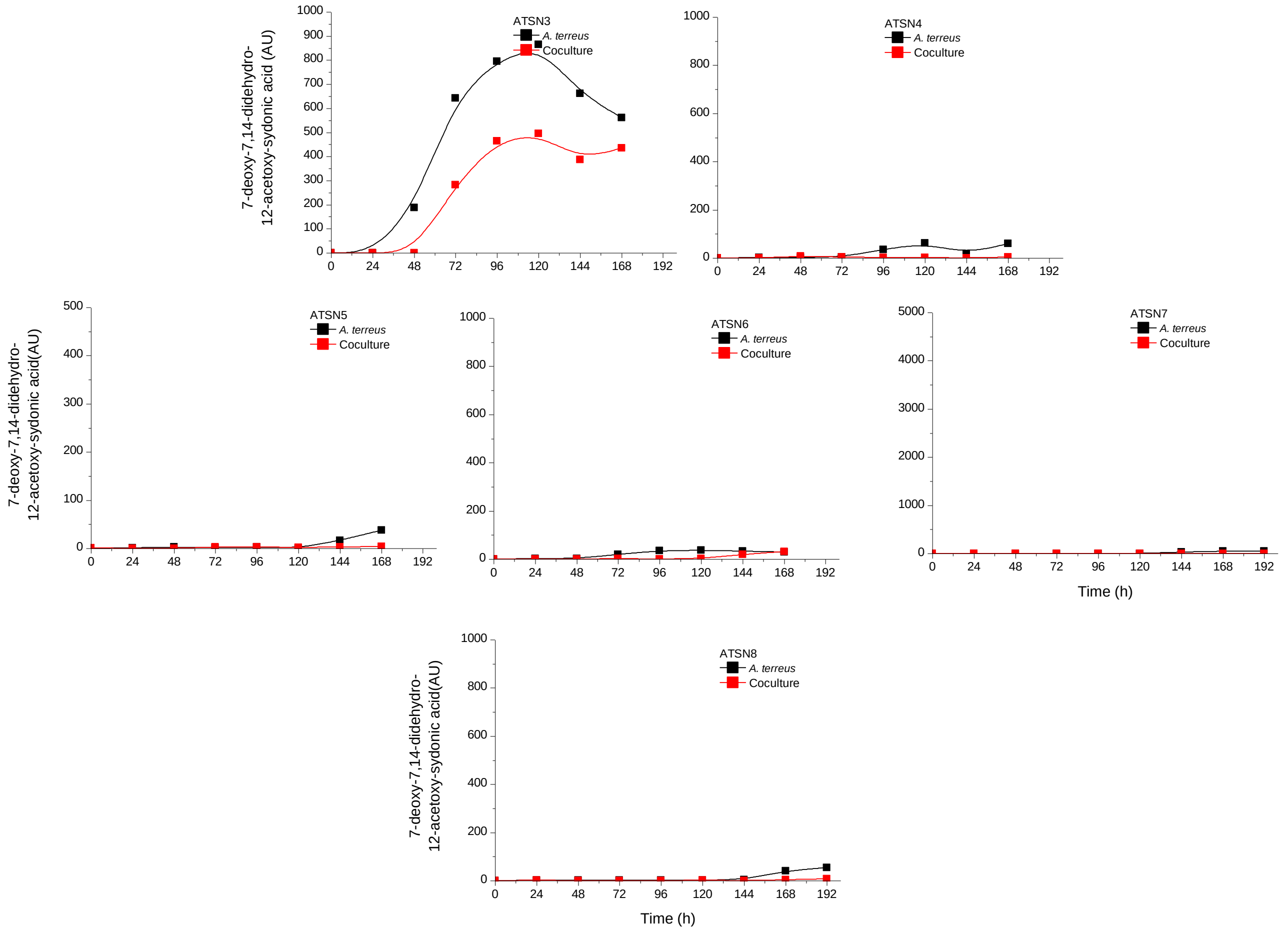

Fig. S40 Time courses of 7-deoxy-7,14-didehydro-12-acetoxysydonic acid production in the *Aspergillus terreus* and *Streptomyces noursei* co-cultures and the corresponding monoculture controls of *A. terreus*. AU-auxiliary units

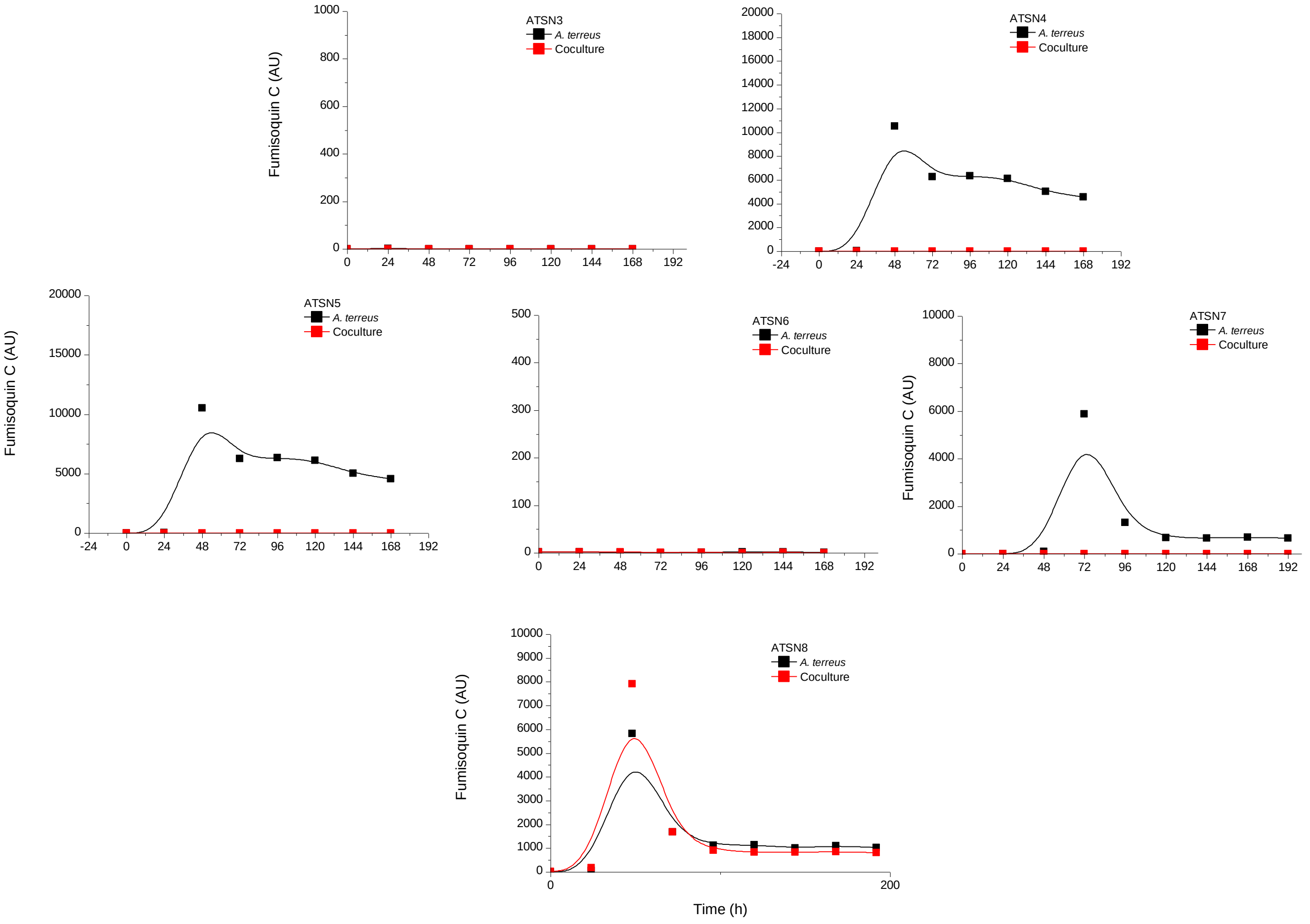

Fig. S41 Time courses of fumisoquin C production in the *Aspergillus terreus* and *Streptomyces noursei* co-cultures and the corresponding monoculture controls of *A. terreus*. AU-auxiliary units

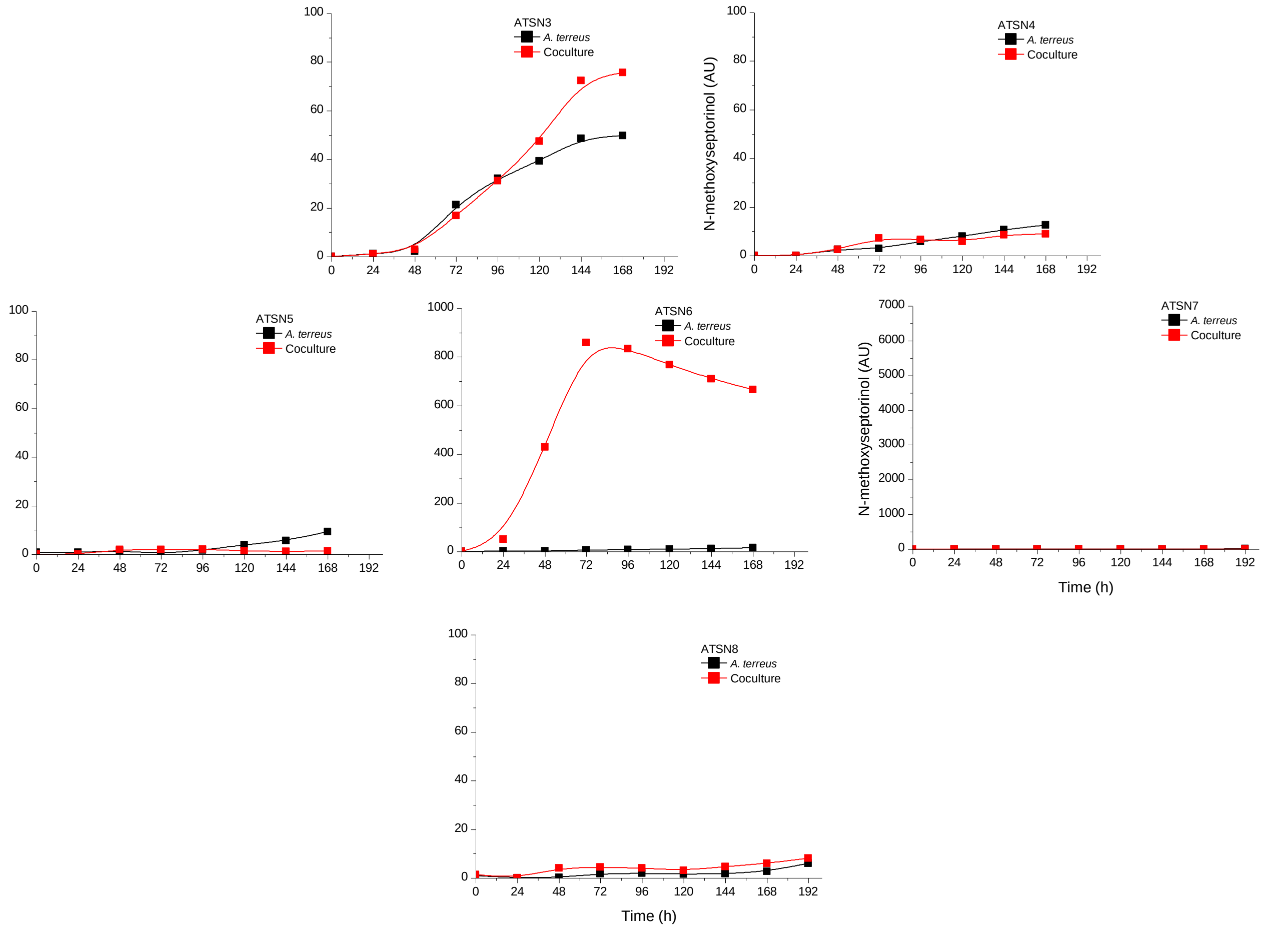

Fig. S42 Time courses of N-methoxyseptorinol production in the *Aspergillus terreus* and *Streptomyces noursei* co-cultures and the corresponding monoculture controls of *A. terreus*. AU-auxiliary units

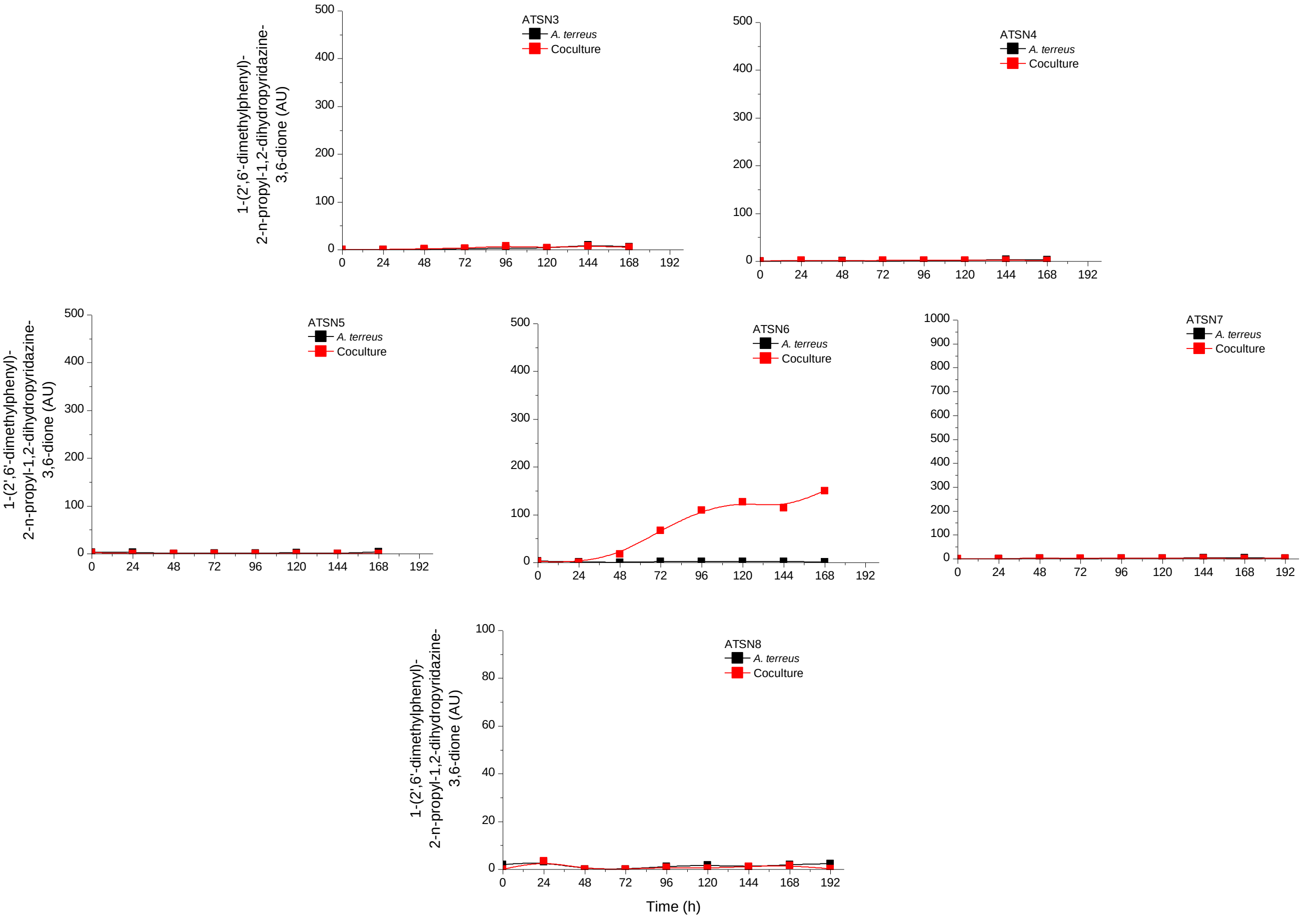

Fig. S43 Time courses of 1-(2',6'-dimethylphenyl)-2-n-propyl-1,2-dihydropyridazine-3,6-dione production in the *Aspergillus terreus* and *Streptomyces noursei* co-cultures and the corresponding monoculture controls of *A. terreus*. AU-auxiliary units

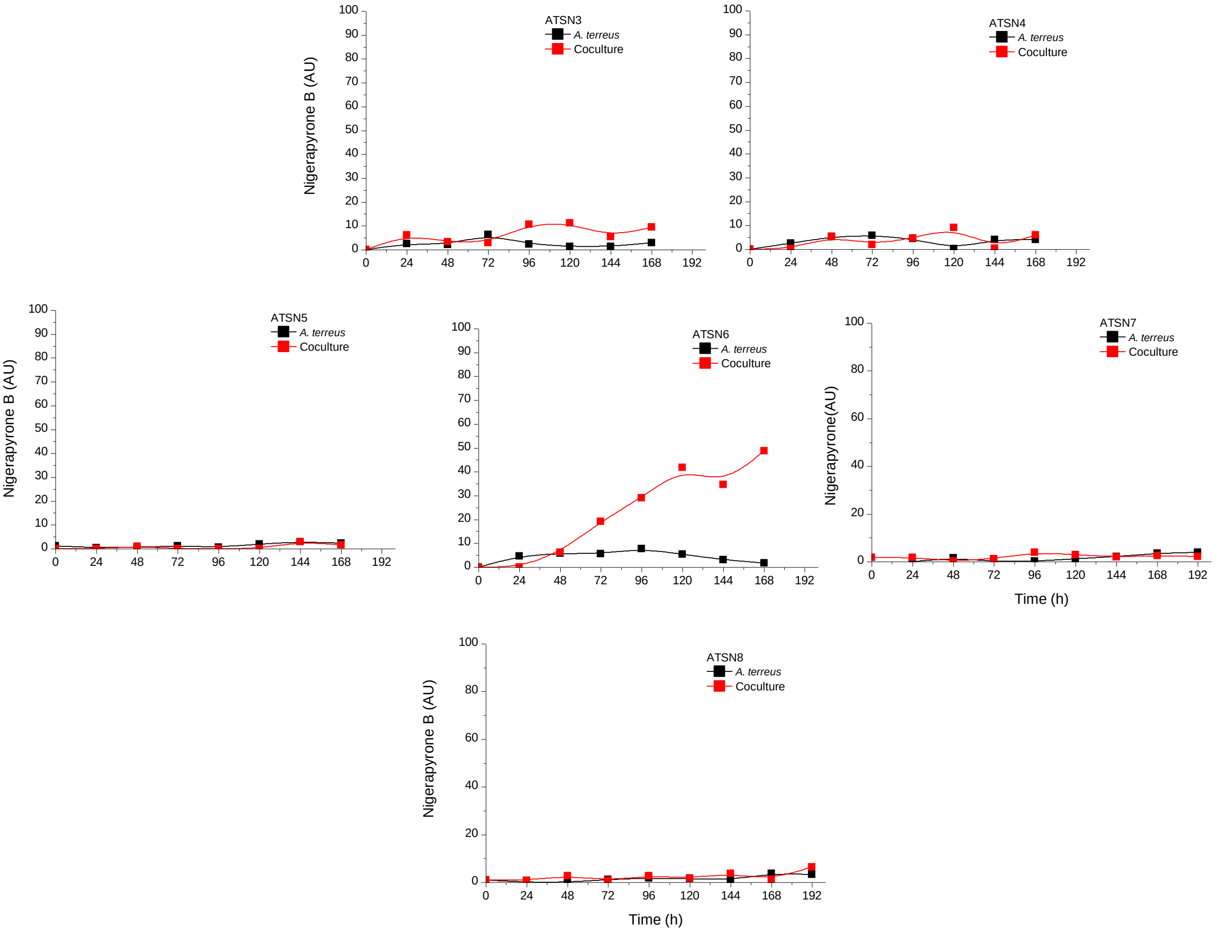

Fig. S44 Time courses of nigerapyrone B production in the *Aspergillus terreus* and *Streptomyces noursei* co-cultures and the corresponding monoculture controls of *A. terreus*. AU-auxiliary units

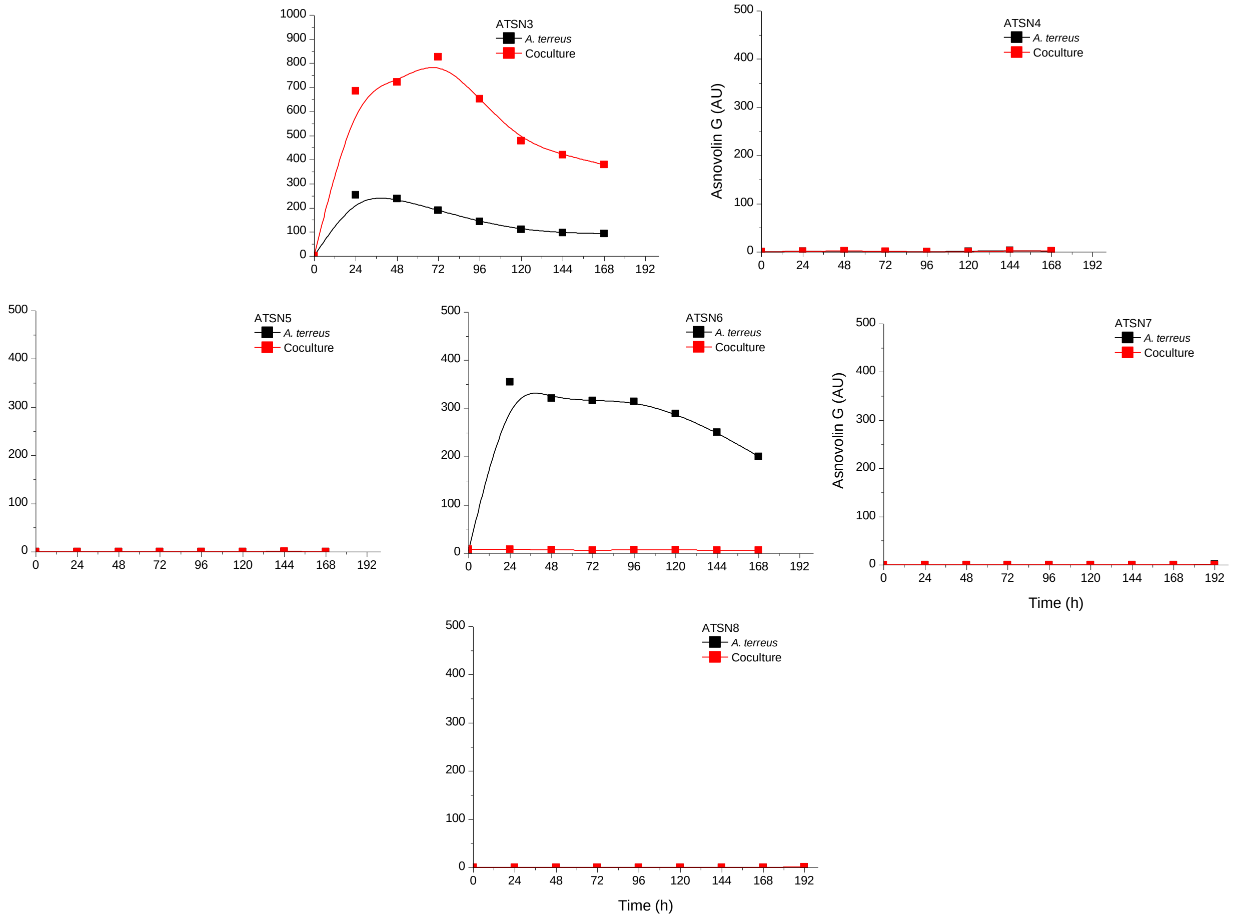

Fig. S45 Time courses of asnovolin G production in the *Aspergillus terreus* and *Streptomyces noursei* co-cultures and the corresponding monoculture controls of *A. terreus*. AU-auxiliary units

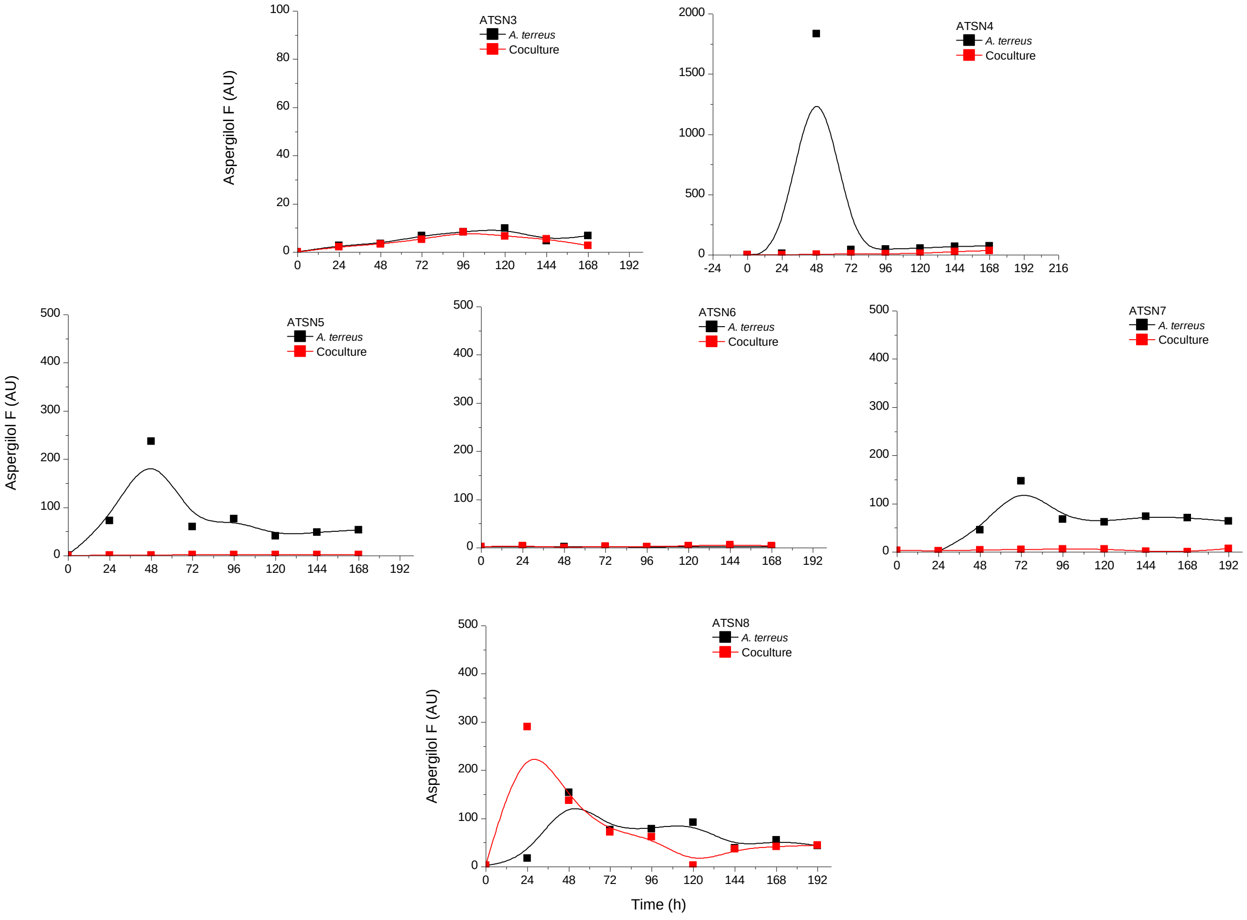

Fig. S46 Time courses of aspergilol F production in the *Aspergillus terreus* and *Streptomyces noursei* co-cultures and the corresponding monoculture controls of *A. terreus*. AU-auxiliary units

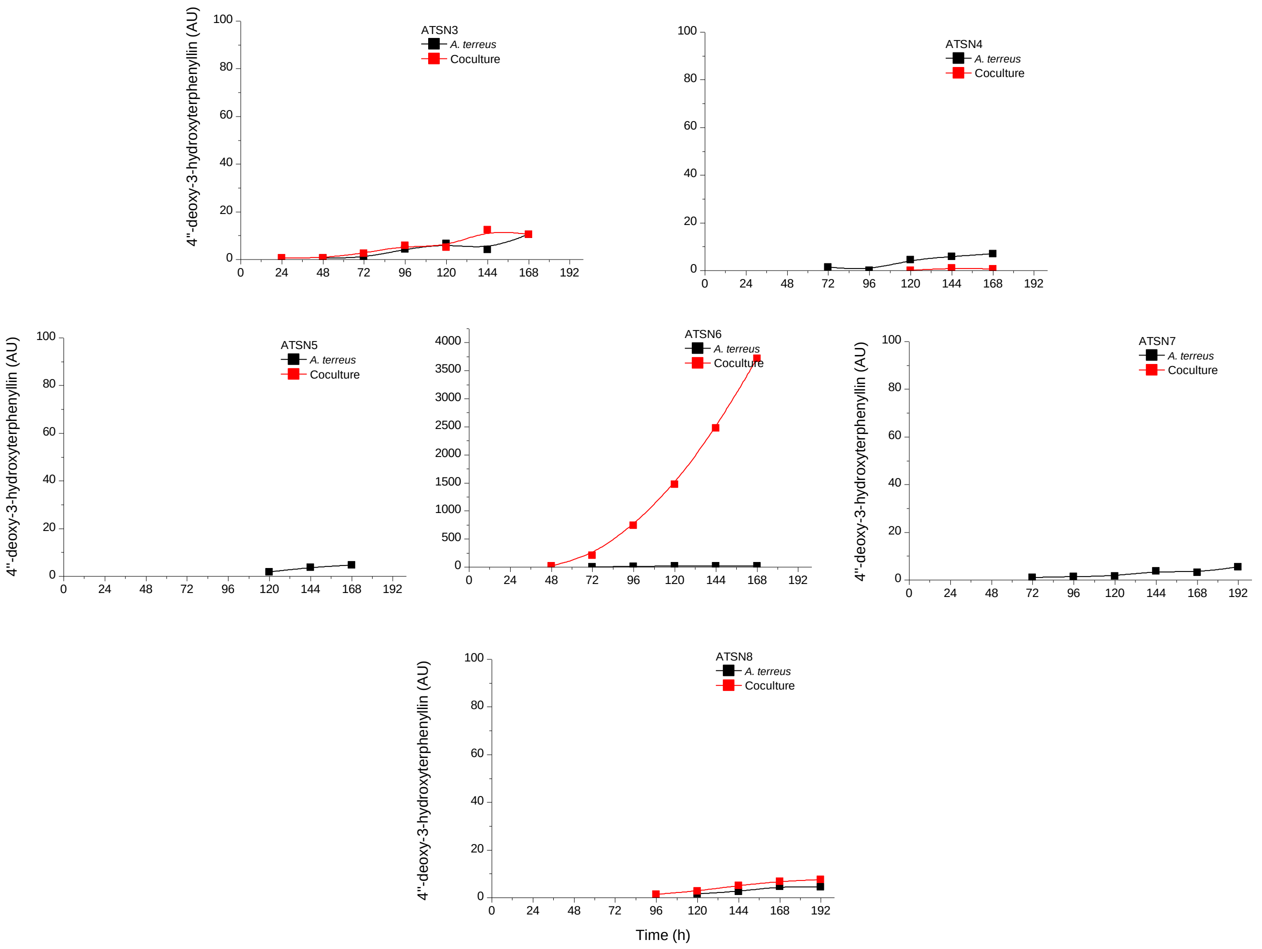

Fig. S47 Time courses of 4''-deoxy-3-hydroxyterphenyllin production in the *Aspergillus terreus* and *Streptomyces noursei* co-cultures and the corresponding monoculture controls of *A. terreus*. AU-auxiliary units

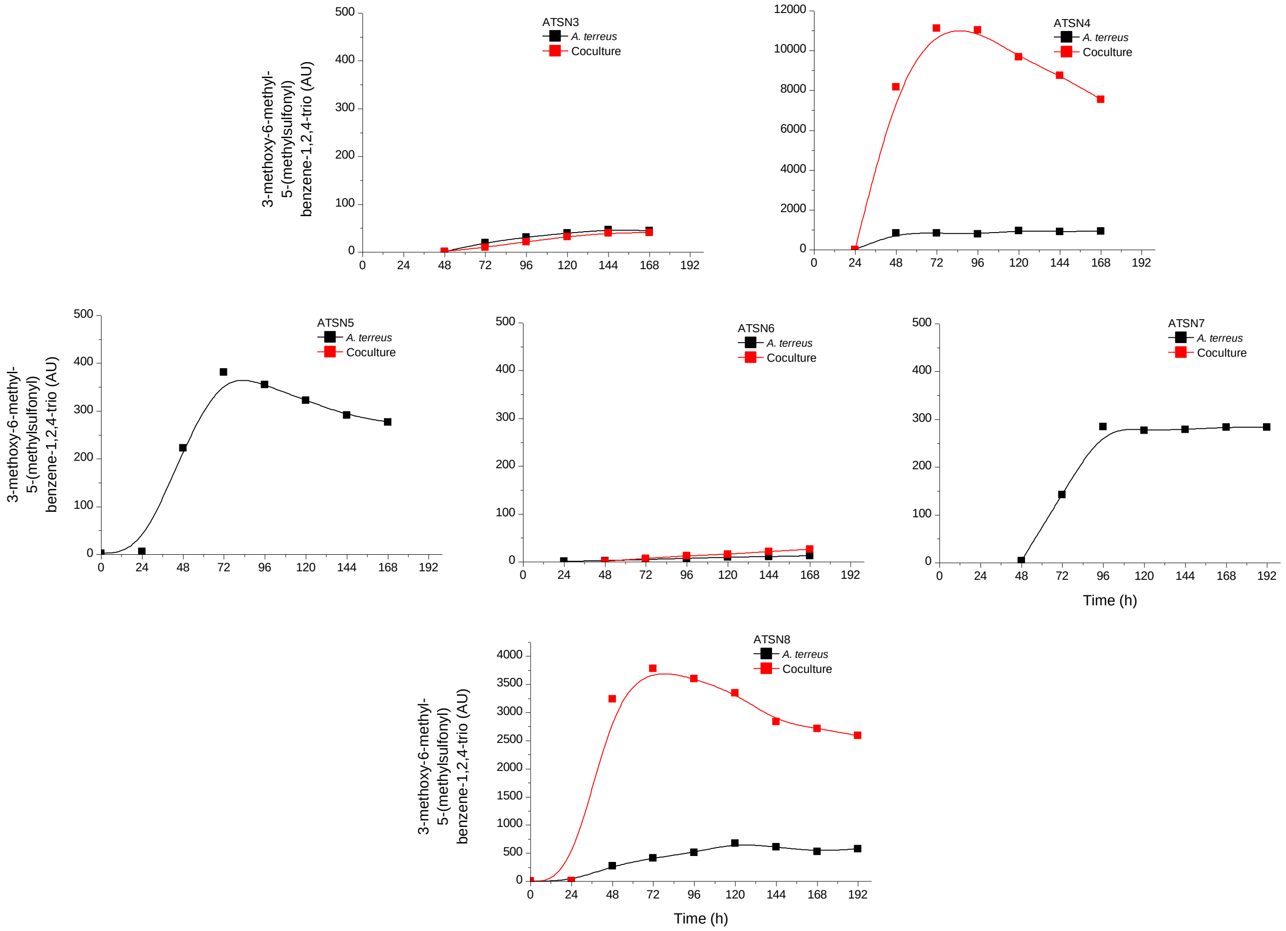

Fig. S48 Time courses of 3-methoxy-6-methyl-5-(methylsulfonyl)benzene-1,2,4-triol production in the *Aspergillus terreus* and *Streptomyces noursei* co-cultures and the corresponding monoculture controls of *A. terreus*. AU-auxiliary units

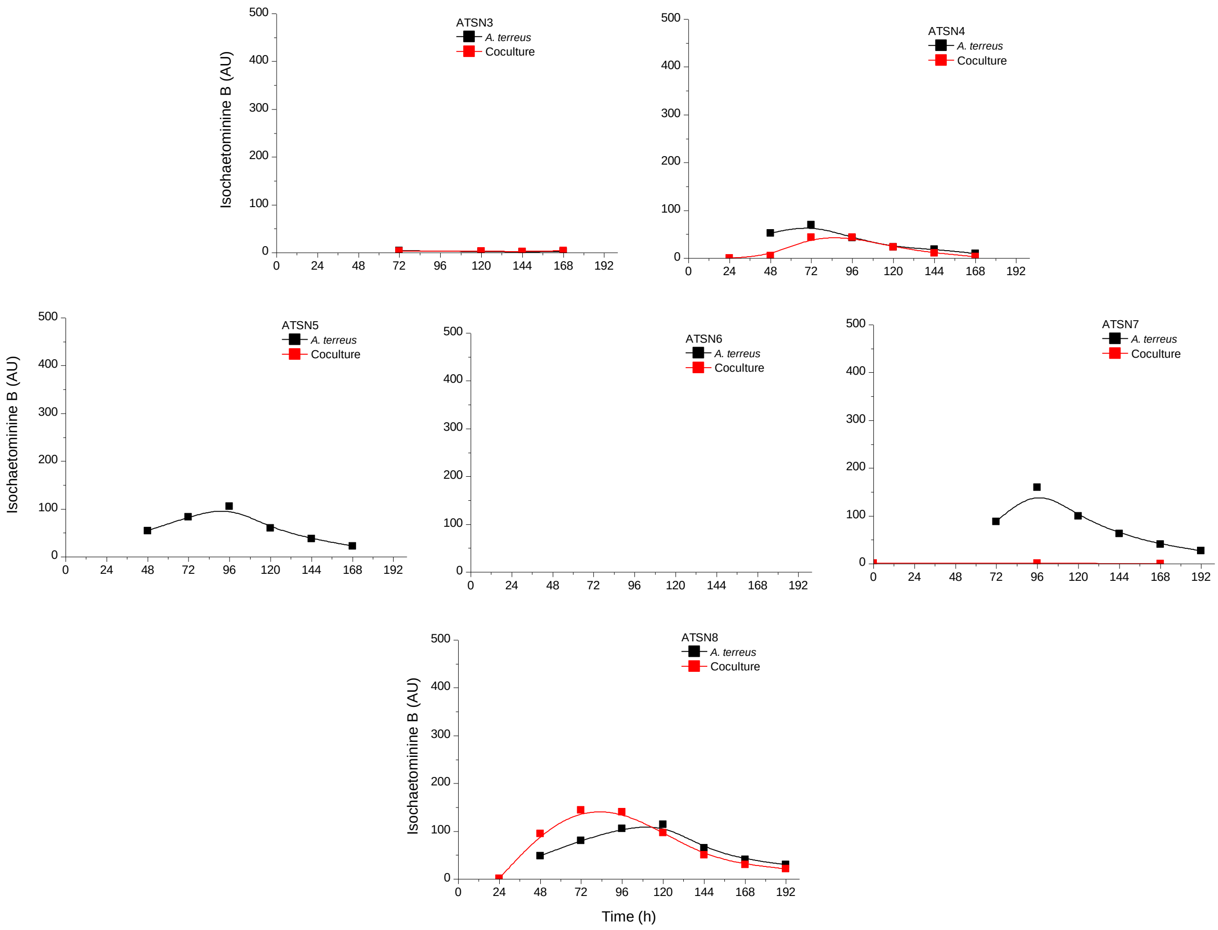

Fig. S49 Time courses of isochaetominine B production in the *Aspergillus terreus* and *Streptomyces noursei* co-cultures and the corresponding monoculture controls of *A. terreus*. AU-auxiliary units

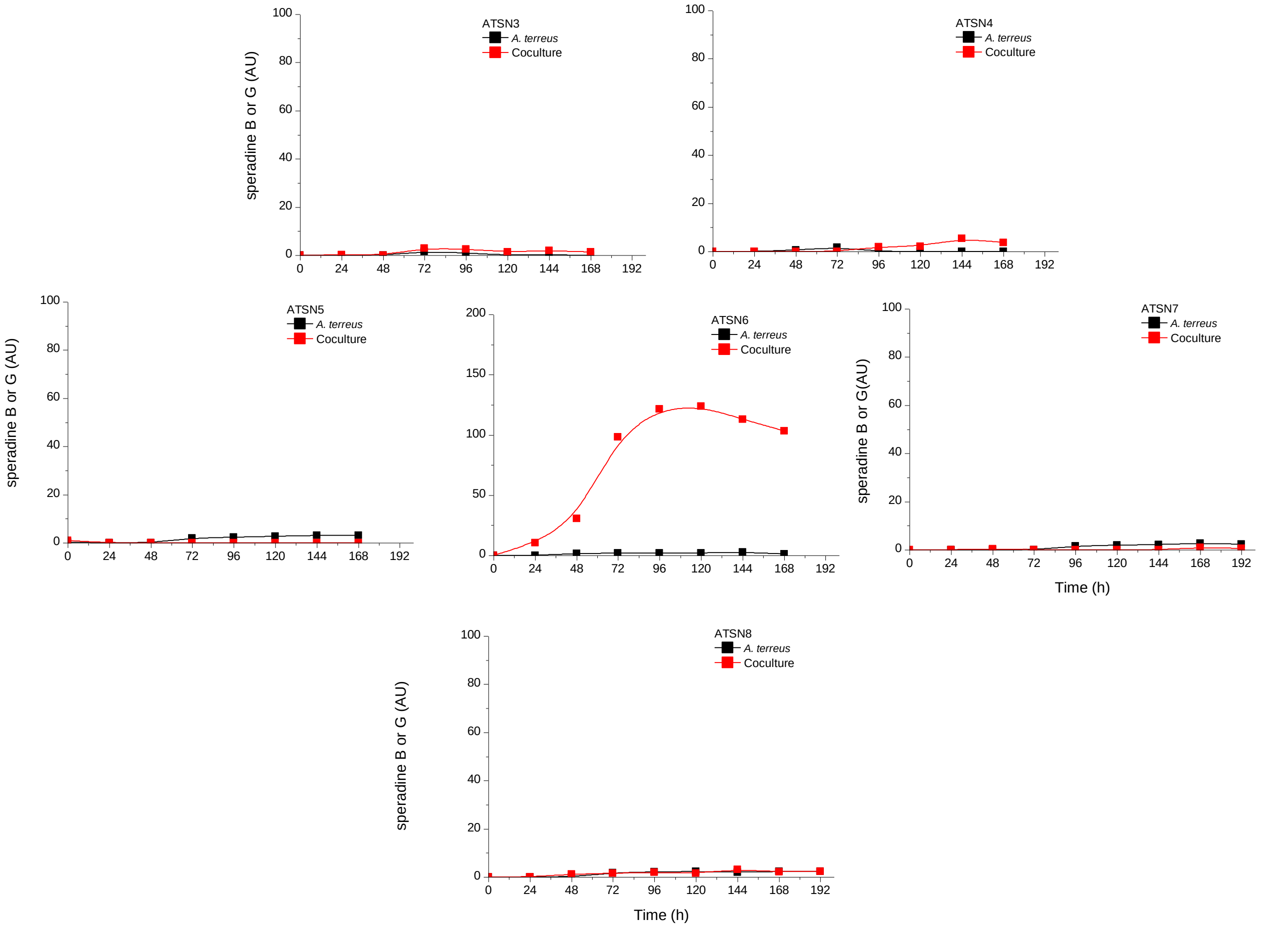

Fig. S50 Time courses of speradine B or G production in the *Aspergillus terreus* and *Streptomyces noursei* co-cultures and the corresponding monoculture controls of *A. terreus*. AU-auxiliary units

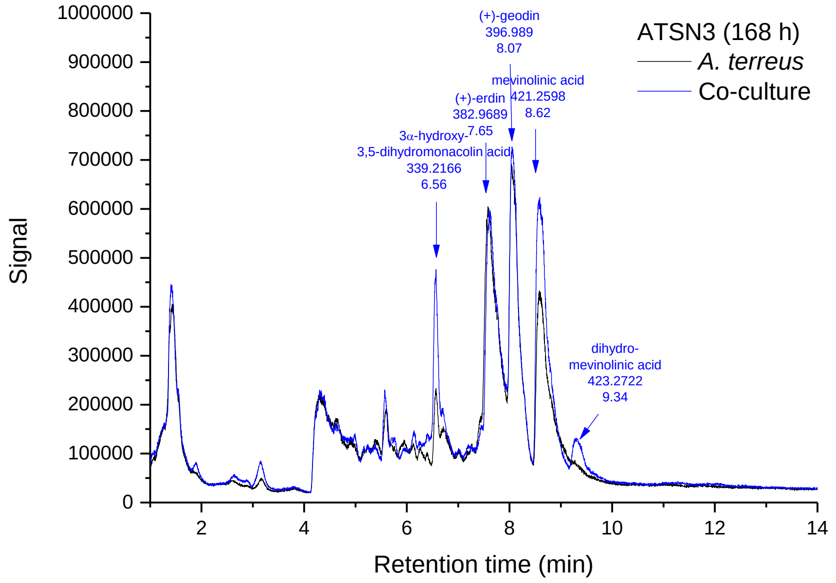

Fig. S51 Total ion chromatogram (ESI⁻) corresponding to ATSN3 experiment. The sample was collected at 168 h of the cultivation process.

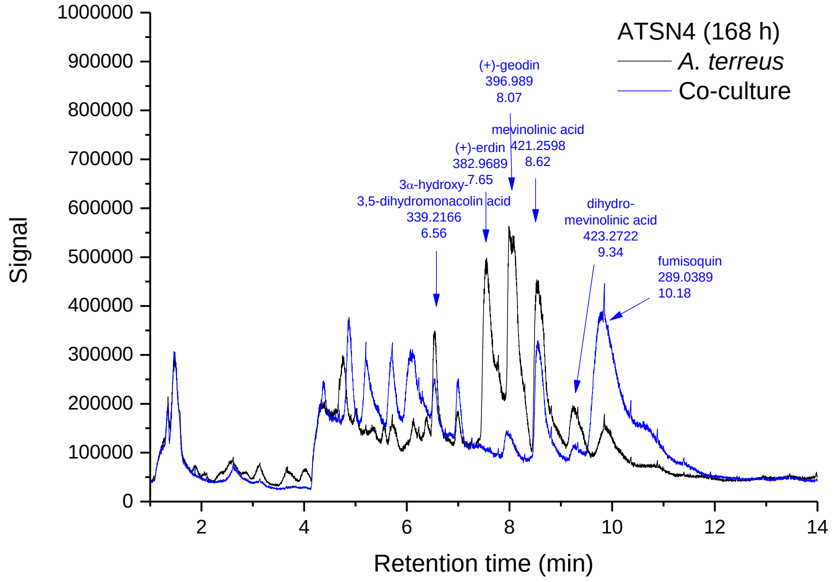

Fig. S52 Total ion chromatogram (ESI⁻) corresponding to ATSN4 experiment. The sample was collected at 168 h of the cultivation process.

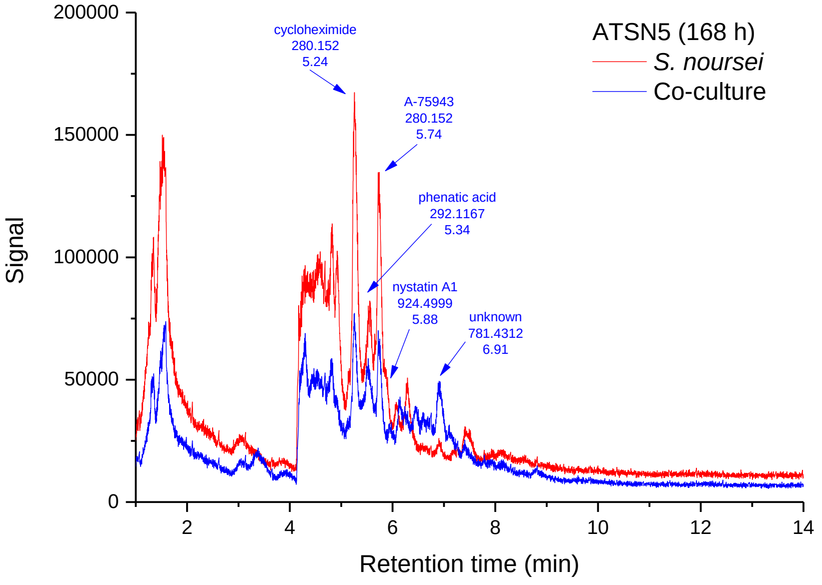

Fig. S53 Total ion chromatogram (ESI⁻) corresponding to ATSN5 experiment. The sample was collected at 168 h of the cultivation process.

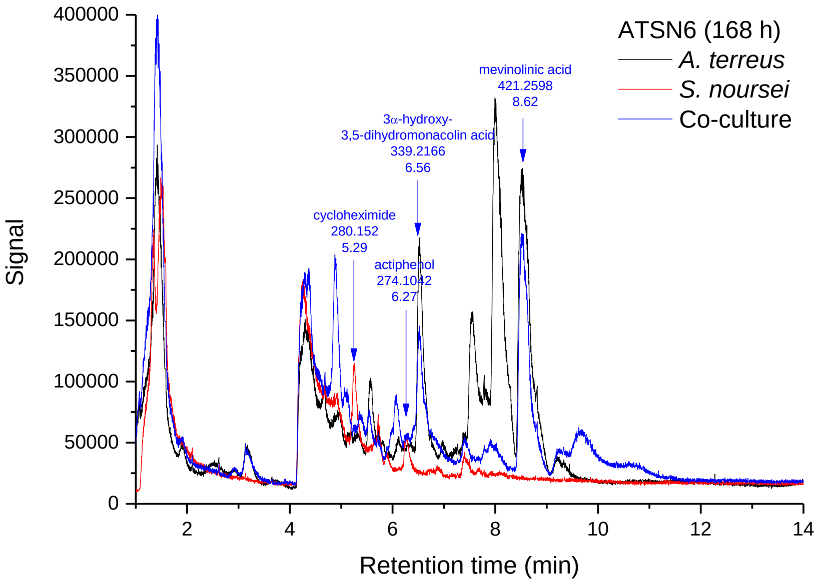

Fig. S54 Total ion chromatogram (ESI⁻) corresponding to ATSN6 experiment. The sample was collected at 168 h of the cultivation process.

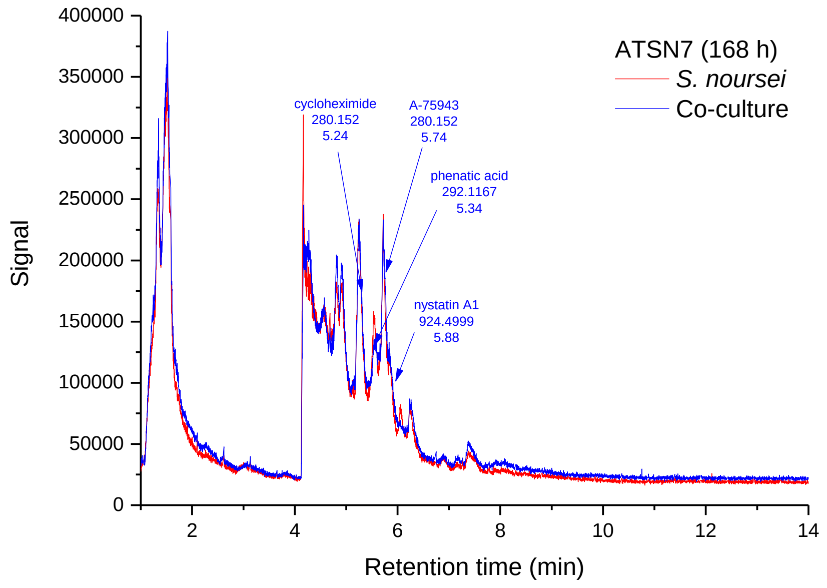

Fig. S55 Total ion chromatogram (ESI^{-}) corresponding to ATSN7 experiment. The sample was collected at 168 h of the cultivation process.

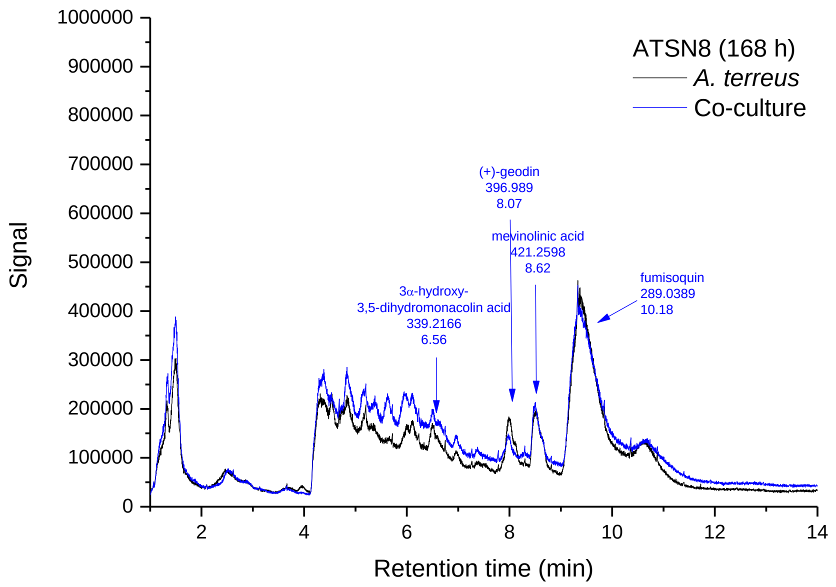

Fig. S56 Total ion chromatogram (ESI^{-}) corresponding to ATSN8 experiment. The sample was collected at 168 h of the cultivation process.
